# Pathobiont-triggered induction of goblet cell response drives regional susceptibility to inflammatory bowel disease

**DOI:** 10.1172/JCI201729

**Published:** 2026-02-17

**Authors:** Paige N. Spencer, Monica E. Brown, Erin P. Smith, Jiawei Wang, William Kim, Luisella Spiga, Naila Tasneem, Alan J. Simmons, Taewoo Kim, Yilin Yang, Yanwen Xu, Lin Zheng, James Ro, Harsimran Kaur, Seung Woo Kang, Matthew D. Helou, Mason A. Lee, Deronisha Arceneaux, Katherine D. Mueller, Ozge S. Kuddar, Mariah H. Harned, Jing Li, Amrita Banerjee, Nicholas O. Markham, Keith T. Wilson, Lori A. Coburn, Jeremy A. Goettel, Qi Liu, M. Kay Washington, Raphael H. Valdivia, Wenhan Zhu, Ken S. Lau

**Affiliations:** 1 Department of Cell and Developmental Biology and Program in Developmental Biology, Vanderbilt University, Nashville, Tennessee, USA.; 2 Epithelial Biology Center, Vanderbilt University Medical Center, Nashville, Tennessee, USA.; 3 Center for Computational Systems Biology, Vanderbilt University, Nashville, Tennessee, USA.; 4 Department of Integrative Immunobiology, Duke University School of Medicine, Durham, North Carolina, USA.; 5 Department of Pathology, Microbiology and Immunology and; 6 Vanderbilt Institute for Infection, Immunology and Inflammation, Vanderbilt University Medical Center, Nashville, Tennessee, USA.; 7 Department of Molecular Genetics and Microbiology, Duke University School of Medicine, Durham, North Carolina, USA.; 8 Department of Medicine, Division of Gastroenterology, Hepatology and Nutrition, Vanderbilt University Medical Center, Nashville, Tennessee, USA.; 9 Department of Veterans Affairs, Tennessee Valley Healthcare System, Nashville, Tennessee, USA.; 10 Center for Mucosal Inflammation and Cancer, Vanderbilt University Medical Center, Nashville, Tennessee, USA.; 11 Program in Cancer Biology, Vanderbilt University School of Medicine, Nashville, Tennessee, USA.; 12 Department of Biostatistics and Center for Quantitative Sciences, and; 13 Department of Surgery, Vanderbilt University Medical Center, Nashville, Tennessee, USA.

**Keywords:** Cell biology, Gastroenterology, Inflammation, Bacterial infections, Inflammatory bowel disease

## Abstract

The gastrointestinal tract varies in structure and function by region, yet the drivers of region-specific inflammatory disease remain elusive. Here, a TNF-overexpressing murine model (*Tnf*^ΔARE/+^) of Crohn’s disease (CD) was used to investigate how pathobionts interact with host immune susceptibilities to drive region-specific disease. We identified the pathobiont *Chlamydia muridarum*, an intracellular bacterium and murine counterpart to the human sexually transmitted *C*. *trachomatis*, as a necessary and sufficient trigger for disease manifestation in the proximal/ascending colon, a common site of CD. In genetically susceptible hosts, pathobiont-triggered proximal colonic inflammation is driven by goblet cell responses, including tryptophan metabolism via indoleamine 2,3-dioxygenase 1 (IDO1). Our findings translate to human disease, where we demonstrate upregulation of epithelia-derived IDO1 in actively inflamed ascending colon specimens, but not actively inflamed terminal ileum specimens, of patients with CD. Our findings mechanistically reveal how genetic and microbial factors drive the manifestation of disease in a region-specific manner and provide a unique model to study CD specific to the ascending colon.

## Abstract 2

Chlamydia and genetic risk combine to trigger Crohn’s disease-like inflammation in the proximal colon, revealing why disease occurs there, opening doors to targeted treatments.

## Graphical abstract

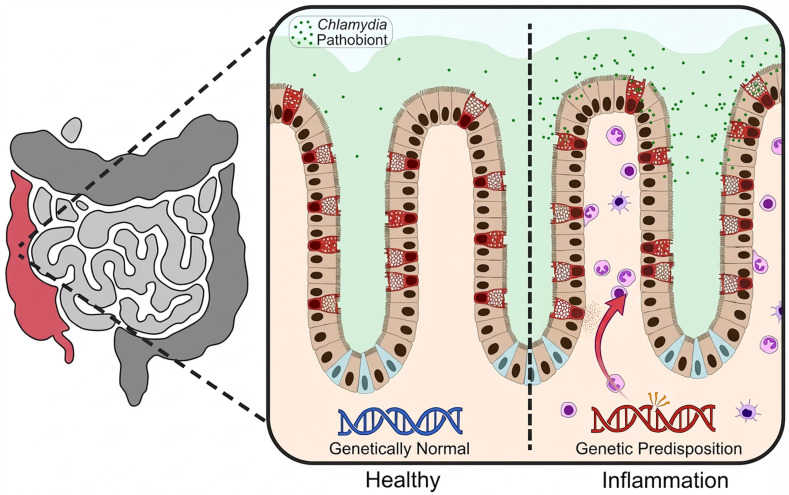

## Introduction

Organ regionalization plays important roles in organ function and in shaping disease patterns. High-resolution studies reveal that each intestinal segment exhibits distinct epithelial features, immune defenses, and responses to local microenvironmental cues (1, 2). The ileum is specialized for nutrient absorption, including vitamin B12 and bile salts, and limits microbial exposure through Paneth cell–derived antimicrobials (3). In contrast, the colon harbors a substantially higher microbial burden and is primarily dedicated to water, electrolyte, and short-chain fatty acid absorption. Moreover, the ileum, proximal colon, and distal colon differ in their epithelial structure and mucus composition (4–8). These regional specializations are likely implicated in the pathogenesis of inflammatory bowel disease (IBD), including Crohn’s disease (CD) (9–15). Although CD can involve any part of the gastrointestinal (GI) tract, inflammation most commonly localizes to the terminal ileum (TI) and/or ascending (proximal) colon (AC) (16–20). The factors driving disease emergence at these distinct sites remain unclear, and modern biologic and small molecule therapies that largely target inflammatory pathways are not tailored toward regional involvement, particularly within different colonic segments.

Manifestation of CD in the gut arises from a complex interplay among host genetics, immune responses, environmental factors, and the microbiome. Genome-wide association studies have identified over 240 IBD susceptibility loci, implicating immune pathways (10, 12, 21) and epithelial barrier regulation (22, 23), yet host genetics alone are insufficient to drive disease outside of rare monogenic cases (24). Animal models further highlight the essential role of the microbiome, with disease emerging in genetically susceptible hosts following colonization by specific disease-promoting microbes, often termed pathobionts (25–28). Pathobionts typically do not confer symptoms but can occupy distinct intestinal niches and provoke inflammation in a context-dependent manner. *Chlamydia* species exemplify such a niche-adapted pathobiont (29). Although best known as urogenital pathogens, *Chlamydia* species can colonize multiple mucosal tissues, including the intestine (30), where certain serovars cause proctocolitis (known as lymphogranuloma venereum) that clinically resembles IBD (31, 32). As obligate intracellular bacteria, *Chlamydia* primarily replicate within epithelial cells, employing various immune evasion mechanisms (33–35). Host responses, including induction of indoleamine 2,3-dioxygenase 1 (IDO1), restrict *Chlamydia* growth by depleting tryptophan, as many species are tryptophan auxotrophs (36–38). While potentially beneficial for the clearance of intracellular microbes, IDO1 induction also promotes *Chlamydia* persistence (39) and has broad impacts on the microenvironment, such as the specification of intestinal secretory cells (40) and the activation of Tregs (41–44). IDO1 is upregulated in inflamed intestinal mucosa, including in IBD (45–47), yet its mechanistic links to intracellular pathobionts and region-specific disease remain poorly defined.

Here, we demonstrate that in a CD-like, immune-dysregulated host, *Chlamydia muridarum* acts as a pathobiont, inducing an aberrant inflammatory program originating from IDO1-expressing goblet cells in the proximal colon (PC). Consistently, human CD with AC involvement shows epithelial IDO1 upregulation in inflammation-associated epithelial cell states, a signature absent in TI disease, revealing a regionally encoded inflammatory program driven by the interplay of host susceptibility, pathobiont colonization, and locally specialized host epithelial responses.

## Results

### The microbiome drives inflammation in the PC of a genetically susceptible host with dysregulated TNF expression.

**Figure 1:**
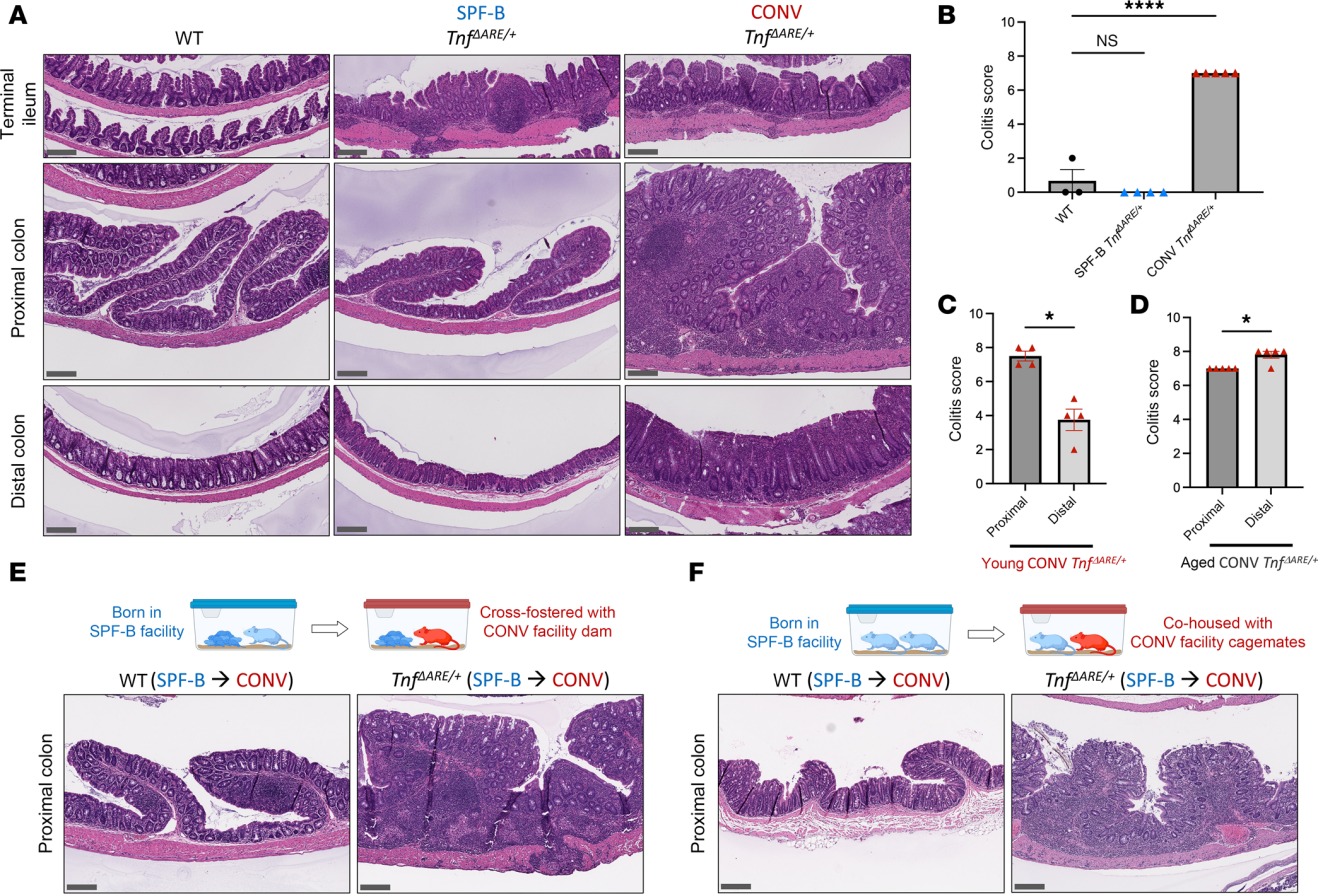
Crohn’s-like disease in the PC is associated with murine housing facility. (**A**) Representative H&E-stained intestinal sections from WT (*N* = 3) and *Tnf^ΔARE/+^* mice from SPF-B (*N* = 4) and CONV facilities (*N* = 5). WT samples are from the CONV facility, and all mice are age-matched (34–42 weeks). (**B**) Colitis scores from histopathological analysis of murine colons from **A**. (**C** and **D**) Colitis scores by colonic regions of CONV *Tnf^ΔARE/+^* mice (*N* = 4 for 12 weeks in **C**; *N* = 5 for 34–42 weeks in **D**). (**E**) Representative H&E-stained PC sections from aged (37 weeks) SPF-B mice (*N* = 3 WT, *N* = 4 *Tnf^ΔARE/+^*) transferred and cohoused/fostered as pups in the CONV facility with a WT or *Tnf^ΔARE/+^* foster dam. (**F**) Representative H&E-stained PC sections from adult (32–54 weeks) SPF-B mice (*N* = 2 WT, *N* = 3 *Tnf^ΔARE/+^*) transferred and cohoused in the CONV facility in mixed-sex conditions until experimental collection. All scale bars: 200 μm. Data are shown as the mean ± SEM in quantifications. Statistical significance was determined using an ordinary 1-way ANOVA with Dunnett’s multiple-comparison test with CONV WT as the control group (**B**) or using paired 2-tailed *t* tests (**C** and **D**). *P < 0.05, ****P < 0.0001. See also Supplemental Figure 1.

While TNF is a critical cytokine for both ileal and colonic CD pathogenesis, how some patients develop inflammatory disease only in the TI, PC/AC, or both is unclear (48–50). We hypothesized that exogenous factors, such as external stressors and specific microbes, influence the regional specificity of disease manifestation in a TNF-dependent manner. To investigate this, we used the *Tnf*^ΔARE/+^ mouse, an established, TNF-driven model of CD that develops inflammation in the TI with only rare and mild inflammation reported in the colon (28, 51). To determine if exogenous factors drive TNF-dependent inflammation in a region-specific manner, we reared mice in specific pathogen–free barrier (SPF-B) and conventional (CONV) facilities, which differ in their animal management policies. *Tnf*^ΔARE/+^ mice developed the expected terminal ileitis phenotype in SPF-B and CONV facilities (Figure 1A and Supplemental Figure 1A; supplemental material available online with this article; https://doi.org/10.1172/JCI201729DS1). Surprisingly, *Tnf*^ΔARE/+^ mice reared in the CONV housing facility, but not those in SPF-B, developed severe colitis that was most pronounced in the PC (Figure 1, A and B, and Supplemental Figure 1, B and C). Colonic inflammation was 100% penetrant in *Tnf*^ΔARE/+^ mice in the CONV facility and was established as early as 6 weeks of age (Supplemental Figure 1D). Colonic inflammation initially developed in the PC of young mice with corresponding increases in TNF protein levels in this region, and it spread to the distal colon at later stages of disease in aged mice (Figure 1, C and D, and Supplemental Figure 1, C and E). The most prevalent features of colonic inflammation were an expansion of the lamina propria compartment and depth of inflammation extending into the submucosa and muscularis propria (Supplemental Figure 1F). Of note, no cases of colonic inflammation were observed in *Tnf*^ΔARE/+^ mice from the SPF-B facility nor in any of the WT mice in either facility, even in mice aged up to 1 year (Supplemental Figure 1G). Our findings indicate that exogenous factors can influence the site of inflammation within the gut of a genetically susceptible host, reminiscent of human CD where the TI and PC/AC are the 2 most affected sites.

Given the multifactorial nature of CD (15, 52), we investigated various potential drivers of proximal colitis. We excluded the effects of genetic drift, as CONV mice were extensively backcrossed to C57BL/6J breeders from The Jackson Laboratory and validated by SNP-based background testing to be inbred comparable to SPF-B mice (Supplemental Figure 1H). To assess nonmicrobial environmental effects in the CONV facility, such as caging and food supply, colitis-free WT and *Tnf*^ΔARE/+^ mice from the SPF-B facility were transferred to the CONV facility without cohousing, where neither genotype developed colitis (Supplemental Figure 1I). We then examined the role of the microbiota by cohousing colitis-free, SPF-B WT and *Tnf*^ΔARE/+^ mice with WT or *Tnf*^ΔARE/+^ mice from the CONV facility. SPF-B *Tnf*^ΔARE/+^ mice that were transferred and cohoused developed colitis in the PC, while transferred and cohoused SPF-B WT mice were free of colonic inflammation (Figure 1, E and F, and Supplemental Figure 1, J and K). Of note, *Tnf*^ΔARE/+^ mice developed PC inflammation under multiple experimental conditions, including transfer as pups to a foster dam (Figure 1E and Supplemental Figure 1J) or transfer as adults (Figure 1F and Supplemental Figure 1K), suggesting that the development of colonic inflammation is independent of the developmental stage of both the host’s immune system and microbiota. We demonstrated that the CONV microbiota, and not a specifically TNF-driven microbiota, is sufficient to confer inflammation of the PC, as WT foster dams were able to transfer colitis to SPF-B *Tnf*^ΔARE/+^ pups, as well as WT adults to SPF-B *Tnf*^ΔARE/+^ adults. In sum, proximal colitis in *Tnf*^ΔARE/+^ mice is microbiota driven and independent of background genetics, nonmicrobial environment, and developmental stage.

### Identification of C. muridarum as a pathobiont in TNF-associated inflammation in the PC.

**Figure 2:**
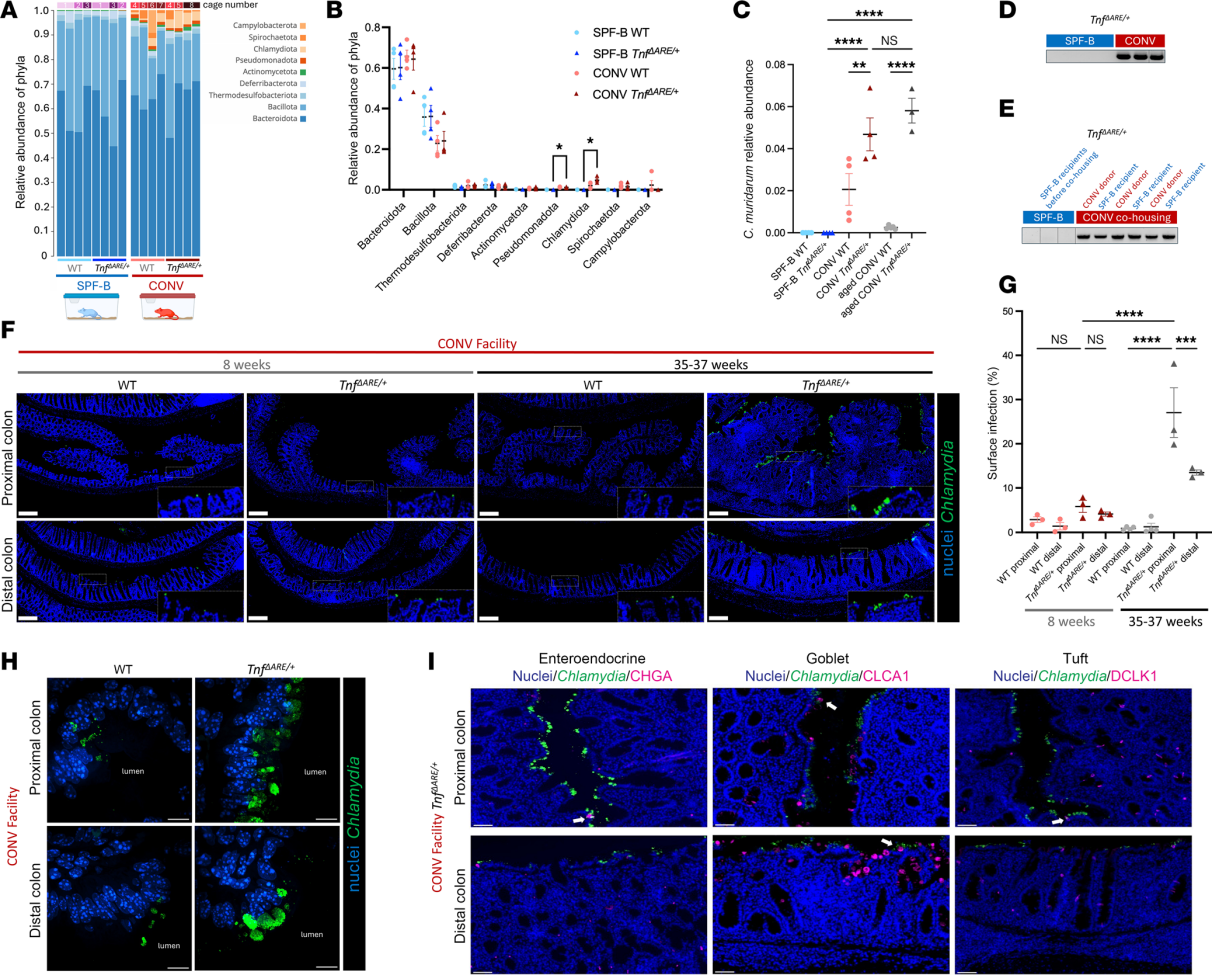
*C*. *muridarum* is associated with PC inflammation in the context of TNF overexpression. (**A** and **B**) Eubacterial shotgun metagenomic data of murine PC luminal contents represented as relative abundance of mapped phyla for individual mice across 8 cages (*N* = 4 per condition). Statistical significance was determined using multiple unpaired 2-tailed *t* tests with FDR of 1%. *P < 0.05. (**C**) Data from **A** mapped to *C*. *muridarum* species. Statistical significance was determined using an ordinary 1-way ANOVA with Šidák’s multiple-comparison test. **P < 0.01, ****P < 0.0001. (**D**) Fecal DNA-based PCR testing for *C*. *muridarum* in *Tnf^ΔARE/+^* mice from SPF-B (*N* = 4) and CONV (*N* = 3) facilities. (**E**) Fecal DNA-based PCR testing for *C*. *muridarum* in SPF-B *Tnf^ΔARE/+^* mice before and after cohousing with *Tnf^ΔARE/+^* mice from the CONV facility. *N* = 3 independent experiments. (**F**) Representative IF images, with insets, of *Chlamydia* major outer membrane protein (MOMP) and nuclei costaining on colonic sections from WT and *Tnf^ΔARE/+^* mice from the CONV facility (*N* = 3 each). Scale bars: 200 μm. Original magnification, ×20. (**G**) Quantification of *Chlamydia* from IF images. Statistical significance was determined using an ordinary 1-way ANOVA with Šidák’s multiple-comparison test. ***P < 0.001, ****P < 0.0001. (**H**) Representative confocal high-magnification IF stained images of *Chlamydia* MOMP and nuclei costaining. *N* = 3 per condition. Scale bars: 10 μm. (**I**) Representative IF images of *Chlamydia* MOMP and nuclei costaining with epithelial cell type–specific markers on colonic sections from age-matched CONV facility *Tnf^ΔARE/+^* mice (*N* = 5, 35–37 weeks of age). White arrows point to MOMP^+^ enteroendocrine, goblet, or tuft cells. Scale bars: 50 μm. Data are shown as the mean ± SEM in quantifications. See also Supplemental Figure 2, Supplemental Unedited blot and gel images, and Supplemental Table 1.

To identify pathobiont species that drive colonic inflammation, we performed shotgun metagenomics of PC luminal contents to identify microbes associated with the most inflamed region (Supplemental Table 1). Overall, the α diversity, at the species level, was not significantly different between genotypes and across facilities and ages, suggesting that within-sample microbial diversity is similar across conditions (Supplemental Figure 2A). In young mice, there were no major phylum-level differences between WT and *Tnf*^ΔARE/+^ microbiota within each facility, suggesting TNF overexpression does not shift phylum-level composition (Figure 2, A and B). However, in the CONV facility, differences in phylum-level composition emerged between WT and *Tnf*^ΔARE/+^ microbiota in aged conditions, suggesting TNF overexpression influences the composition of the PC microbiota at later stages of disease (Supplemental Figure 2, B and C). Moreover, differences between WT and *Tnf*^ΔARE/+^ β diversity became more pronounced in aged conditions (Supplemental Figure 2D).

However, when comparing young *Tnf*^ΔARE/+^ mice across facilities, we found Chlamydiota and Pseudomonadota phyla were enriched in the PCs of CONV mice compared with SPF-B mice (Figure 2, A and B). Reads for the Chlamydiota phyla mapped to a single species, *C*. *muridarum*, an obligate intracellular bacterium and the only natural *Chlamydia* pathogen of mice. *C*. *muridarum* is commonly used as a model to study human infections from *Chlamydia trachomatis*, a sexually transmitted disease–causing bacterium that is also associated with colonic inflammation (30, 31). While *C*. *muridarum* was undetected in the PC microbiota of SPF-B mice of either genotype, the relative abundance of *C*. *muridarum* was higher in the PC microbiota of CONV *Tnf*^ΔARE/+^ mice compared with CONV WT mice, with more pronounced differences in aged conditions (Figure 2C). Moreover, the relative abundance of *C*. *muridarum* in the PC of *Tnf*^ΔARE/+^ mice was not significantly different in young versus aged mice. These results suggest that WT mice can mitigate *C*. *muridarum* colonization over time, but *Tnf*^ΔARE/+^ mice cannot control its growth. PCR-based fecal testing for *Chlamydia* confirmed all tested SPF-B *Tnf*^ΔARE/+^ mice were *Chlamydia* negative, while all CONV *Tnf*^ΔARE/+^ mice were *Chlamydia* positive (Figure 2D and Supplemental Unedited blot and gel images). We then analyzed fecal samples of mice in transfer and cohousing experiments. We found SPF-B *Tnf*^ΔARE/+^ mice were *Chlamydia* positive upon cohousing, indicating that *Chlamydia* is a transmissible component of the microbiota that is associated with PC inflammation in *Tnf*^ΔARE/+^ mice (Figure 2E and Supplemental Unedited blot and gel images). Given the role of *Chlamydia* species in human sexually transmitted disease, we asked whether sexual transmission is required to establish GI colonization. We cohoused *Tnf*^ΔARE/+^ mice in same-sex conditions and found that *Tnf*^ΔARE/+^ mice developed PC inflammation, suggesting against a strictly sexually transmitted route and supporting a fecal-oral route (Supplemental Figure 2E) (53, 54). Overall, these findings identify *C*. *muridarum* as a potential pathobiont associated with inflammation in the PC of *Tnf*^ΔARE/+^ mice.

Next, we used immunofluorescence (IF) microscopy to assess the site of *C*. *muridarum* colonization along the length of the GI tract of WT and *Tnf*^ΔARE/+^ mice. Intracellular inclusions of *Chlamydia* were detected at high levels in the PC of *Tnf*^ΔARE/+^ mice and progressively decreased in a gradient-like manner toward the distal colon, which correlated to the degree of inflammation (Figure 2, F and G, and Supplemental Figure 2F). Consistent with shotgun metagenomics data, *C*. *muridarum* was not detected by IF microscopy in the PC of SPF-B mice (Supplemental Figure 2G). The small intestine was largely devoid of *Chlamydia* inclusions in mice from either facility or genotype, with only rare ileal inclusions detected in *Tnf*^ΔARE/+^ mice (Supplemental Figure 2, H and I). Shotgun metagenomic sequencing of ileal luminal contents from WT and *Tnf*^ΔARE/+^ mice revealed the presence of *C*. *muridarum* in all samples, though relative abundance varied widely, with some samples dominated by *C*. *muridarum* (Supplemental Figure 2J). Given our observations in IF imaging, the variability and high apparent abundance of *C*. *muridarum* in the ileum more likely reflect the relatively low microbial load of this region (55), where even limited presence can appear dominant in sequencing data due to the paucity of other microbes, rather than true colonization. Collectively, these findings suggest that while *C*. *muridarum* is capable of colonizing the ileal compartment, it is not the preferred niche nor is it required for terminal ileitis. Instead, *C*. *muridarum* preferentially colonizes the colon and is associated with inflammation of the PC.

In addition to IF-based detection of *Chlamydia*, we used RNA-FISH to detect *C*. *muridarum* 23S ribosomal RNA and found a similar pattern of colonization in CONV WT and *Tnf*^ΔARE/+^ mice (Supplemental Figure 2K). *Chlamydia*-positive cells were restricted to the luminal surface epithelium, with high-resolution imaging showing apically localized intracellular *C*. *muridarum* inclusions adjacent to the nucleus that were larger in *Tnf*^ΔARE/+^ than in WT mice (Figure 2H and Supplemental Figure 2L). We then determined the cellular tropism of *C*. *muridarum* by examining its colocalization with colonic epithelial cell markers. At the luminal surface, *Chlamydia*-positive epithelial cells are abundant, with only rare colocalization with secretory lineage cells (enteroendocrine, goblet, and tuft cells) (Figure 2I). Given the predominance of absorptive cells in the colonic epithelium, our results suggest that *Chlamydia* can infect both secretory and absorptive cells in the PC but exhibits tropism for luminal surface absorptive epithelial cells.

### C. muridarum is necessary and sufficient to drive TNF-dependent PC inflammation.

**Figure 3:**
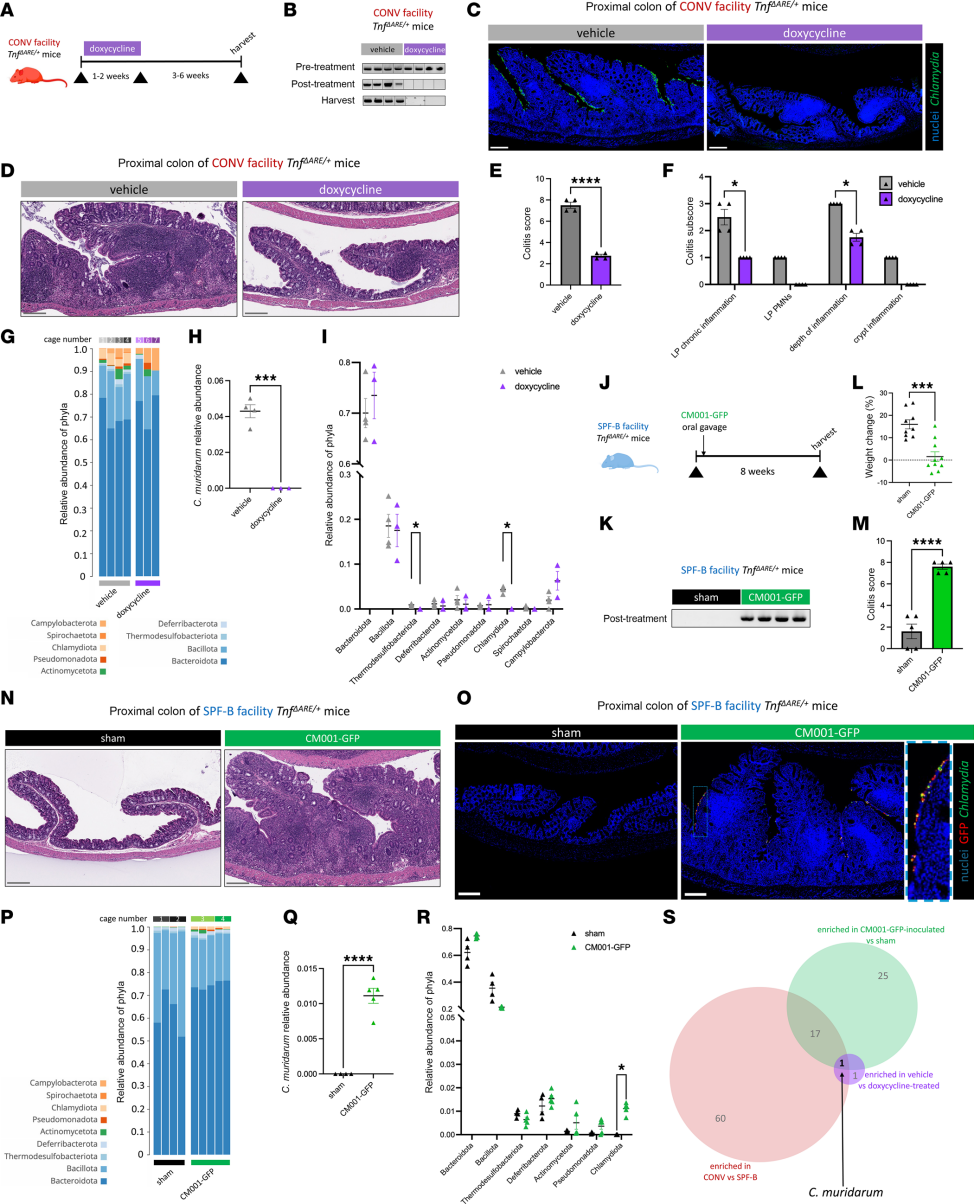
*C*. *muridarum* is necessary and sufficient to drive PC inflammation in the *Tnf^ΔARE/+^* model. (**A**) Doxycycline experiment scheme for **B**–**I**, age-matched at 11–12 weeks at harvest. *N* = 4 mice per condition for **B**–**F**. (**B**) Fecal testing for *C*. *muridarum* at different time points. (**C** and **D**) Representative IF of *Chlamydia* MOMP and nuclei (**C**) and H&E-stained PC sections (**D**) from the doxycycline experiment. (**E**) Colitis scores of samples from **D**. (**F**) Colitis subscores that comprise **E**. (**G**) Eubacterial shotgun metagenomic data of PC luminal contents of mice treated with doxycycline (*N* = 4) or vehicle (*N* = 3), represented as relative abundance of mapped phyla for individual *Tnf^ΔARE/+^* mice of mixed ages (11–23 weeks of age) across 7 cages. (**H**) Data from **G** with reads specifically mapped to *C*. *muridarum* species. (**I**) Data from **G** plotted. (**J**) CM001-GFP experimental scheme for **K**–**R**, age-matched at 16–20 weeks at tissue harvest. (**K**) Fecal testing for *C*. *muridarum* in *Tnf^ΔARE/+^* mice from the CM001-GFP experiment. *N* = 4 mice per condition. (**L**) Body weight change from the CM001-GFP experiment. *N* = 10 mice per condition. (**M**) Colitis scores from the CM001-GFP experiment. *N* = 5 each condition. (**N**) H&E-stained PC sections from the CM001-GFP experiment. *N* = 10 mice per condition. (**O**) Representative IF images with insets of MOMP and endogenous GFP from CM001-GFP on PC sections. *N* = 5 mice per condition. Original magnification, ×20. (**P**) Eubacterial shotgun metagenomic data of PC luminal contents from *Tnf^ΔARE/+^* mice in CM001-GFP-inoculated (*N* = 5) or sham (*N* = 4) conditions across 4 cages. (**Q** and **R**) Same analyses as in **H** and **I** but with data from **P**. (**S**) Shared enriched species from eubacterial shotgun metagenomic data analyses in Supplemental Figure 4, G–J, L, and M. All scale bars: 200 μm. Data are shown as the mean ± SEM in quantifications. Statistical significance was determined using unpaired 2-tailed *t* tests (**E**, **H**, **L**, **M**, and **Q**) or multiple unpaired 2-tailed *t* tests with FDR of 1% (**F**, **I**, and **R**). *P < 0.05, ***P < 0.001, ****P < 0.0001. See also Supplemental Figures 3 and 4, Supplemental Tables 2 and 3, and Supplemental Unedited blot and gel images.

Given our finding of an association of *C*. *muridarum* with inflammation of the PC, along with the established role of specific *C*. *trachomatis* serovars in human colon pathology (31, 32) and the role of TNF signaling in *Chlamydia*-induced pathology (56, 57), we assessed the necessity and sufficiency of *C*. *muridarum* in inducing colonic inflammation in *Tnf*^ΔARE/+^ mice. To determine necessity, we treated CONV WT and *Tnf*^ΔARE/+^ mice with doxycycline, a clinically effective antibiotic for clearing *Chlamydia* infections in humans (Figure 3A) (58). *Chlamydia* was not detected by PCR in fecal samples from weanling-aged WT and *Tnf*^ΔARE/+^ mice treated with doxycycline for 1–2 weeks and remained undetectable at the time of harvest (Figure 3, B and C, Supplemental Figure 3A, and Supplemental Unedited blot and gel images). In mice and humans, the native gut microbiota gradually recovers after cessation of antibiotics (59, 60). Thus, we evaluated the extent of inflammation in the PC 3–6 weeks after antibiotic withdrawal. Strikingly, doxycycline-treated *Tnf*^ΔARE/+^ mice had significantly lower PC inflammation compared with vehicle control–treated, age-matched *Tnf*^ΔARE/+^ mice (Figure 3, D–F, and Supplemental Figure 3B). Unlike inflammation in the PC, doxycycline did not reduce ileal inflammation in *Tnf*^ΔARE/+^ mice compared with vehicle control conditions (Supplemental Figure 3C). This, combined with our findings that *C*. *muridarum* does not readily colonize the small intestine (Supplemental Figure 2, H–J), further suggests that *C*. *muridarum* presence does not promote nor protect against ileal inflammation. At the time of harvest, *C*. *muridarum* remained undetectable in shotgun metagenomic data of luminal PC contents of doxycycline-treated *Tnf*^ΔARE/+^ mice but was abundant in the vehicle control group (Figure 3, G and H, and Supplemental Table 2). Because doxycycline can alter microbial community structure, we assessed whether the recovered microbiome was broadly changed in treated mice and found that, at the phylum level, only Chlamydiota and Thermodesulfobacteriota were significantly reduced (Figure 3I). We ruled out the role of Thermodesulfobacteriota in PC inflammation, as these microbes were also present in SPF-B mice without colonic inflammation (Figure 2, A and B, and Supplemental Table 1). In aged mice with established inflammation prior to doxycycline administration, there was no overall reduction of inflammation upon treatment with doxycycline compared with vehicle-treated controls, which is unsurprising given that reversal of advanced disease is likely more complex (Supplemental Figure 3, D–F). However, analysis of colitis subscores revealed a statistically significant reduction in lamina propria chronic inflammation, indicating that partial reversal of inflammation was attained with doxycycline treatment (Supplemental Figure 3E). These results indicate that *C*. *muridarum* is required for inflammation in the PC without affecting ileitis, in a manner largely independent of other bacteria.

To determine whether *C*. *muridarum* is sufficient to induce inflammation in the PC, we inoculated *Chlamydia-*free *Tnf*^ΔARE/+^ mice from the SPF-B facility with a typed *C*. *muridarum* Nigg strain expressing GFP, CM001-GFP (61), which shares approximately 99% sequence similarity (ANIb = 0.988, TETRA = 0.999) to the *C*. *muridarum* strain we isolated from our CONV facility (*C*. *muridarum* strain VU [*Cm*-VU]) (Figure 3J and Supplemental Figure 4, A and B). CM001-GFP successfully engrafted into the microbiome of SPF-B *Tnf*^ΔARE/+^ mice after a single gavage without the need for antibiotic pretreatment (Figure 3K and Supplemental Unedited blot and gel images). CM001-GFP–inoculated *Tnf*^ΔARE/+^ mice were not overtly ill compared with the sham-inoculated *Tnf*^ΔARE/+^ mice; however, they exhibited significantly lower weight gain compared with sham-inoculated *Tnf*^ΔARE/+^ controls, consistent with GI inflammation (Figure 3L). CM001-GFP–inoculated mice remained *Chlamydia* positive at the time of harvest, and macroscopic and histological assessment revealed that CM001-GFP–inoculated SPF-B *Tnf*^ΔARE/+^ mice developed severe colonic inflammation reminiscent of the disease in the CONV facility, while sham-inoculated *Tnf*^ΔARE/+^ mice showed no colonic inflammation, similar to untreated SPF-B mice (Figure 3, M–O, and Supplemental Figure 4, C–E). Inoculation with CM001-GFP had no effect on the extent of ileal inflammation in *Tnf*^ΔARE/+^ mice, consistent with region-specific susceptibility (Supplemental Figure 4F). To determine whether *C*. *muridarum* colonization indirectly drives additional community-level microbial changes to induce PC inflammation, we performed shotgun metagenomic sequencing of the PC luminal contents. Compared with sham-inoculated mice, CM001-GFP–inoculated mice had no significant changes at the phylum level, except for Chlamydiota (Figure 3, P–R, and Supplemental Table 3), demonstrating CM001-GFP engraftment does not induce global shifts in the microbiome.

To test whether *C*. *muridarum* is necessary and sufficient to drive colonic inflammation, we analyzed species-level shotgun metagenomic data across all experimental conditions (Supplemental Figure 4G and Supplemental Tables 1–3). Species enrichment was not driven by age, as no species differed between young and aged CONV *Tnf*^ΔARE/+^ PC samples, and comparisons of young SPF-B *Tnf*^ΔARE/+^ mice with either young or aged CONV *Tnf*^ΔARE/+^ mice yielded similar numbers of enriched species (78 and 61, respectively) with substantial overlap (Supplemental Figure 4, H–K). Having excluded age as a confounder, we identified candidate pathobionts by intersecting species enriched across 3 comparisons: CONV versus SPF-B, vehicle-treated versus doxycycline-treated, and CM001-GFP–inoculated versus sham-inoculated *Tnf*^ΔARE/+^ mice (Supplemental Figure 4, J, L, and M). *C*. *muridarum* was the only species common to all conditions (Figure 3S). Taken together, these results strongly support that *C*. *muridarum* is necessary and sufficient to drive PC inflammation in a genetically susceptible host, such as in the *Tnf*^ΔARE/+^ model.

### Chlamydia colonization induces IDO1 expression in goblet cells.

**Figure 4:**
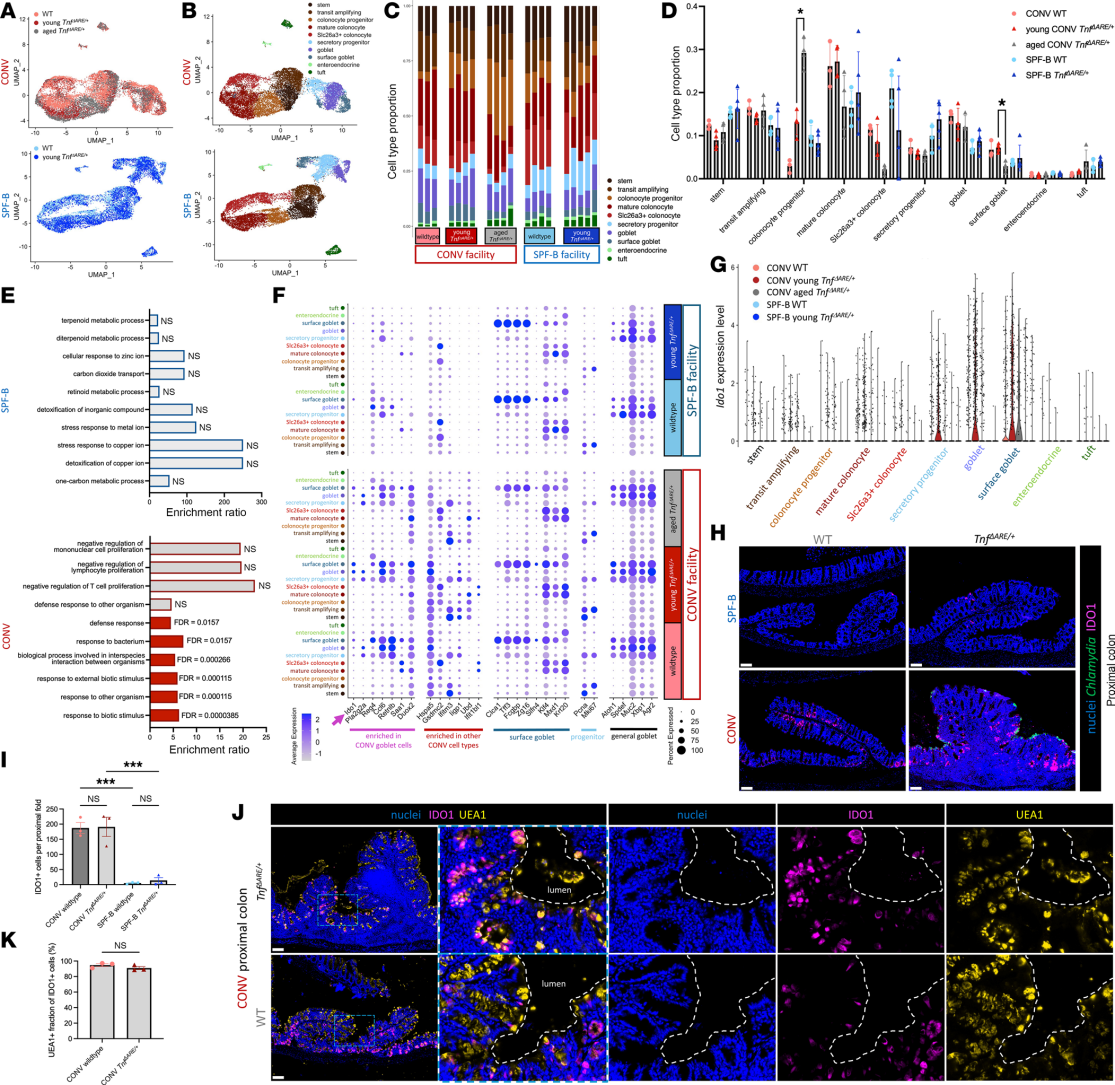
*C*. *muridarum* colonization induces IDO1 expression in PC goblet cells. (**A** and **B**) UMAP coembedding of scRNA-seq data from PC epithelial cells from CONV WT (*N* = 3), CONV young *Tnf^ΔARE/+^* (*N* = 4), CONV aged *Tnf^ΔARE/+^* (*N* = 4), SPF-B WT (*N* = 4), and SPF-B young *Tnf^ΔARE/+^* (*N* = 5) mice with sample type overlay (**A**) and cell type (**B**) overlay. (**C** and **D**) Cell type proportions from scRNA-seq data in **B** grouped by sample type. Statistical significance was determined using multiple unpaired 2-tailed *t* tests between 3 pairwise comparisons with FDR of 1%. Only statistically significant results are shown. *P < 0.05. (**E**) Gene Ontology of Biological Process terms derived from ORA of upregulated genes in cells from the SPF-B facility (top) or CONV facility (bottom). Enrichment ratio is shown, and statistical significance was determined using FDR < 0.05. (**F**) Cell type–specific expression of genes selected from those reaching statistical enrichment in ORA from **E** and from select marker genes for surface goblet cells, progenitors, and goblet cells. (**G**) Cell type–specific expression of *Ido1* from data in **A** and **B**. (**H**) Representative IF images of IDO1, *Chlamydia* MOMP, and nuclei costaining on PC sections from age-matched (34–42 weeks) WT and *Tnf^ΔARE/+^* mice from the SPF-B and CONV facilities. *N* = 3 mice per condition. Data in top right image first appear as nuclei/MOMP costaining (no IDO1) in Supplemental Figure 2G. (**I**) Quantitative analysis of images in **H**. Statistical significance was determined using an ordinary 1-way ANOVA with Šidák’s multiple-comparison test. ***P < 0.001. (**J**) Representative IF images, with insets, of IDO1, UEA1 lectin, and nuclei costaining on PC sections from age-matched (16–17 weeks) WT and *Tnf^ΔARE/+^* mice from the CONV facility. Dashed white lines indicate border between epithelial surface and lumen. *N* = 3 mice per condition. Original magnification, ×20. (**K**) Quantitative analysis of images in **J**. Statistical significance was determined using an unpaired 2-tailed *t* test. All scale bars: 100 μm. Data are shown as the mean ± SEM in quantifications. See also Supplemental Figures 5 and 6, and Supplemental Tables 4–7.

We sought to identify host responses to *C*. *muridarum* colonization with a focus on epithelial cells of the PC, as these cells are exclusive targets of *C*. *muridarum* tropism. We performed scRNA-seq on the PC epithelia of WT and *Tnf*^ΔARE/+^ mice from SPF-B and CONV facilities, including young and aged *Tnf*^ΔARE/+^ mice from the CONV facility (Figure 4A and Supplemental Figure 5A). Within each housing facility, cells of WT and *Tnf*^ΔARE/+^ PC were intermixed in the UMAP coembedding, suggesting that neither TNF overexpression nor *C*. *muridarum* abundance drastically alters colonic epithelial cells. While major transcriptomic differences amongst cell types were not observed, we aimed to determine whether epithelial cell type proportions were different amongst conditions (Figure 4, B and C, Supplemental Figure 5B, and Supplemental Table 4). Comparison of young CONV *Tnf*^ΔARE/+^ samples with CONV WT, aged CONV *Tnf*^ΔARE/+^, and young SPF-B *Tnf*^ΔARE/+^ samples revealed only 2 statistically significant differences: a decrease in surface goblet cells and an increase in colonocyte progenitors in aged CONV *Tnf*^ΔARE/+^ PC compared with young CONV *Tnf*^ΔARE/+^ PC (Figure 4D). Strikingly, there were no differences amongst epithelial cells between young CONV *Tnf*^ΔARE/+^ and young SPF-B *Tnf*^ΔARE/+^. These findings suggest that *C*. *muridarum* colonization does not induce changes in host responses by promoting the depletion or expansion of particular epithelial cell types of the PC, nor by drastically altering cell states, with the exception of a loss of surface goblet cells and expansion of colonocyte progenitors in later stages of inflammation.

Next, we aimed to identify cell type–specific changes in gene expression in *Chlamydia-*colonized (CONV facility) versus uncolonized (SPF-B facility) conditions. Surprisingly, our analysis revealed only a limited number of differentially expressed genes when comparing individual PC epithelial cell types between CONV and SPF-B housed mice (Supplemental Figure 5C and Supplemental Table 5). We performed a more direct comparison between young *Tnf*^ΔARE/+^ samples in CONV conditions and young *Tnf*^ΔARE/+^ samples in SPF-B conditions, which yielded a similar set of differentially expressed genes to those in the broader comparison, most of which were shared between comparisons (Supplemental Figure 5C and Supplemental Table 6). These results support that *C*. *muridarum* colonization induces modest transcriptomic changes in WT and *Tnf*^ΔARE/+^ PC epithelial cell types.

To reveal specific gene program alterations, we performed gene overrepresentation analysis (ORA) using statistically upregulated genes from all cell types in the CONV or SPF-B facilities, separately, as input (Figure 4E and Supplemental Tables 6 and 7). Terms related to colonic cell function were enriched in SPF-B specimens but did not reach statistical significance. In contrast, several terms were statistically enriched in CONV specimens, all of which were terms related to host responses to microbes. These results indicate a loss of normal function and induction of defense responses in the PC epithelial cells from *Chlamydia-*positive mice in the CONV facility. We examined genes that reached statistical enrichment in ORA and found that many were enriched in goblet cell subtypes and secretory progenitors, with the most prominent and statistically significant expression differences in surface goblet cells (Supplemental Tables 6 and 7, and Figure 4F). Generally, genes contributing to these ORA terms fell into broad functional categories related to antimicrobial function or regulation of immune and inflammatory responses with a particular emphasis on IFN response genes, which have not been directly investigated in goblet cells. One such IFN response gene, *Ido1*, was expressed at high levels in a subset of goblet cells and surface goblet cells in the PC of mice from the CONV facility and at low levels in a small subset of goblet cells from the PC of mice from the SPF-B facility (Figure 4, F and G, and Supplemental Figure 6A). Within the CONV facility, *Ido1* was expressed at higher levels in goblet and surface goblet cells of *Tnf*^ΔARE/+^ mice compared with WT mice, which was intriguing given our observation of surface epithelial localization of *C*. *muridarum* in the colon. *Ido1* encodes the protein IDO1, an enzyme that converts tryptophan to kynurenine and has established roles in goblet cell differentiation (40), immunosuppression (41–44), and innate immune protection from infection (39), including from *Chlamydia* and other pathogens that require host-derived tryptophan (36–38). We examined IDO1 protein expression and found patterns consistent with our transcriptomic data; IDO1 was expressed at significantly higher levels in samples from the CONV facility compared with the SPF-B facility in both WT and *Tnf*^ΔARE/+^ PC, with signal most elevated in the PC compared with the distal colon and greater than 90% of IDO1-expressing cells being goblet cells (Figure 4, H–K, and Supplemental Figure 6, B and C). These results suggest a potential role for goblet cells in *Chlamydia*-induced PC inflammation via upregulation of IDO1.

### Chronic inflammation in the PC is potentiated through a goblet cell response–dependent mechanism.

**Figure 5:**
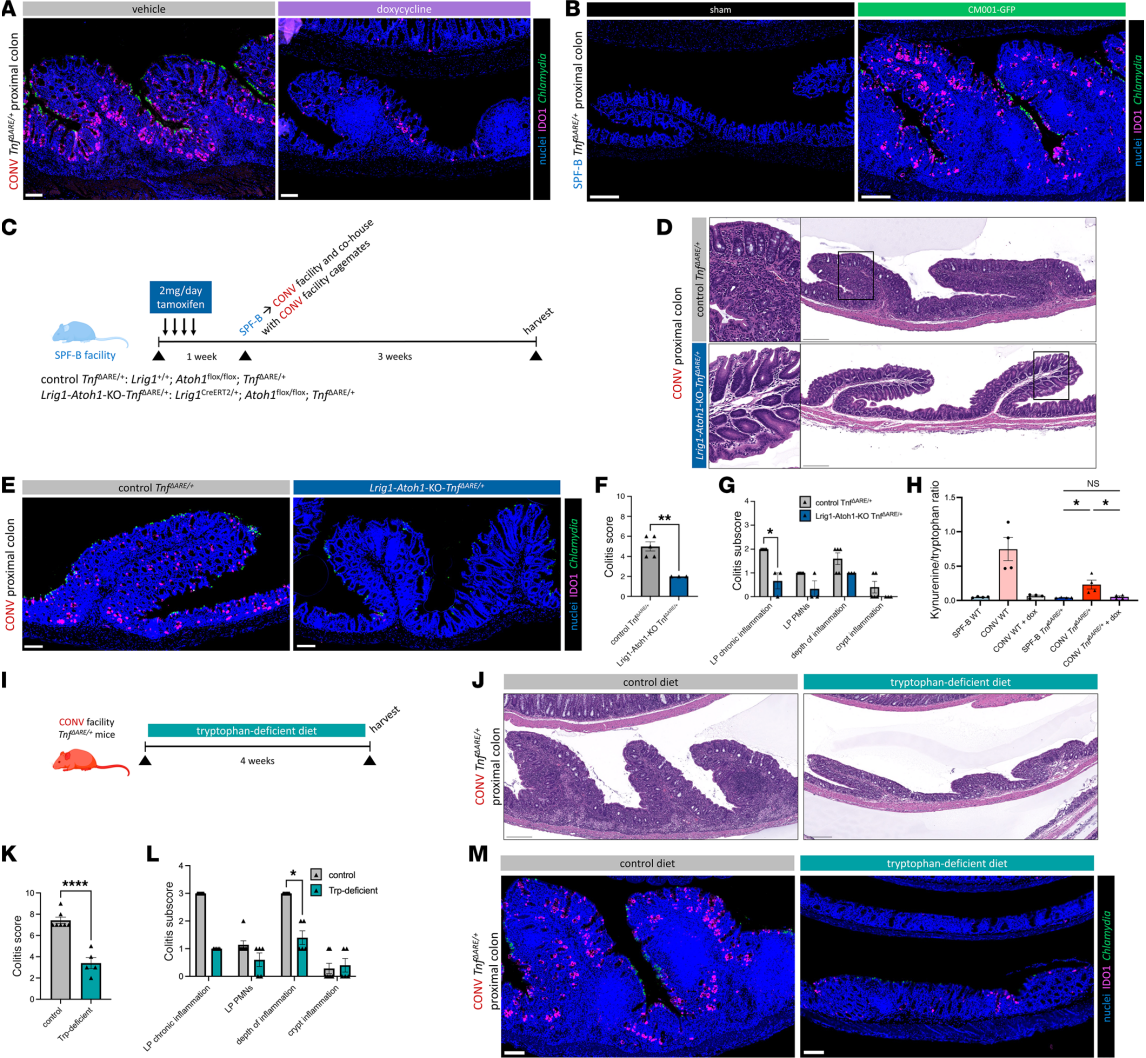
Perturbation of the IDO1 pathway reduces *Chlamydia*-driven PC inflammation in the *Tnf^ΔARE/+^* model. (**A**) Representative IF images of IDO1, *Chlamydia* MOMP, and nuclei costaining on PC sections from age-matched (11–12 weeks) CONV *Tnf^ΔARE/+^* mice treated with doxycycline or vehicle. *N* = 4 mice per condition. Scale bars: 100 μm. (**B**) Representative IF images as in **A** of PC sections from age-matched (16–20 weeks) SPF-B *Tnf^ΔARE/+^* mice that are sham or CM001-GFP inoculated. *N* = 5 mice per condition. Scale bars: 200 μm. (**C**) Experimental paradigm for secretory cell ablation in *Tnf^ΔARE/+^* mice. (**D**) Representative H&E-stained PC sections, with insets, from age-matched (20–27 weeks) *Tnf^ΔARE/+^* mice with (*N* = 3) or without (*N* = 5) secretory cell ablation. Scale bars: 200 μm. (**E**) Representative IF images as in **A** of PC sections from age-matched *Tnf^ΔARE/+^* mice in **D** with or without secretory cell ablation. Scale bars: 100 μm. (**F**) Colitis scores from histopathological scoring of colons from **D**. (**G**) Colitis subscores that contribute to overall colitis score in **F**. (**H**) Ratio of kynurenine to tryptophan, measured by liquid chromatography–mass spectrometry, in PC tissue from age-matched mice. *N* = 4 per condition. (**I**) Experimental paradigm for administration of tryptophan-deficient diet to *Tnf^ΔARE/+^* mice from the CONV facility. (**J**) Representative H&E-stained PC sections from age-matched (11–12 weeks) CONV *Tnf^ΔARE/+^* mice fed a control diet (*N* = 7) or a tryptophan-deficient diet (*N* = 5). Scale bars: 200 μm. (**K** and **L**) Histopathological scoring analysis as in **F** and **G** of colons from **J**. (**M**) Representative IF images as in **A** of PC sections from age-matched *Tnf^ΔARE/+^* mice in **J** fed a control or tryptophan-deficient diet. Scale bars: 100 μm. Data are shown as the mean ± SEM in quantifications. Statistical significance was determined using an unpaired 2-tailed *t* test (**F** and **K**), multiple unpaired 2-tailed *t* tests with FDR of 1% (**G** and **L**), or an ordinary 1-way ANOVA with Šidák’s multiple-comparison test (**H**). *P < 0.05, **P < 0.01, ****P < 0.0001. See also Supplemental Figures 7 and 8.

We next investigated whether IDO1 expression was dependent on the presence of *Chlamydia*. In CONV *Tnf*^ΔARE/+^ mice treated with doxycycline to clear *Chlamydia*, IDO1 expression was substantially lower in the PC compared with that in vehicle-treated *Tnf*^ΔARE/+^ mice (Figure 5A and Supplemental Figure 7A). Consistently, SPF-B *Tnf*^ΔARE/+^ mice inoculated with *C*. *muridarum* (CM001-GFP) showed increased IDO1 expression compared with sham-inoculated *Tnf*^ΔARE/+^ mice, on a similar level to CONV facility *Tnf*^ΔARE/+^ mice colonized by *Cm*-VU (Figure 5B and Supplemental Figure 7B). These results demonstrate that upregulation of IDO1 is induced by *C*. *muridarum* colonization of the PC.

We next investigated the relationship between IDO1 expression in goblet cells and *Chlamydia*-induced PC inflammation. Since IDO1 expression is restricted to goblet cells, we induced secretory cell ablation in *C*. *muridarum–*free SPF-B *Tnf*^ΔARE/+^ mice by knocking out the master secretory cell transcription factor *Atoh1* in intestinal and colonic stem cells (*Lrig1^CreERT2/+^ Atoh1^fl/fl^* [*Lrig1-Atoh1*-KO]) (62, 63). After Cre recombinase–mediated knockout of *Atoh1*, goblet cells within the PC of *Lrig1-Atoh1*-KO-*Tnf*^ΔARE/+^ mice were lost (Figure 5, C and D, and Supplemental Figure 7C). To colonize with *C*. *muridarum*, SPF-B *Lrig1-Atoh1*-KO-*Tnf*^ΔARE/+^ and *Tnf*^ΔARE/+^ control mice were cohoused with CONV facility cagemates naturally colonized with *Cm*-VU (Figure 5C). While *C*. *muridarum* was successfully transferred in all conditions, IDO1 expression was no longer detected in the PC epithelium of *Lrig1-Atoh1*-KO-*Tnf*^ΔARE/+^ mice (Figure 5E and Supplemental Figure 7D). Remarkably, PC inflammation in *Lrig1-Atoh1*-KO-*Tnf*^ΔARE/+^ mice was suppressed, while secretory cell–replete *Tnf*^ΔARE/+^ mice presented with severe PC inflammation (Figure 5, D, F, and G, and Supplemental Figure 7C). Given the localization of *C*. *muridarum* to the colonic surface and high *Ido1* expression in surface goblet cells, we repeated the same experiment where only the surface fraction of *Krt20^+^* goblet cells were depleted by Cre-mediated knockout of *Atoh1* (*Krt20^CreERT2/+^ Atoh1^fl/fl^* [*Krt20-Atoh1*-KO]). Indeed, only goblet cells at the crypt surface in the PC were depleted after tamoxifen induction in this model (Supplemental Figure 8A). Accordingly, IDO1 expression was also reduced at the crypt surface in the PC in *Krt20-Atoh1*-KO-*Tnf*^ΔARE/+^ mice (Supplemental Figure 8B). Like the *Lrig1-Atoh1*-KO*-Tnf*^ΔARE/+^ model where all goblet cells were depleted, surface goblet cell–depleted *Krt20-Atoh1*-KO-*Tnf*^ΔARE/+^ mice exhibited less severe PC inflammation characterized by less pronounced transmural inflammation (Supplemental Figure 8C). These results demonstrate the role of host responses in goblet cells, which is the main source of IDO1 expression, in inducing inflammation in the PC following *C*. *muridarum* colonization.

To assess whether goblet cell–associated IDO1 is active, we measured the levels of tryptophan, the substrate, and kynurenine, the product, in the PC of WT and *Tnf*^ΔARE/+^ mice in the CONV and SPF-B facilities. Both metabolites were elevated in the PC of CONV *Tnf*^ΔARE/+^ mice compared with the PC of SPF-B *Tnf*^ΔARE/+^ mice (Supplemental Figure 8D). While tryptophan levels can depend on various dietary, metabolic, and microbial factors, the ratio of kynurenine to tryptophan serves as a surrogate for enzymatic activity of IDO1 (64, 65). The kynurenine/tryptophan ratio was significantly elevated in the PC of CONV *Tnf*^ΔARE/+^ mice compared with SPF-B controls (Figure 5H). To determine whether IDO1 activity is directly driven by *C*. *muridarum* present in the CONV facility*,* we assessed the levels of these metabolites in the PC of doxycycline-treated CONV *Tnf*^ΔARE/+^ mice. Doxycycline treatment did not alter tryptophan levels but significantly reduced kynurenine levels and normalized the kynurenine/tryptophan ratio to *C*. *muridarum–*naive SPF-B levels (Figure 5H and Supplemental Figure 8D), indicating that elevated IDO1 activity in the PC is driven by *C*. *muridarum*, resulting in conversion of available tryptophan to downstream metabolites.

After determining that IDO1 is enzymatically active, we next sought to perturb this pathway to determine the role of IDO1 in PC inflammation in the *Tnf*^ΔARE/+^ mouse. We removed tryptophan from the diet of *Tnf*^ΔARE/+^ mice in the CONV facility to directly test if IDO1 enzymatic activity of converting tryptophan to kynurenine was responsible for driving PC inflammation (Figure 5I). PC inflammation of *Tnf*^ΔARE/+^ mice on a tryptophan-deficient diet was significantly suppressed compared with *Tnf*^ΔARE/+^ mice on a control diet (Figure 5, J–L, and Supplemental Figure 8E). Importantly, the PCs of *Tnf*^ΔARE/+^ mice on tryptophan-deficient diet remained colonized by *C*. *muridarum* and induction of IDO1 expression persisted, although both were reduced compared with the control diet condition (Figure 5M and Supplemental Figure 8F). The impact of the tryptophan-deficient diet is unlikely to occur by restricting the replication of *Cm-*VU because this strain, unlike other *C*. *muridarum* strains, encodes a functional *trpAB* operon that can use indole to bypass this nutritional restriction (EPS and RHV, unpublished observations). This ability to synthesize tryptophan from indole, an abundant metabolite that could be derived from the diet and microbiota, may allow *Cm-*VU to more effectively evade the host immune response (66, 67). These results further support our model that *Chlamydia-*induced aberrant goblet cell responses drive chronic inflammation in the PC of a genetically susceptible host.

### Chlamydia-induced colonic inflammation is independent of upstream ileal inflammation or antimicrobial function.

**Figure 6:**
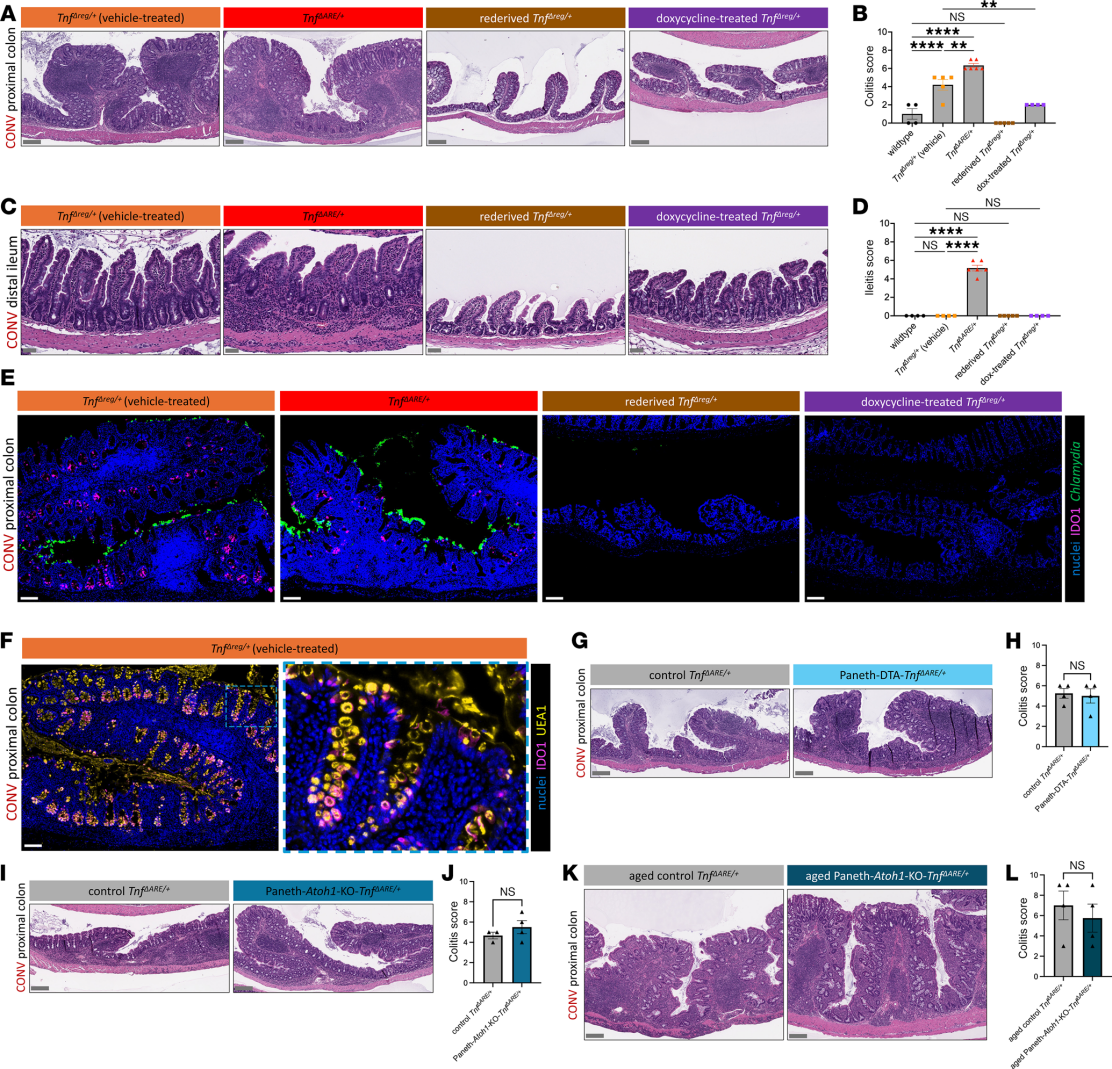
PC inflammation is independent of inflammation and secretory function of the small intestine. (**A** and **C**) Representative H&E-stained PC (**A**) and TI (**C**) sections from age-matched (16–17 weeks) mice from CONV *Tnf^Δreg/+^* (*N* = 5), CONV *Tnf^ΔARE/+^* (*N* = 6), rederived *Chlamydia*-negative CONV *Tnf^Δreg/+^* (*N* = 5), and doxycycline-treated CONV *Tnf^Δreg/+^* (*N* = 4) conditions. Scale bars: 200 μm (**A**), 50 μm (**C**). (**B** and **D**) Colitis scores (**B**) and ileitis scores (**D**) of mice from **A** and **C**. (**E**) Representative IF images of IDO1, *Chlamydia* MOMP, and nuclei costaining of PC sections from CONV *Tnf^Δreg/+^*, CONV *Tnf^ΔARE/+^*, rederived *Chlamydia*-negative CONV *Tnf^Δreg/+^*, and doxycycline-treated CONV *Tnf^Δreg/+^* mice. *N* = 3 mice per condition, age-matched at 16–17 weeks of age at harvest. Scale bars: 100 μm. (**F**) Representative IF image, with inset, of IDO1, UEA1 lectin, and nuclei costaining of PC sections from age-matched (16–17 weeks) CONV *Tnf^Δreg/+^* mice (*N* = 3). Scale bars: 100 μm. Original magnification, ×20. (**G**) Representative H&E-stained PC sections from age-matched (8 weeks) control CONV *Tnf^ΔARE/+^* (*N* = 4) and CONV PC-DTA-*Tnf^ΔARE/+^* (*N* = 4) mice. Scale bars: 200 μm. (**H**) Colitis scores of PCs from **G**. (**I**) Representative H&E-stained PC sections from age-matched (6–10 weeks) control CONV *Tnf^ΔARE/+^* (*N* = 3) and CONV Paneth-*Atoh1*-KO-*Tnf^ΔARE/+^* (*N* = 4) mice. Scale bars: 200 μm. (**J**) Colitis scores of PCs from **I**. (**K**) Representative H&E-stained PC sections from aged (23–64 weeks) control CONV *Tnf^ΔARE/+^* (*N* = 4) and aged CONV Paneth-*Atoh1*-KO-*Tnf^ΔARE/+^* (*N* = 4) mice. Scale bars: 200 μm. (**L**) Colitis scores of PCs from **K**. Statistical significance was determined using an ordinary 1-way ANOVA with Šidák’s multiple-comparison test (**B** and **D**) or an unpaired 2-tailed *t* test (**H**, **J**, and **L**). **P < 0.01, ****P < 0.0001. Data are shown as the mean ± SEM in quantifications. See also Supplemental Figures 9–11.

Given that PC and TI inflammation in the *Tnf*^ΔARE/+^ model occur concomitantly, we asked whether upstream inflammation in the TI is required for *C*. *muridarum–*induced inflammation in the PC. We leveraged a murine model where a genetic insert disrupts the *Tnf* gene and leads to TNF protein expression at an intermediate level between that of WT and *Tnf*^ΔARE/+^ mice (*Tnf*^Δreg/+^, or *Tnf*^ΔAREneo/+^ in ref. 68) (Supplemental Figure 9A). Like *Tnf*^ΔARE/+^ mice, *Tnf*^Δreg/+^ mice develop inflammation in the PC when reared in the CONV facility (Figure 6, A and B, and Supplemental Figure 9B). Importantly, *Tnf*^Δreg/+^ mice did not develop concomitant ileitis, demonstrating independence between TI and PC inflammation (Figure 6, C and D, and Supplemental Figure 9C). Similar to CONV *Tnf*^ΔARE/+^ mice treated with doxycycline, PC inflammation was significantly reduced when CONV facility *Tnf*^Δreg/+^ mice were treated with doxycycline to clear *C*. *muridarum,* and the TI remained uninflamed (Figure 6, A–E, and Supplemental Figure 9, B–D). Moreover, *Tnf*^Δreg/+^ mice in *Chlamydia-*free conditions, achieved by rederivation from sperm into *Chlamydia*-free dams (rederived *Tnf*^Δreg/+^), were expectedly free of *Chlamydia* and their TI and PC were not inflamed, further supporting the role of *C*. *muridarum* in driving PC inflammation (Figure 6, A–E, and Supplemental Figure 9, B–D). The epithelial mechanisms driving inflammation are likely conserved, as evidenced by high expression of IDO1 in PC goblet cells from CONV *Tnf*^Δreg/+^ mice, compared with low IDO1 expression in the PC of doxycycline-treated *Tnf*^Δreg/+^ mice and rederived *Tnf*^Δreg/+^ mice into *Chlamydia-*free conditions (Figure 6, E and F, and Supplemental Figure 9, D and E). These findings demonstrate that inflammation in the PC occurs independently of upstream TI inflammation and is driven by IDO1 expression in goblet cells in response to *C*. *muridarum* colonization in the context of TNF overexpression.

While our results showed that upstream TI inflammation is not required for the development of *C*. *muridarum*–induced colitis, we asked whether antimicrobials produced by small intestinal Paneth cells, which are deposited into mucus that flows into the PC (69–71), would offer protection by modulating *C*. *muridarum* colonization. Loss of Paneth cell function is observed in inflamed CD lesions and has been implicated in pathogenesis (72, 73), and progressive loss of these cells in inflamed regions is characteristic of the *Tnf*^ΔARE/+^ model of intestinal inflammation (74). To determine whether loss of Paneth cell function promotes a bloom of *C*. *muridarum* to drive colonic inflammation, we developed 2 models (*Defa4^Cre/+^ Atoh1^f/fl^* [Paneth-*Atoh1*-KO] and *Defa4^Cre/+^ Rosa^LSL-DTA/+^* [Paneth-DTA]) that achieve long-term ablation of Paneth cells in the *Tnf*^ΔARE/+^ model (Supplemental Figure 10, A–D). In these 2 models, there was no significant difference in colonic inflammation compared with age-matched *Tnf*^ΔARE/+^ controls, indicating Paneth cells do not promote nor protect the PC from inflammation (Figure 6, G–J, and Supplemental Figure 11, A and B). Moreover, Paneth cell–ablated *Tnf*^ΔARE/+^ mice displayed a similar number of *C*. *muridarum* inclusions when compared with control *Tnf*^ΔARE/+^ mice, and IDO1 upregulation was sustained, suggesting Paneth cells do not modulate *C*. *muridarum* colonization (Supplemental Figure 11, C and D). Paneth-*Atoh1*-KO mice exhibited a loss of all secretory cells in the small intestine as a function of age but had no alterations in secretory cell specification in the colon (Supplemental Figure 11, E and F). This model enabled us to specifically examine the role of small intestinal secretory cells in PC inflammation. Colonic inflammation of aged Paneth-*Atoh1*-KO-*Tnf*^ΔARE/+^ mice was similar to that of age-matched control *Tnf*^ΔARE/+^ mice, suggesting that colonic secretory cells, and not small intestinal secretory cells, promote *C*. *muridarum–*induced PC inflammation (Figure 6, K and L, and Supplemental Figure 11G). Taken together, our results suggest that PC inflammation develops independently of inflammation or secretory cell function in the small intestine and instead is driven by specific microbial triggers and colonic goblet cell responses that together drive aberrant inflammatory responses.

### Epithelial IDO1 activation is a hallmark of patients with CD with AC involvement.

We showed that an intracellular microbe *C*. *muridarum* can induce PC inflammation in a genetically susceptible host (TNF overexpression) through goblet cell responses such as IDO1. We next investigated the generalizability of this mechanism in other hosts with immune dysregulation. IL-10 is an antiinflammatory cytokine that is implicated in the pathogenesis of CD, and IL-10 null mice are widely recognized as a colitis model that requires a microbial trigger to induce pathology (26, 75, 76). Consistent with our *Tnf*^ΔARE/+^ model, *Il10rb*^–/–^ mice cohoused with *Chlamydia*-positive CONV facility cagemates developed PC inflammation, while *Il10rb*^–/–^ mice in isolated caging displayed no PC inflammation (Supplemental Figure 12A). Moreover, cohoused *Il10rb*^–/–^ mice harbored *Chlamydia-*colonized PC epithelial cells and had elevated IDO1 expression in epithelial cells compared with isolated caging controls that were *Chlamydia* negative with low expression of IDO1 (Supplemental Figure 12B). Together, these results demonstrate the generalizability of the intracellular microbe, *Chlamydia*, in triggering inflammation specifically in the PC of a host with genetic susceptibility to immune disruptions, such as the IL-10 family cytokine pathways, mirroring mechanisms implicated in human IBD.

**Figure 7:**
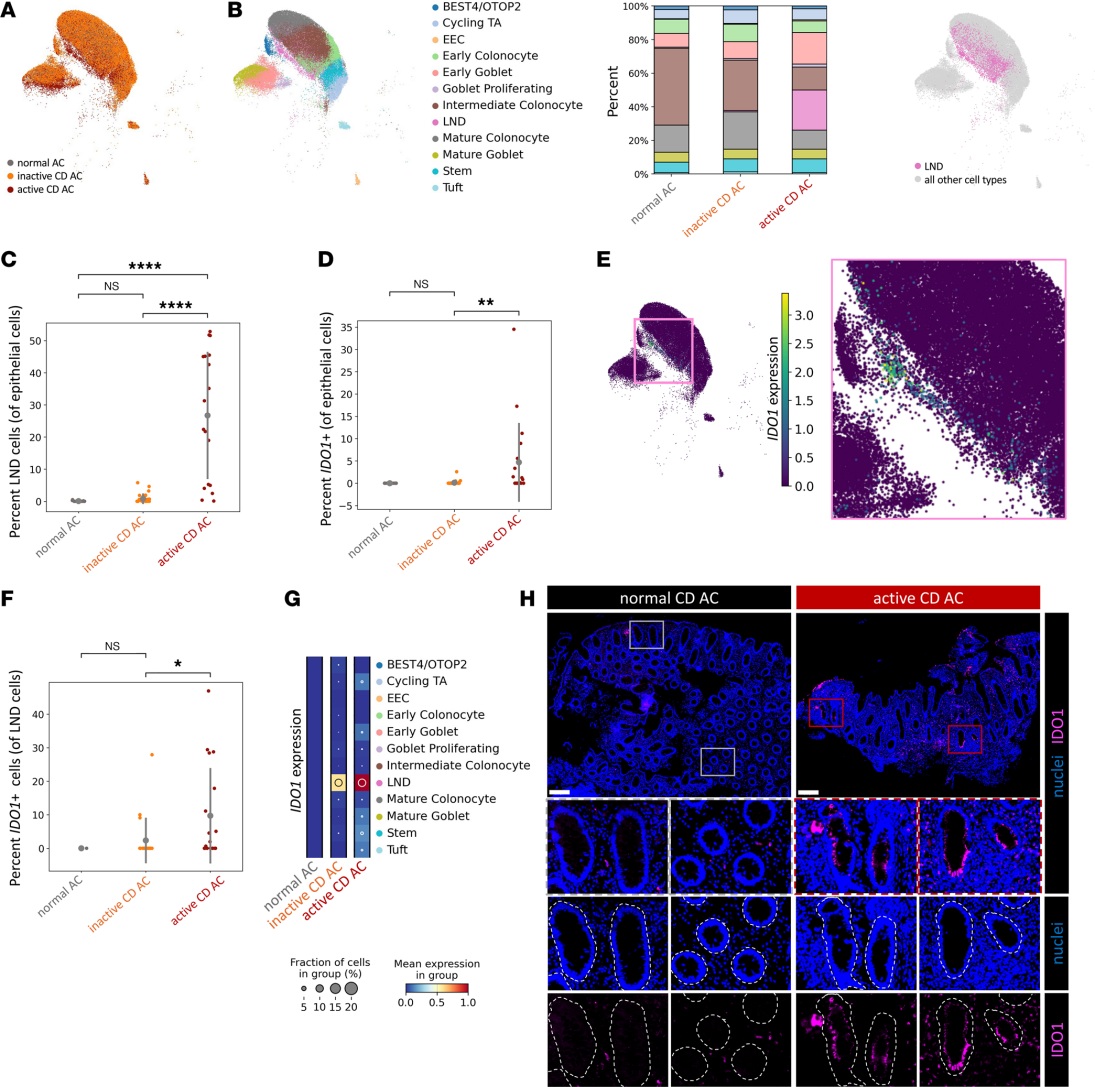
Epithelial IDO1 is upregulated in human CD with active AC inflammation. (**A**) UMAP coembedding of human scRNA-seq data of AC epithelial cells from normal (*N* = 15), inactive CD (*N* = 34), and active CD (*N* = 19) specimens. (**B**) Cell type breakdown of AC scRNA-seq data by UMAP overlay (left and right) and as cell type proportion across sample types (middle). (**C**) Statistical comparison of LND cell proportion amongst all epithelial cells from AC scRNA-seq data. (**D**) Statistical comparison of IDO1-expressing cell proportions amongst all epithelial cells from AC scRNA-seq data. (**E**) UMAP of AC scRNA-seq data with overlay of *IDO1* gene expression. Inset shows *IDO1* expression in the LND cluster. (**F**) Statistical comparison of the proportion of IDO1-expressing cells amongst LND cells from AC scRNA-seq data separated by sample type. (**G**) Cell type–specific *IDO1* expression. (**H**) Representative IF images of IDO1 and nuclei costaining on AC biopsy sections from CD specimens with normal (*N* = 3) or active (*N* = 3) histopathological scoring. Dashed white lines indicate the basal side of epithelial cells. Scale bars: 200 μm. Original magnification, ×20. Data are shown as the mean ± SD in quantifications. Statistical significance was determined using an ordinary 1-way ANOVA with Tukey’s post hoc multiple-comparison test. *P < 0.05, **P < 0.01, ****P < 0.0001. See also Supplemental Figure 12 and Supplemental Table 8.

We next investigated whether IDO1 upregulation is associated with human CD with active AC involvement. We analyzed a human scRNA-seq dataset of CD PC (termed AC in human) and TI specimens, where an emergent population of epithelial cells, termed LND cells (LCN2-, NOS2-, DUOX2-expressing), were identified and associated with CD with active inflammation (Figure 7, A and B, and Supplemental Figure 12C) (77). CD AC specimens with active inflammation had significantly higher proportions of LND cells compared with inactive CD of the AC or normal AC (Figure 7C). In AC specimens, *IDO1* expression was restricted to epithelial cells from CD specimens with a history (active or inactive) of AC inflammation (Figure 7D). Intriguingly*,* *IDO1* was almost exclusively expressed by the LND epithelial cell subpopulation and was significantly higher in patients with active AC inflammation compared with those with inactive AC inflammation (Figure 7, E–G). Using IF microscopy, we confirmed IDO1 protein expression was upregulated in epithelial cells from CD AC samples with active AC inflammation and was not expressed by epithelial cells from CD AC samples without AC inflammation (Figure 7H and Supplemental Figure 12D). Spatially, IDO1-expressing epithelial cells were typically clustered together in isolated crypts and were located in more inflamed regions of the lamina propria, further associating this response with AC inflammation. IDO1 was also detected in the lamina propria of both normal CD AC and actively inflamed CD AC specimens, and *IDO1* was expressed by macrophages in our scRNA-seq data (data not shown), as observed by other groups (78). Notably, *IDO1* expression was largely undetected in TI epithelial cells, further supporting the notion that epithelial IDO1 expression is a region-specific response that drives susceptibility to disease in the AC but not in the TI (Supplemental Figure 12, E–G). Our results demonstrate that like *Chlamydia*-driven upregulation of epithelial IDO1 in mouse models of CD, an analogous upregulation of IDO1 in inflammation-associated LND epithelial cells is a hallmark of active CD in the AC. In addition to the IDO1 response, we examined the expression of goblet cell–specific genes reaching statistical enrichment in ORA of our murine model of AC inflammation (Figure 4F) as well as hallmark IFN-γ response genes and found an enrichment in LND cells, further suggesting conservation of this pathobiont-associated signaling response across species (Supplemental Figure 12H) (79). While IDO1 is chiefly expressed by goblet cells in the murine PC, human IDO1 expression was restricted to the colonic LND epithelial cell subpopulation, a nongoblet, proinflammatory cell type that emerges in CD (77). While the pathobiont trigger and responding cell type may differ between mouse and human, our study generally supports a model whereby pathobionts induce proinflammatory signaling pathways in epithelial cells to drive chronic disease when encountering both anatomical and genetic susceptibilities.

## Discussion

Numerous biologic and small-molecule therapies are currently approved for CD without consideration for the specific GI region involved (80, 81). CD most commonly involves the terminal TI or PC/AC, which are anatomically contiguous, but functionally and biologically distinct organs. Here, we demonstrate that unique secretory cell responses to a pathobiont in the PC drives region-specific, chronic inflammatory disease. Along the GI tract, microbial load and diversity increase, accompanied by a progressively thicker mucus barrier (6). In the TI, Paneth cell–derived antimicrobials help maintain the host-microbe balance. The PC occupies a uniquely vulnerable niche, marked by both loss of Paneth cells and increased bacterial load. Despite enhanced mucin secretion by colonic goblet cells, the mucus barrier in the PC is more penetrable than the distal colon (4, 6), which may render the PC a uniquely vulnerable site prone to pathobiont-triggered inflammation in susceptible hosts. Given luminal continuity between gut segments, one hypothesis is that inflammation-associated TI microbiota flow downstream to seed PC disease. However, our colon-specific model demonstrates that pathobiont-driven PC inflammation occurs independently of upstream ileal disease. Furthermore, Paneth cell depletion, despite the loss of this microbiota-modulating cell type, did not alter PC inflammation. Together, these findings indicate that CD is not simply a general inflammatory disease of the intestine but is shaped by unique and independent cellular and molecular contexts that dictate how microbes affect a particular GI niche.

Pathobionts, normally resident members of the microbiota that become pathogenic under permissive conditions (27), have long been hypothesized as triggers of CD (52). In our CONV colony, *C*. *muridarum* is a resident gut microbe that is typically asymptomatic. We found *C*. *muridarum* is both necessary and sufficient to drive chronic PC inflammation in susceptible hosts via nonbarrier functions of secretory epithelial cells, specifically through tryptophan-modifying pathways. While *Chlamydia* species have not been definitively linked to CD in humans, specific *C*. *trachomatis* serovars are causative agents of proctocolitis that clinically resembles IBD (31, 32). Moreover, *Chlamydia* species frequently colonize the GI tract (82, 83), display tropism for human colonic epithelial organoids (84), and are detectable in phagocytes from intestinal biopsies of patients with CD and from non-IBD controls (85). The lack of a strong association between *Chlamydia* infection and CD in well-powered studies may reflect disease heterogeneity, with involvement limited to a subset of patients. Rather than implicating a single organism, our findings establish a mechanistic link between pathobionts and host epithelial cell responses, supporting a potential role for various microbes to act as region-specific pathobionts in AC disease through the same pathway. Our work also establishes a preclinical model of PC-restricted inflammation to interrogate disease mechanisms, addressing a major gap in CD modeling that goes beyond corroborating reports of *C*. *muridarum* as a reemergent confounder in animal research (54).

We identify a mechanism in which epithelial cells upregulate an immunogenic response to pathobionts, involving IDO1, to drive chronic PC/AC inflammation. While ablating barrier-forming goblet cells to protect against intestinal inflammation seems counterintuitive, accumulating evidence suggests these cells perform functions beyond mucin secretion, acting as context-dependent innate immune signaling hubs (86–90). Accordingly, we show that pathobiont-triggered goblet cell programs (e.g., IDO1, IFN, and antimicrobial pathways) can drive region-specific colitis, implying a trade-off in which suppression of pathogenic innate immune signaling outweighs the loss of mucin-mediated barrier function.

While IDO1 upregulation has been observed in bulk tissue from inflamed human IBD specimens (45–47), our study extends these observations by revealing that epithelial IDO1 upregulation is mediated by LND cells, a CD-associated proinflammatory signaling hub (77). Furthermore, we show that epithelial IDO1 upregulation is specific to CD of the AC, but not of the TI, consistent with the regional specificity we observed in our CD model. While we did not delineate the full downstream immune cascade, we propose that chronic, pathobiont-driven inflammation arises from IDO1-mediated remodeling of the microenvironment through altered tryptophan availability and engagement of the kynurenine pathway (91). IDO1 activity depletes tryptophan, restricting this essential amino acid from metabolically demanding cells, which in T cells, results in reduced proliferation and dysfunction (92, 93). In addition to tryptophan depletion, IDO1 generates kynurenine, which activates AHR to promote Treg differentiation and immunosuppression (41–44), whereas downstream kynurenine metabolites (e.g., kynurenic acid, picolinic acid, and quinolinic acid) can exert diverse, context-dependent immune effects. While Tregs are known for suppressing an active immune response, elevated Tregs are frequently observed in inflamed IBD mucosa despite uncontrolled inflammation (94, 95). Recent work has identified Tregs that are proinflammatory in CD-associated inflammation (96, 97). Although adoptive Treg therapy is being explored to suppress inflammation in CD (98), our findings argue for caution as this specific immunosuppressive strategy may impair clearance of pathobionts and instead promote persistent inflammation. In contrast, targeting immunometabolic checkpoints such as epithelial innate immune signaling via IDO1 may constitute a more precise approach. Future CD therapies should balance immune suppression with microbial control and be tailored to context- and region-specific disease mechanisms.

We acknowledge the following limitations of this study. Most experiments used the *Tnf*^ΔARE/+^ mouse, a well-established TI CD model (51) that we show also develops PC inflammation. There are known and unknown differences between this model and human CD. Although the *Tnf*^ΔARE/+^ mouse lacks skip lesions, we argue for its translational relevance; the presence of these lesions is a function of organ size and not biology, as they are present in the porcine *Tnf*^ΔARE/+^ model with the same mutation (99). The model is driven by elevated TNF overexpression and may only capture a subset of AC CD, reflecting heterogeneity of patient response to anti-TNF therapy (100, 101). Moreover, our study focuses on response to a specific pathobiont, *C*. *muridarum*, and the relevance of its human counterpart or of specific AC pathobionts remains to be defined.

## Methods

### Sex as a biological variable.

Both male and female mice were used in experiments, and findings were similar unless otherwise noted. Both male and female human specimens were examined, and findings were similar unless otherwise noted.

### Statistics.

Data were analyzed using GraphPad Prism (v10.2.2) and R (v4.1.1) with various packages and associated dependencies listed in the Key Resources Table in Supplemental Methods. Specific tests, thresholds, and parameters used for each statistical test are described in figure legends. *P* values of less than 0.05 were considered significant. Unless otherwise stated, data represent the mean ± SEM.

### Study approval.

All murine experiments were performed under protocols approved by the Vanderbilt University Medical Center Animal Care and Use Committee and in accordance with NIH guidelines. The human work described in this study was conducted through The Gut Cell Atlas study protocol and was approved by the Institutional Review Board at Vanderbilt University Medical Center (IRB 191738). Written informed consent was obtained from subjects with CD and those considered control (non-IBD) to obtain TI and AC tissues at the time of scheduled endoscopic procedures. All samples were obtained as a part of the clinical trial Combinatorial Single Cell Strategies for a Crohn’s Disease Gut Cell Atlas (ClinicalTrials.gov NCT04113733).

### Data availability.

The sequencing data used in this manuscript are accessible in the Gene Expression Omnibus under GSE284294 (murine scRNA-seq) and GSE266546 (human scRNA-seq). Other data are available upon request to the corresponding author. Values for all data points in graphs are reported in the Supporting Data Values file.

For further details of methods, see Supplemental Methods.

## Author contributions

Conceptualization, PNS and KSL; data curation, PNS, EPS, AJS, KTW, LAC, QL, RHV, and KSL; formal analysis, PNS, EPS, MEB, WK, YY, HK, KDM, and KSL; funding acquisition, NOM, KTW, LAC, JAG, QL, MKW, RHV, WZ, and KSL; investigation, PNS, JW, EPS, LS, AJS, TK, WK, MEB, YY, HK, YX, SWK, MDH, MAL, LZ, DA, NT, KDM, OSK, MHH, JR, JL, AB, MKW, and KSL; methodology, PNS, EPS, LS, AJS, MEB, YY, HK, MDH, KDM, JAG, MKW, RHV, WZ, and KSL; project administration, PNS, EPS, LS, AJS, KTW, LAC, JAG, QL, MKW, RHV, WZ, and KSL; software, PNS, EPS, YY, HK, MDH, KDM, QL, and KSL; resources, NOM, KTW, LAC, JAG, QL, MKW, RHV, WZ, and KSL; supervision, KTW, LAC, JAG, QL, MKW, RHV, WZ, and KSL; validation, PNS, MEB, EPS, KDM, RHV, and KSL; visualization, PNS, JW, EPS, LS, MEB, WK, YY, HK, SWK, MAL, KDM, and KSL; writing, PNS and KSL.

## Funding support

This work is the result of NIH funding, in whole or in part, and is subject to the NIH Public Access Policy. Through acceptance of this federal funding, the NIH has been given a right to make the work publicly available in PubMed Central.

- NIH grants R01DK103831 and U54CA274367 (to KSL).
- NIH grants F31DK127687 and T32HD007502 (in support of PNS).
- NIH grants R01DK134692 and R35GM147470 (to WZ).
- NIH grant R01DK128200 (to KTW).
- NIH grant R21AI173599 (to RHV).
- Howard Hughes Medical Institute GT16446 (to DA and KSL).
- National Science Foundation Graduate Research Fellowship 2444112 (to MEB).
- Stanley Cohen Innovation Fund (to KSL).
- The Leona M. and Harry B. Helmsley Charitable Trust G-1903-03793 (to the Vanderbilt University Medical Center Gut Cell Atlas, of which KSL, LAC, and KTW are members).
- Crohn’s & Colitis Foundation Senior Research Award 1061046 (to JAG).
- VA Merit grants I01CX002473 (to KTW) and I01CX002662 (LAC).
- Core support from NIH: P30CA068485 and P30DK058404.

## Supplementary Material

Supplemental data

Unedited blot and gel images

Supplemental table 1

Supplemental table 2

Supplemental table 3

Supplemental table 4

Supplemental table 5

Supplemental table 6

Supplemental table 7

Supplemental table 8

Supporting data values

## Version 1 02/17/2026

In-Press Preview

## Version 2 04/01/2026

Electronic publication
