# Supplementary material for: Pathobiont-triggered induction of goblet cell response drives regional susceptibility to inflammatory bowel disease
Source: J Clin Invest. 2026 Feb 17;136(7):e201729. doi: 10.1172/JCI201729 (PMC13038202; doi:10.1172/JCI201729)
Supplement: Supplemental data [file jci-136-201729-s098.pdf]

SUPPLEMENTAL METHODS

KEY RESOURCES TABLE

REAGENT or RESOURCE	SOURCE	IDENTIFIER
**Antibodies**	**Source**	**Identifier**
Goat polyclonal anti- Chlamydia major outer membrane protein (MOMP)	Novus Biologicals	Cat#NB100-65054; RRID: AB_962905
Alexa Fluor 647 Rabbit Anti-DCAMKL1 (Clone EPPR6085)	Abcam	Cat#ab202755
Alexa Fluor 647 Rabbit Anti-CLCA1 (Clone EPR12254-88)	Abcam	Cat#ab203735
CoraLite Plus 488-conjugated Rabbit Chromogranin A Polyclonal antibody	Proteintech	Cat#CL488-10529; RRID: AB_2883077
Rabbit monoclonal anti-IDO1 (Clone D8W5E)	Cell Signaling Technology	Cat#51851; RRID: AB_2799402
Rabbit polyclonal anti-GFP	Novus Biologicals	NB600-308
Donkey anti-Goat IgG (H+L) Cross-Adsorbed Secondary Antibody, Alexa Fluor™ 488	Invitrogen	A11055
Donkey anti-Rabbit IgG (H+L) Highly Cross-Adsorbed Secondary Antibody, Alexa Fluor™ 568	Invitrogen	A10042
Donkey anti-Rabbit IgG (H+L) Highly Cross-Adsorbed Secondary Antibody, Alexa Fluor™ 647	Invitrogen	A31573
Donkey anti-Goat IgG (H+L) Cross-Adsorbed Secondary Antibody, Alexa Fluor™ 647	Invitrogen	A21447
**Bacterial and virus strains**		
CM001-GFP	Laboratories of Raphael Valdivia (this study)	
Cm-VU	This Study	
**Chemicals, peptides, and recombinant proteins (animal treatments)**		
Tryptophan-deficient diet	Inotiv	TD.230608
Control diet	Inotiv	TD.07788
Standard chow	LabDiet	5L0D
Tamoxifen	MP Biomedicals	CAS: 10540-29-1
Sucrose	Fisher Chemical	Cat: S3-212
Doxycycline	Sigma-Aldrich	Product #: D9891; CAS #: 24390-14-5
**Critical commercial assays**		
Mouse TNF-alpha Quantikine ELISA Kit	R&D Systems	MTA00B
Pierce BCA Protein Assay Kit	Thermo Fisher Scientific	23225
HotStarTaq Plus Master Mix Kit	Qiagen	203643
DNeasy Blood and Tissue Kit	Qiagen	Cat# 69504
Qubit dsDNA Quantification Assay Kit	Invitrogen	Cat# Q32853
Nanopore Rapid Barcoding Kit 96 V14	Oxford Nanopore Technologies	Cat# SQK-RBK114.96
SeqWell ExpressPlex 96 kit	seqWell	Cat# 301098
Qubit-iT 1X dsDNA HS Assay Kit	Thermo Fisher Scientific	Q33230
DNeasy PowerLyzer PowerSoil Kit	Qiagen	12855
**Deposited data**		
scRNA-seq data for *Tnf*$^{\Delta ARE/+}$ mice	This Study	GSE284294

scRNA-seq for human CD	Laboratories of Keith Wilson, Lori Coburn, Ken Lau, and Qi Liu (1)	GSE266546
**Experimental models: Cell lines**		
Vero	ATCC	CCL-81
McCoy	ATCC	CRL-1696
**Experimental models: Organisms/strains**		
B6.129S-Tnf^{tm2Gkl}*/Flmg* (common name: *Tnf*$^{\Delta ARE/+}$)	Laboratory of Kevin Haigis (2)	EM:04974
B6;129S-Tnf$^{tm2.1Gkl}$*/Flmg* (common name: *Tnf*$^{\Delta AREneo}$ and termed *Tnf*$^{\Delta reg/+}$ and *Tnf*$^{\Delta reg/\Delta reg}$ in this paper)	EMMA Greek Node (2, 3)	EM:11081
Defa4^{Cre}	Laboratory of Peter Dempsey (4)	N/A
B6.129P2-*Gt(ROSA)26Sor*$^{tm1(DTA)Lky}$/J (common name: *Rosa*$^{LSL\text{-}DTA}$)	Laboratory of Robert Coffey	RRID: IMSR_JAX:009669
Gt(ROSA)26Sor$^{tm4/ACTB\text{-}tdTomato,\text{-}EGFP)Luo}$/J (common name: *Rosa*^{mTmG})	Jackson Labs	RRID: IMSR_JAX:007576
B6.129S7-*Atoh1*^{tm3Hzo}/J (common name: *Atoh1*^{flox})	Jackson Labs	RRID:IMSR_JAX:008681
Lrig1$^{tm1.1(cre/ERT2)Rjc}$*/J* (common name: *Lrig1*^{CreERT2})	Jackson Labs (5)	RRID:IMSR_JAX:018418
C57BL/6N-*Krt20*$^{em1(cre/ERT2)Amc}$/J (common name: *Krt20*^{CreERT2})	Jackson Labs	RRID: IMSR_JAX:030600
C57BL/6J	Jackson Labs	RRID: IMSR_JAX:000664
B6.129S2-*Il10rb*^{tm1Agt}/J (common name IL-10rb$^{-/-}$)	Laboratory of Jeremy Goettel	RRID:IMSR_JAX:005027
**Oligonucleotides**		
CTTGGCATTGACAGGCGA	Laboratory of Neil Lipman (6)	CHLSE37
GGAGAGTGGTCTCCCCAGATT	Laboratory of Neil Lipman (6)	CHLSE461
**Software and algorithms**		
Seurat	Laboratory of Rahul Satija (7)	https://satijalab.org/seurat/
Python	Python	https://www.python.org/
RStudio version 4.1.1	RStudio	https://posit.co/downloads/
SCANPY	Laboratory of Fabian Theis (8)	https://github.com/scverse/scanpy
Biorender	Biorender	https://www.biorender.com/
Prism	GraphPad	https://www.graphpad.com/features
Scikit-bio	Maxime Borry (9)	https://scikit.bio/index.html
One Codex	One Codex (10)	https://www.onecodex.com/
Nikon Elements	Nikon	https://www.microscope.healthcare.nikon.com/products/software/nis-elements
QuPath version 0.5.1	Laboratory of Peter Hamilton (11)	https://qupath.github.io/
**Other**		
UEA-1 lectin	Sigma-Aldrich	L8262-25
Streptavidin, Alexa Fluor™ 750 conjugate	Thermo Fisher Scientific	S21384
Streptavidin, Alexa Fluor™ 488 Conjugate	Thermo Fisher Scientific	S32354
HCR Probe Hybridization Buffer	Molecular Instruments	PBH03524
HCR Probe Wash Buffer	Molecular Instruments	BPW03224
HCR Amplification Buffer	Molecular Instruments	BAM03924

Proteinase K	Thermofisher	01044666
20X SSC	Invitrogen	15557-044
Probe: CM_23S	Molecular Instruments	RTE679
Amplifier B1-546	Molecular Instruments	N/A
Probe: Muc2	Molecular Instruments	NM_023566.3
Amplifier B3-488	Molecular Instruments	N/A
4% paraformaldehyde	Thermo Fisher Scientific	J19943-K2
Histoclear	National Diagnostics	HS-200
Triton X-100	Sigma	9002-93-1
Target Retrieval Solution, Citrate pH 6.1 (10x)	Agilent Dako	S1699
Bovine Serum Albumin	Sigma-Aldrich	A9418
Normal Donkey Serum	Jackson Immuno Research	017-000-121
Hoechst	Invitrogen	H3570
Prolong Gold AntiFade Reagent	Thermo Fisher Scientific	P36930
Glycerol	Invitrogen	15514-011
Water, Nuclease Free	Promega	P1193
Quick-Load® Purple 1 kb Plus DNA Ladder	New England Bio Labs	N0550S
0.5M EDTA pH 8	Corning	46-034-CI
1M HEPES	Corning	25-060-CI
10X DPBS	Boston BioProducts Inc	BM-220
Fetal Bovine Serum	Sigma-Aldrich	F4135
Protease from *Bacillus licheniformis*	Sigma-Aldrich	P5380
Deoxyribonuclease (DNase) I from bovine pancreas	New England Biolabs	M0303S
1x DNase I buffer	New England Biolabs	B0303S
DTT	Teknova	D9750
1X NP-40 Lysis High Salt Buffer Solution	RPI Corp.	N320000
100X Halt™ Protease and Phosphatase Inhibitor Single-Use Cocktail	Thermo Fisher Scientific	78442
KH_2PO_4	Thermo Fisher Scientific	BP362500
DPBS w/o Calcium & Magnesium 1x	Thermo Fisher Scientific	MT21031CV
Water, Cell Culture Grade	Sigma-Aldrich	W3500
L-Glutamic acid	Millipore Sigma	G1251
DMEM, High Glucose w/ Pyruvate and L-Glutamine	Gibco	11995065
DMEM	Gibco	11965092
PBS without Calcium or Magnesium, pH 7.4	Gibco	10010023
Trypsin-EDTA (0.25%), phenol red	Gibco	25200056

EXPERIMENTAL MODEL AND STUDY PARTICIPANT DETAILS

Human Specimens from The Gut Cell Atlas

The Gut Cell Atlas (GCA) study protocol was approved by the Institutional Review Board at Vanderbilt University Medical Center (IRB #191738). Written informed consent was obtained from non-IBD control and CD subjects to obtain TI and AC tissues at the time of scheduled endoscopic procedures. All samples were obtained as a part of the clinical trial "Combinatorial Single Cell Strategies for a Crohn's Disease Gut Cell Atlas", identifier NCT04113733 (clinicaltrials.gov).

Between December 2019 and July 2023, endoscopy subjects were prospectively recruited in the IBD clinic or GI endoscopy unit at Vanderbilt University Medical Center prior to colonoscopy for CD disease activity assessment or non-IBD indications including colorectal cancer screening or polyp surveillance. Exclusion criteria for the study were: pregnancy, known coagulopathy or bleeding disorders, known renal or hepatic impairment, history of organ transplantation, or inability to give informed consent. For all participants, demographics including age, gender, medical history, and medication use were determined from participant reporting and review of the electronic medical record. Patient metadata is available in Table S8.

Tissue biopsies in the TI and AC for immediate scRNA-seq data generation were placed in chelation buffer (4 mM EDTA, 0.5 mM DTT in PBS) as previously described (1). Tissue biopsies for later DNA extraction were flash frozen on dry ice and stored at -80°C. Tissue biopsies for formalin-fixed and paraffin-embedded (FFPE) tissue block generation underwent standard fixation and paraffin embedding protocols. 5 μm sections were used from each FFPE block, stained with hematoxylin and eosin (H&E), and examined in a blinded manner by a gastrointestinal pathologist (M.K.W.) and graded accordingly as: inactive (normal_CD or quiescent) or active (mild, moderate, or severe activity). All associated study data were collected and managed using Research Electronic Data Capture (REDCap) electronic data capture tools hosted at Vanderbilt (12, 13), including Clinical Data Interoperability Services, such as Clinical Data Pull (14) and e-consent (15).

Murine Housing

Mice were housed (up to 5 per cage) in a conventional (CONV), specific pathogen-free barrier (SPF-B), or ABSL-2 environment under a standard 12-hour daylight cycle, and were fed a standard irradiated chow (PicoLab® Laboratory

Rodent Diet 5L0D) and provided water ad libitum, unless otherwise specified. Littermate controls of both sexes were used for experiments when possible. Housing facility conditions are detailed below.

CONV Murine Housing Facility: Mice were procured from approved commercial vendors or non-commercial sources that meet facility health requirements after a period of quarantine and testing. Mice were housed in individual ventilated cages. Acidified municipal water is provided via automatic watering valves. Traffic flow of animals was two-way (i.e., mice may leave the facility for procedures and return to the housing room). PPE required to work in Level 5 rooms in this facility includes gowns and gloves. Excluded pathogens: Mouse Hepatitis Virus, Mouse Parvovirus, Minute Virus of Mice, Lymphocytic Choriomeningitis Virus, Sendai Virus, Pneumonia Virus of Mice, Epizootic Diarrhea of Infant Mice, Theiler's Mouse Encephalomyelitis Virus, Mouse Pox, Mouse Adenovirus, Mouse Reovirus, *Mycoplasma pulmonis*, *Syphacia* spp, *Aspiculuris* spp, *Myobia musculi*, *Radfordia affinis*, *Myocoptes musculinus*, *Psorergates simplex*.

SPF-B Murine Housing Facility: Mice were procured only from approved commercial vendors or via rederivation. Mice were housed in autoclaved individual ventilated cages. Autoclaved reverse osmosis water was provided in water bottles. The SPF-B facility may not be entered after working in any other mouse facility. Traffic flow of animals is one way from the facility (i.e., mice may not return if they leave the facility for procedures). All equipment and supplies must be dedicated to this facility. PPE required to work in the facility includes gowns, gloves, caps, masks, and shoe covers. Excluded pathogens include those in the CONV facility as well as mouse Norovirus and *Helicobacter* spp.

Animal Biosafety Level 2 (ABSL-2) Murine Housing Facility:

Rooms were dedicated for mice experimentally infected with agents that require ABSL-2 housing conditions. Mice were purchased directly from approved vendors and delivered to ABSL-2 housing rooms or transferred from other SPF-B housing rooms. Mice were housed in individual ventilated cages and provided reverse osmosis water in bottles. All handling of animals was conducted in biosafety cabinets and animals were not transferred to other housing areas from ABSL-2 rooms. PPE required to work in ABSL-2 areas includes gowns, double gloves, and masks.

Murine models and experiments

Tnf$^{\Delta ARE/+}$ and wildtype (*Tnf*$^{+/+}$) mice were housed in CONV, SPF-B, or ABSL-2 facilities as described above. Mice were maintained on a C57BL/6J background by backcrossing to breeders purchased from Jackson Labs. Genotyping of the mice was performed through Transnetyx (Cordova, TN, USA). Other alleles on C57BL/6J backgrounds were bred into the *Tnf*$^{\Delta ARE/+}$ mouseline as described below. Weights were monitored to ensure mice were euthanized at humane endpoints. Unless otherwise specified, equal representation of male and female mice were used in all experiments wherever possible.

Genetic backgrounds of the mice were tested using the miniMUGA array performed through Transnetyx (Cordova, TN, USA) (16).

For co-housing experiments, SPF-B wildtype and *Tnf*$^{\Delta ARE/+}$ mice were transferred to the CONV facility and placed into fresh cages with CONV facility, *Chlamydia muridarum*-positive wildtype or *Tnf*$^{\Delta ARE/+}$ cagemates. This experiment was performed at various ages, with same-sex or mixed-sex conditions, and harvested at various ages. If transferred as pups, the SPF-B litter was transferred by postnatal day 3, combined/co-fostered with a CONV wildtype or *Tnf*$^{\Delta ARE/+}$ dam with similar age litter, and monitored. Genotyping for transgenes or mutated alleles was used to distinguish CONV and SPF-B pups in the mixed litters. Mice were euthanized and tissue was harvested at timepoints and ages indicated in figure legends.

For isolated caging experiments, adult SPF-B wildtype and *Tnf*$^{\Delta ARE/+}$ mice were transferred to the CONV facility and placed into fresh cages without CONV facility cagemates. Mice and cage changes were handled with fresh gloves, a clean hood, and clean materials to minimize transfer of microbiota from CONV facility mice. Mice were euthanized and tissue was harvested at timepoints and ages indicated in figure legends.

For doxycycline treatment experiments, adult *C. muridarum*-positive CONV mice were given vehicle (5% sucrose) or doxycycline (5% sucrose, 2 mg/mL doxycycline) water ad libitum for 1-2 weeks, as indicated in figure legends. To minimize re-inoculation with *C. muridarum* from fecal matter, cages were changed at the start of the experiment and upon cessation of vehicle or doxycycline treatment. Mice and cage changes were handled with fresh gloves, a clean hood, and clean materials to minimize transfer of microbiota from other cages. Mice were treated at various ages as stated in figure legends. Fecal samples were collected throughout the experiment including before treatment, within one week after cessation of treatment, and at harvest. Mice were euthanized and tissue was harvested at timepoints and ages indicated in figure legends.

For CM001-GFP inoculation experiments, adult SPF-B mice were transferred to the ABSL-2 facility. Mice and cage changes were handled with fresh gloves, a clean hood, and clean materials to minimize transfer of microbiota from other cages. Mice were administered a single treatment of sham or CM001-GFP inoculate via oral gavage in 100 μL volume. CM001-GFP ($3x10^6$ IFUs) inoculate was prepared from CM001-GFP-infected Vero cell lysate and sham inoculate was prepared from uninfected Vero cell lysate, both prepared in 1X SPG (sucrose-phosphate-glutamate) buffer (219 mM sucrose, 3.7 mM KH_2PO_4, 4.9 mM L-glutamic acid in cell-grade water to pH 7.4-7.6 using NaOH). Fecal samples were collected throughout the experiment including before treatment, at timepoints post-treatment, and at harvest. Mice were euthanized and tissue was harvested at timepoints and ages indicated in figure legends. Weight change was calculated at harvest as a percent of the individual mouse's weight prior to treatment.

For *Lrig1-Atoh1*-KO-$Tnf^{\Delta ARE/+}$ experiments, tamoxifen was administered to SPF-B mice in experimental ($Lrig1^{CreERT2/+}$;$Atoh1^{fl/fl}$;$Tnf^{\Delta ARE/+}$) or control ($Lrig1^{+/+}$;$Atoh1^{fl/fl}$;$Tnf^{\Delta ARE/+}$) conditions via intraperitoneal injection of 2 mg of tamoxifen for 4 consecutive days (8 mg total dose). Seven days after the first injection, mice were transferred to the CONV facility and co-housed with *C. muridarum*-positive CONV $Tnf^{\Delta ARE/+}$ cagemates for 3 weeks. Mice were euthanized and tissue was harvested at timepoints and ages indicated in figure legends.

For *Krt20-Atoh1*-KO-$Tnf^{\Delta ARE/+}$ experiments, tamoxifen was administered to CONV mice in experimental ($Krt20^{CreERT2/+}$;$Atoh1^{fl/fl}$;$Tnf^{\Delta ARE/+}$) or control ($Krt20^{+/+}$;$Atoh1^{fl/fl}$;$Tnf^{\Delta ARE/+}$) conditions via two intraperitoneal injections per week of 6.25 mg of tamoxifen for 3 weeks (37.5 mg total dose). Mice were euthanized and tissue was harvested three weeks after the first injection at ages indicated in figure legends.

For tryptophan-deficient diet experiments, CONV $Tnf^{\Delta ARE/+}$ mice were given synthetic diets that were tryptophan-deficient or 0.18% tryptophan (control) ad libitum. Mice were euthanized and tissue was harvested after four weeks of treatment at ages indicated in figure legends.

For rederivation of the *Tnf*$^{\Delta reg}$ line in *C. muridarum*-free CONV facility conditions, *in vitro* fertilization was performed using *Tnf*$^{\Delta reg/+}$ sperm and C57BL/6J eggs. Embryos were transferred into superovulating dams. Weanlings were transferred to the CONV facility and kept in isolated caging conditions, as described above.

For Paneth-DTA-*Tnf*$^{\Delta ARE/+}$ experiments, CONV mice of experimental (*Defa4*$^{Cre/+}$*;Rosa*$^{LSL\text{-}DTA/+}$*;Tnf*$^{\Delta ARE/+}$) or control (*Defa4*$^{+/+}$*;Rosa*$^{LSL\text{-}DTA/+}$*;Tnf*$^{\Delta ARE/+}$ or *Defa4*$^{+/+}$*;Rosa*$^{+/+}$*;Tnf*$^{\Delta ARE/+}$) genotypes were euthanized and tissue was harvested at ages indicated in figure legends.

For Paneth-*Atoh1*-KO-*Tnf*$^{\Delta ARE/+}$ experiments, CONV mice of experimental (*Defa4*$^{Cre/+}$*;Atoh1*$^{fl/fl}$*;Tnf*$^{\Delta ARE/+}$) or control (*Defa4*$^{+/+}$*;Atoh1*$^{fl/+}$*;Tnf*$^{\Delta ARE/+}$, *Defa4*$^{Cre/+}$*;Atoh1*$^{fl/+}$*;Tnf*$^{\Delta ARE/+}$, or *Defa4*$^{+/+}$*;Atoh1*$^{fl/fl}$*;Tnf*$^{\Delta ARE/+}$) genotypes were euthanized and tissue was harvested at ages indicated in figure legends.

For *Il10rb*$^{-/-}$ experiments, *C. muridarum*-free *Il10rb*$^{-/-}$ CONV mice were either kept in isolated caging (control condition) or co-housed with *C. muridarum*-positive *Tnf*$^{\Delta ARE/+}$ cagemates (experimental condition) for 4-8 weeks. Mice were euthanized and tissue was harvested at ages indicated in figure legends.

For scRNA-seq experiments, wildtype or *Tnf*$^{\Delta ARE/+}$ mice were harvested from SPF-B or CONV facilities at young (6-7w) or aged (20-24w) conditions. The CONV wildtype condition used a mixture of young and aged mice.

DNA extraction and shotgun metagenomic sequencing of murine intestinal luminal contents

Mouse small intestine and colons were harvested using sterilized tools. The terminal ileum, defined as the last twelfth of the small intestine, and the proximal colon, defined by the first third of the colon, were separately isolated. The contents of the each segment were flushed using sterile PBS pH 7.4 without Calcium or Magnesium into individual barcoded tubes containing DNA stabilization buffer to ensure reproducibility, stability, and traceability, and shipped for DNA extraction, library preparation, and sequencing by Transnetyx (Cordova, TN USA). DNA extraction was optimized and fully automated using a robust process for reproducible extraction of inhibitor-free, high molecular weight genomic DNA that captures the true microbial diversity of samples. DNA extractions were subjected to quality control (QC) steps. Next, genomic DNA was converted into sequencing libraries using a method optimized for minimal bias. Unique dual indexed (UDI) adapters

were used to ensure that reads and/or organisms are not mis-assigned. After QC, the libraries were sequenced using the shotgun sequencing method (a depth of 2 million 2x150 bp read pairs), which enables species and strain level taxonomic resolution.

Shotgun metagenomic sequencing analysis

Sequencing data were uploaded automatically onto One Codex analysis software and analyzed against the One Codex database consisting of ~148K complete microbial reference genomes (10). The classification results were filtered through several statistical post-processing steps designed to eliminate false positive results caused by contamination or sequencing artifacts. Host reads were identified using mouse and human genomes. The relative abundance of each microbial species is estimated based on the depth and coverage of sequencing across every available reference genome. Only reads for the eubacteria kingdom were considered in analysis of shotgun metagenomic data.

Murine scRNA-seq data generation

Colons were dissected and trisected. The proximal colon, defined by the first third of the colon, was isolated and luminal content was flushed using PBS without calcium or magnesium. Next, the tissue was flayed open, washed twice in PBS without calcium and magnesium, and placed into cold chelation buffer consisting of 3 mM EDTA (Corning), 20 mM HEPES (Corning), and 0.5 mM DTT (Teknova) in PBS without calcium or magnesium for 1 hour at 4°C. Chelation buffer was discarded and colonic epithelium (crypts) were collected by vigorously shaking the colonic tissue in PBS. The shaking process was repeated for a total of four times and the pooled crypt isolates were passed through a 100 µm filter. Crypts were dissociated into single cells using cold protease buffer consisting of 5 mg/mL Protease from *Bacillus licheniformis* (Sigma-Aldrich) and 2.5 mg/mL DNase (Sigma-Aldrich) in PBS without calcium or magnesium on a rotator for 25 minutes at 4°C. After dissociation, the suspension was mechanically dissociated into single cells by gentle pipetting with a wide bore pipette tip. The solution was passed through a 70 µm filter and resultant single cells were quenched with 2% FBS. A series of washes were performed to obtain a single-cell suspension with mostly live cells and minimal debris. Single-cell encapsulation, library preparation, and sequencing were performed as previously described (17, 18).

scRNA-seq data alignment and filtering

For human scRNA-seq data, alignment and filtering were described previously (1). For murine scRNA-seq data, raw sequencing data were aligned using dropEst to mouse GRCm38.85 resulting in count matrices (19). Resultant count matrices were subjected to filtering protocols as follows. First, cells were ranked according to number of transcripts (counts) detected and an inflection point cutoff was used to filter out cells with low counts. Next, cells were projected in 2D space using UMAP embedding and overlays of total counts, percent mitochondrial reads, and cell type specific markers were used to iteratively remove low-quality cells.

scRNA-seq data analysis

Analysis of pre-processed murine scRNA-seq data was carried out Seurat version 4.0.4 as described previously (7, 20). Functions use default arguments unless specified. Batch effects were minimal as seen from the intermixing of cell types, and therefore, no batch corrections were performed. Briefly, Seurat's clustering (FindClusters) function with iterative adjustment of the clustering resolution parameter was used along with differential expression analysis (FindMarkers) to label cell types based on marker gene expression. Differential expression analysis (FindMarkers) was performed to identify differences between cell types and across conditions, using log fold change of 1 or 1.5, only positive (upregulated) genes, and all other standard parameters. ORA was performed using WebGestalt web-based tool with the geneontology/biological process functional database (21).

Analysis of pre-processed human scRNA-seq data was carried out in SCANPY (8) with UMAP embeddings and cell type annotations as described previously (1). Normal AC is composed of healthy control specimens, inactive CD AC is composed of CD specimens histopathologically scored as normal or quiescent, and active CD AC is composed of CD specimens histopathologically scored as mild, moderate, or severe.

Murine intestinal tissue fixation, embedding, and staining

Intestinal tissue was dissected, flushed with PBS, flayed longitudinally onto Whatman filter paper, and fixed in 4% PFA (Thermo Scientific) for approximately 24 hrs at room temperature. Fixed tissues were washed with PBS, swiss-rolled, stabilized in 2% agar, and stored in 70% EtOH until processing and paraffin embedding to generate formalin-fixed, paraffin embedded (FFPE) blocks. FFPE blocks were sectioned at 5 µm thick onto charged glass slides. Slides were incubated in Histoclear (National Diagnostics) solution for deparaffinization. Next, slides were rehydrated by stepped incubation in 90%,

70%, and 50% ethanol solutions, followed by permeabilization in 0.3% Triton X-100 (Sigma). Next, slides were antigen retrieved in citrate buffer (Dako) for 20 minutes in a pressure cooker at 105°C followed by a 20-minute bench cool down. For IF staining, slides were blocked for 1+ hour in a humidified chamber in blocking buffer consisting of 2.5% Normal Donkey Serum, 1% BSA, 0.3% Triton X-100, 1:10,000 Hoechst 33342 in PBS prior to antibody staining. Primary antibodies were incubated on the slides in a humidified chamber for 1+ hour, followed by three washes in PBS. Compatible conjugated secondaries, if applicable, were incubated on the slides in a humidified chamber for 1 hour, followed by three washes in 1X PBS. Slides were mounted in Prolong Gold mounting media (Thermo Fisher Scientific) or 50% glycerol in PBS. For histological analysis, slides were processed and stained for hematoxylin and eosin using standard approaches. For RNA-FISH, Molecular Instruments HCR™ RNA-FISH protocol was adapted as described previously (1) and used with custom probes designed for *Muc2* or *C. muridarum 23S* RNA on FFPE tissue sections (22).

Brightfield and fluorescence imaging

Brightfield whole slide images were imaged on a Leica Aperio AT2 automated slide scanner (Leica Biosystems) at 20x magnification to a resolution of 0.273 µm /pixel. Immunofluorescent tissue sections for whole slide imaging were imaged on a Leica Aperio Versa automated slide scanner (Leica Biosystems) at 20x magnification to a resolution of 0.325 µm/pixel utilizing a combination of five filter cubes at six wavelengths (405, 488, 546, 594, 647, 750 nm). Spinning disk confocal microscopy was performed using a Nikon Ti2 inverted light microscope with a Yokogawa CSU-X1 spinning disk head, an Andor DU-897 EMCCD camera, and four excitation LASERs (405, 488, 561, and 647nm), a Plan Apo Lambda 20x/0.75 NA air objective, and an Apo TIRF 100x/1.49NA oil immersion objective. High-magnification images were deconvolved (Richardson-Lucy deconvolution of image volumes, 20 iterations) using Nikon Elements software.

Image-based quantification of *C. muridarum*-infected surface epithelium

Quantification of *C. muridarum*-infected colonic epithelial surface was done utilizing QuPath. Proximal and distal regions of the colon were annotated by defining the proximal region as the portion containing proximal folds. The surface epithelia region was then annotated by manual drawing of the cells bordering the luminal space. Next, cells within the annotated surface epithelia region were defined using nuclei signal (Hoechst) with a radius expansion to allow for full inclusion of the cytoplasm for epithelial cells. Cells were scored positive or negative for *C. muridarum* infection by thresholding the staining

intensity for *Chlamydia* major outer membrane protein (MOMP) within the cytoplasm. Finally, the proportion of infected surface epithelial cells was quantified by dividing the percent of MOMP-positive cells by the total surface epithelial cells.

Histopathological Scoring

Inflammation and damage in H&E stained FFPE slides was quantified by a trained pathologist in a blinded fashion using methods adapted from Erben *et al.,* 2014 (23). A Colitis Score was calculated based on the sum of four subscores that each range from 0-4, with 0 as normal and 4 as most pronounced: lamina propria (LP) chronic inflammation, LP polymorphonuclear leukocytes (PMNs), depth of inflammation, and crypt inflammation.

***In vitro* bone marrow-derived macrophage (BMDM) stimulation**

Bone marrow was flushed from tibia and femur bones of wildtype, $Tnf^{\Delta reg/+}$, $Tnf^{\Delta ARE/+}$, and $Tnf^{\Delta reg/\Delta reg}$ mice in an aseptic environment. Cell clumps were disaggregated gently, and the cell suspension was centrifuged at 250xg for 5 min. The cell pellets were resuspended in DMEM medium (Gibco: 11995065) with 20% FBS, 100 IU/ml penicillin, 100 μg/ml streptomycin and 30% L cell-conditioned medium, and cultured at 37°C in 5% CO_2. For generation of M1 macrophages, BMDM were stimulated for 24 hr with 100 ng/mL of LPS and 20 ng/mL IFN-γ. After 6–7 days, nonadherent cells were aspirated and adherent macrophages were removed by washing plate with ice-cold PBS and scraping.

TNF enzyme-linked immunosorbent assay (ELISA)

To quantify TNF levels in stimulated BMDMs from wildtype, $Tnf^{\Delta reg/+}$, $Tnf^{\Delta ARE/+}$, and $Tnf^{\Delta reg/\Delta reg}$ mice, TNF ELISA was performed on stimulated BMDM lysates by adding equal volumes of each sample to individual wells of the Mouse TNF-alpha Quantikine ELISA Kit (R&D Systems) and following the manufacturer's protocol.

To quantify TNF levels in intestinal epithelial cells of CONV wildtype and $Tnf^{\Delta ARE/+}$ mice, small intestine was dissected and trisected (duodedum, jejunum, ileum) and colon was dissected and bisected (proximal colon and distal colon). Luminal content was flushed using PBS without calcium or magnesium. Next, the tissue was flayed open, washed twice in PBS without calcium and magnesium, and placed into cold chelation buffer consisting of 3 mM EDTA (Corning), 20 mM HEPES (Corning), and 0.5 mM DTT (Teknova) in PBS without calcium or magnesium for 1 hour at 4°C. Chelation buffer was discarded and colonic epithelium (crypts) were collected by vigorously shaking the colonic tissue in PBS. The shaking

process was repeated for a total of four times and the pooled crypt isolates were passed through a 100 µm filter. The dissociated crypts were instead lysed in NP-40 Lysis Buffer (RPI Corp.) with Protease and 1X Phosphatase Inhibitor Cocktail (Thermo Fisher Scientific) combined with brief sonication. Lysates were then quantified for total protein level using the Pierce BCA Protein Assay Kit (Thermo Fisher Scientific), diluted to a standard protein concentration, and TNF ELISA was performed by adding samples to individual wells of the Mouse TNF Quantikine ELISA Kit (R&D Systems) and following the manufacturer's protocol.

Fecal DNA testing for *C. muridarum* 23S rRNA

Fecal pellets were collected and frozen on dry ice immediately. Samples were thawed and DNA was extracted using the DNeasy PowerLyzer PowerSoil Kit (Qiagen) according to manufacturer's instructions. A region of *C. muridarum* 23S rRNA gene was amplified by PCR using HotStarTaq Plus Master Mix Kit (Qiagen) with the primers and thermocycling conditions developed by Mishkin *et al.,* 2022, which generated an expected product of 425 bp as determined by gel electrophoresis (6).

Quantification of tissue levels of kynurenine and tryptophan by liquid chromatography mass spectrometry

Tissue samples were weighed and homogenized by sonication (Fisher Sonic Dismembrator, 2 x 15 sec, power = 5, t = 0° C) to a final tissue density of 100 mg/mL in 1x PBS containing 10 % (*v/v*) MeOH. In separate Eppendorf tubes, 50 µL of tissue homogenates were spiked with 250 pmol of internal standard tryptophan-d_5 and mixed by gentle vortexing. The spiked homogenates were combined with 250 µL ACN/$CHCl_3$/MeOH (2:2:1) and 100 µL H_2O containing 100 mM $NaHCO_3$, vortexed vigorously, and centrifuged to facilitate clean phase separation (3,000 x *g*, 5 min, t = 5 °C). The upper aqueous layers were transferred to clean tubes, combined with 75 µL of ACN containing 25 mM dansyl chloride, and gently vortexed at room temperature for 15 min. Reactions were quenched with 10 µL of 5 % (*v/v*) HCOOH in H_2O, vortexed vigorously, and centrifuged to remove insoluble material (18,000 x *g*, 15 min, t = 5 °C). The clear supernatants were transferred to 2-mL autosampler vials equipped with low-volume polypropylene inserts and Teflon-lined rubber septa. Tryptophan and kynurenine were quantified as their dansyl derivatives by reverse phase LC-MS in the Vanderbilt Mass Spectrometry Core facility using a Waters Acquity UPLC system (Waters, Milford, MA) interfaced with Thermo TSQ Quantum Ultra AM mass spectrometer (Thermo Fisher Scientific, Waltham, MA). Multiple reaction monitoring (MRM) was performed with positive electrospray ionization (Torosyan et al., 2021 Cell Reports).

Cell lines and bacterial strains

C. muridarum strains CM001 (a gift from Catherine O'Connell(24)), CM001-GFP, and Cm-VU were propagated in Vero cells (CCL-81; ATCC). The Vero cells were maintained in Dulbecco's Modified Eagle Medium (DMEM; Gibco: 11995065) supplemented with 10% fetal bovine serum (FBS; Sigma-Aldrich). Confluent Vero cells were infected with *C. muridarum* by centrifugation at 1500 × g for 30 minutes at 10°C. *C. muridarum* strains were harvested by water lysis and sonication. Concentrations were determined using tittering protocols described previously (25).

Chlamydia **shuttle vector cloning and transformation**

The *Chlamydia* plasmid p2TK2$_{spec}$-Nigg-GFP was constructed by replacing *mCherry* in the p2TK2$_{spec}$-Nigg mCh(Gro$_{L2}$) vector (26) with *gfp*. The *gfp* gene was amplified from pGFP::Nigg (27) using the primers GFP_gro_inf_F (*Sac* I): 5'-AGCTTAAACgagctcATGAGTAAAGGAGAAGCACT-3' and GFP_gro_inf_R (*Kpn* I): 5'-ggtaccTTACTTGTATAGTTCATCCATGCCATGTG-3'. Additionally, the *groESL* promoter was amplified from p2TK2$_{spec}$-Nigg mCh(Gro$_{L2}$) using the primers groPro_inf_F (*Age* I): 5'-ACCGTATTACaccggtATTTTTAAAAATAGCAGTTG-3' and groPro_inf_R (*Sac* I): 5'-gagctcGTTTAAGCTCCTCGTATTTTTATATTCTA-3'. The *groESL* terminator was amplified from the same vector using the primers groTerm_inf_F (*Kpn* I): 5'-TACAAGTAAggtaccTTCCTCTAATGGGAACAAATAG-3' and groTerm_inf_R (*Not* I): 5'-TCCGTCGACgcggccgcAGAAAAGGATGGTCGTAA-3'. The vector p2TK2$_{spec}$-Nigg mCh(Gro$_{L2}$) was digested with *Age* I and *Not* I, and the three PCR products were combined and ligated into the digested vector using In-Fusion Cloning (Takara Bio). The resulting plasmid, p2TK2$_{spec}$-Nigg-GFP, was sequence verified by Sanger sequencing using the groPro_inf_F and the groTerm_inf_R primers at Eton Biosciences.

C. muridarum CM001 was transformed with p2TK2$_{spec}$-Nigg-GFP as follows: $1x10^7$ IFUs were incubated with 15 µg of plasmid DNA in buffer containing 0.9 mM calcium chloride for 40 minutes, then added to confluent Vero cells in a 6-well plate. Sixteen hours post-infection, 500 µg/ml of spectinomycin was added. Lysates from infected cells were used sequentially to infect new monolayers every 48 hours until GFP-positive inclusions were visible. Transformants were plaque-purified to obtain a clonal isolate.

Cm-VU isolation

Whole colons were isolated from *C. muridarum*-positive *Tnf*$^{\Delta ARE/+}$ mice from the CONV facility and placed in ice-cold 1X SPG (sucrose-phosphate-glutamate) buffer (219 mM sucrose, 3.7 mM KH_2PO_4, 4.9 mM L-glutamic acid in cell-grade water to pH 7.4-7.6 using NaOH). All steps were performed on ice or at 4°C. After flushing out fecal material with DPBS (Gibco), the colon was filleted, minced into 2 mm pieces, and washed with DPBS. Epithelial cells were isolated by incubating the tissue in 8 mM EDTA-DPBS for 75 minutes with rotation, followed by three DPBS washes. Cells were further dislodged by vigorous shaking in 0.1% BSA-DPBS, and the supernatant was filtered through a 70 µm filter. The filtrate was centrifuged at 300 ×g for 5 minutes, and the pellet was resuspended in water, sonicated, and filtered through a 0.45 µm filter. The *Chlamydi*a-containing filtrate was added to Vero cells. After 18 hours, 0.5 µg/ml cycloheximide and 50 µg/ml gentamicin were added. Bacteria were passaged as necessary.

Whole genome sequencing (WGS) of *C. muridarum*

C. muridarum isolates Cm-VU and CM001 were prepared from six 6-well plates of Vero cells infected at an MOI of 2 and grown for 48 hours. Cells were lysed in sterile water, sonicated, and centrifuged at 500 ×g for 5 minutes at 4°C to remove host cell debris. The supernatant was then centrifuged at 19,000 ×g for 5 minutes at 4°C to pellet *Chlamydia* elementary bodies (EBs), which were treated with 8 U of DNase I (New England Biolabs) in 1x DNase I buffer (New England Biolabs) for 1 hour at 37°C to degrade host DNA. EBs were washed in DPBS, and genomic DNA was extracted using the DNeasy Blood and Tissue Kit (Qiagen), following the manufacturer's protocol, quantified with the Qubit dsDNA Quantification Assay Kit (Invitrogen), and assessed for the quality of high molecular weight DNA by agarose gel electrophoresis.

Cm-VU was sequenced by Plasmidsaurus (https://www.plasmidsaurus.com) using their Hybrid Bacterial Genome workflow, combining Oxford Nanopore and Illumina sequencing. An amplification-free library was prepared using the Oxford Nanopore Rapid Barcoding Kit 96 V14 (Cat# SQK-RBK114.96) and sequenced on a PromethION P24 instrument with a R10.4.1 flow cell. Basecalling was performed in super-accurate mode using ont-doradod-for promethion v7.1.4, with a minimum Qscore of 10, and adapters were trimmed using MinKnow. The Illumina library was prepared using the SeqWell ExpressPlex 96 kit (Cat# 301098) and sequenced on an Illumina NextSeq2000 instrument with paired-end 2x150bp reads. Nanopore fastq reads were first processed using filtlong (v0.2.1), retaining reads > 1000 bp, and subsampled to 100x coverage using rasusa (v2.0.0) (28). Assembly was performed with Flye (v2.9.4) (29, 30). The assembly was polished using Polypolish (v0.6.0) (31, 32). with Illumina reads, resulting in a 1,072,053 bp chromosome and 7,501 bp plasmid.

C. muridarum CM001 was sequenced by the Microbial Sequencing and Analysis Center, now SeqCenter in Pittsburgh, PA (https://www.seqcenter.com). The library was prepared using the Illumina DNA Prep kit with IDT 10bp UDI indices and sequenced on an Illumina NextSeq 2000, producing 2x151bp reads. Demultiplexing and adapter trimming was preformed using bcl-convert (v3.9.3) (33). The *C. muridarum* CM001 genome was assembled in Geneious Prime (v2022.1). Fastq reads were trimmed using BBDuk, normalized using BBNorm, paired reads were merged with BBMerge, and duplicate reads were removed using Dedupe. The processed reads were aligned to the published CM001 reference genome (NZ_CP027217) (24) using Bowtie2, and the resulting consensus sequence was used for pangenomic analysis.

Pangenomic analysis

Chlamydia genomes (n=13) and one *Chlamydiifrater phoenicopteri* genome were retrieved from NCBI (accession numbers tablulated below). A comparison of 15 *Chlamydia* genomes including Cm-VU and CM001 was performed using anvi'o version 7.1 (34). To satisfy anvi'o formatting requirements, the fasta files containing all genomes were converted into contigs-fasta format using anvi-script-reformat-fasta with parameters --simplify_names and --seq_type NT. The reformatted genomes were then used to generate databases using the command anvi-gen-contigs-database. These databases were annotated using anvi-run-hmms and anvi-run-cogs (35). and used to generate a genomes storage database using anvi-gen-genomes-storage. The pangenome was constructed using anvi-pan-genome with parameters --minbit 0.5, --mcl-inflation 10, and --use-ncbi-blast. Average nucleotide identity between all pairs of genomes was calculated using anvi-compute-genome-similarity using --pyANI program (36) after including *C. phoenicopteri* as an outgroup. ANI calculations were run once each using the ANIb and TETRA alignment methods. The pangenome figure was finalized using Inkscape (37). The ANI dendrogram was visualized using the Interactive Tree of Life (38).

NCBI accession numbers for species and strains used in pangenomic analyses

**Species**	**Strain**	**NCBI Accession**
C. trachomatis	A/HAR-13	GCA_000012125.1
C. trachomatis	C/TW-3	GCF_000507225.1
C. trachomatis	D/UW-3/CX	GCA_000008725.1
C. trachomatis	E/Bour	GCF_000318645.1
C. trachomatis	L1/440/LN	GCF_000318825.1
C. trachomatis	L2/434/Bu	GCA_000068585.1
C. muridarum	Weiss	GCF_000174995.1

C. muridarum	Nigg	GCA_000006685.1
C. abortus	S26/3	GCF_000026025.1
C. psittaci	6BC	GCF_000204255.1
C. pecorum	E58	GCF_000204135.1
C. suis	S45	GCF_019405085.1
C. pneumoniae	CWL029	GCF_000008745.1
Chlamydiifrater phoenicopteri	14-27711_R47	GCF_902807005.1

Genomic sequencing and comparison of native *C. muridarum* strain (Cm-VU)

Genome comparisons between reference *Chlamydia* genomes and the *Chlamydia* isolate Cm-VU were conducted using average nucleotide identity (ANI) and tetranucleotide frequency correlation coefficient (TETRA) analyses, with species-level cutoffs set at >95% for ANI and >0.989 for TETRA (39–41). The genome of the Cm-VU isolate was sequenced and compared to 14 other *Chlamydia* genomes, including 6 *C. trachomatis* strains, 3 *C. muridarum* strains (including CM001, which was used for experiments in this study), and reference genomes from 5 other *Chlamydia* species. All genomes, except for CM001 and Cm-VU, were acquired from publicly available sequences on NCBI (tabulated above).

SUPPLEMENTAL REFERENCES

1. Li J, et al. Identification and multimodal characterization of a specialized epithelial cell type associated with Crohn's disease. *Nature Communications 2024 15:1*. 2024;15(1):1–19.
2. Kontoyiannis D, et al. Impaired On/Off Regulation of TNF Biosynthesis in Mice Lacking TNF AU-Rich Elements: Implications for Joint and Gut-Associated Immunopathologies. *Immunity*. 1999;10(3):387–398.
3. Roulis M, et al. Intestinal epithelial cells as producers but not targets of chronic TNF suffice to cause murine Crohn-like pathology. *Proc Natl Acad Sci U S A*. 2011;108(13):5396–5401.
4. Burger E, et al. Loss of Paneth Cell Autophagy Causes Acute Susceptibility to Toxoplasma gondii-Mediated Inflammation. *Cell Host Microbe*. 2018;23(2):177-190.e4.
5. Powell AE, et al. The pan-ErbB negative regulator lrig1 is an intestinal stem cell marker that functions as a tumor suppressor. *Cell*. 2012;149(1):146–158.
6. Mishkin N, et al. Reemergence of the Murine Bacterial Pathogen Chlamydia muridarum in Research Mouse Colonies. *Comp Med*. 2022;72(4):230–242.
7. Butler A, et al. Integrating single-cell transcriptomic data across different conditions, technologies, and species. *Nature Biotechnology 2018 36:5*. 2018;36(5):411–420.
8. Wolf FA, Angerer P, Theis FJ. SCANPY: Large-scale single-cell gene expression data analysis. *Genome Biol*. 2018;19(1):1–5.
9. Borry M. Sourcepredict: Prediction of metagenomic sample sources using dimension reduction followed by machine learning classification. *J Open Source Softw*. 2019;4(41):1540.
10. Minot SS, Krumm N, Greenfield NB. One Codex: A Sensitive and Accurate Data Platform for Genomic Microbial Identification. *bioRxiv*. 2015;027607.
11. Bankhead P, et al. QuPath: Open source software for digital pathology image analysis. *Scientific Reports 2017 7:1*. 2017;7(1):1–7.
12. Harris PA, et al. Research electronic data capture (REDCap)--a metadata-driven methodology and workflow process for providing translational research informatics support. *J Biomed Inform*. 2009;42(2):377–381.
13. Harris PA, et al. The REDCap consortium: Building an international community of software platform partners. *J Biomed Inform*. 2019;95:103208.

14. Cheng AC, et al. REDCap on FHIR: Clinical Data Interoperability Services. *J Biomed Inform*. 2021;121. https://doi.org/10.1016/J.JBI.2021.103871.
15. Lawrence CE, et al. A REDCap-based model for electronic consent (eConsent): Moving toward a more personalized consent. *J Clin Transl Sci*. 2020;4(4):345–353.
16. Sigmon JS, et al. Content and Performance of the MiniMUGA Genotyping Array: A New Tool To Improve Rigor and Reproducibility in Mouse Research. *Genetics*. 2020;216(4):905–930.
17. Banerjee A, et al. Succinate Produced by Intestinal Microbes Promotes Specification of Tuft Cells to Suppress Ileal Inflammation. *Gastroenterology*. 2020;159(6):2101-2115.e5.
18. Chen B, et al. Differential pre-malignant programs and microenvironment chart distinct paths to malignancy in human colorectal polyps. *Cell*. 2021;184(26):6262-6280.e26.
19. Petukhov V, et al. dropEst: pipeline for accurate estimation of molecular counts in droplet-based single-cell RNA-seq experiments. *Genome Biol*. 2018;19(1):78.
20. Vega PN, et al. Cancer-Associated Fibroblasts and Squamous Epithelial Cells Constitute a Unique Microenvironment in a Mouse Model of Inflammation-Induced Colon Cancer. *Front Oncol*. 2022;12. https://doi.org/10.3389/FONC.2022.878920.
21. Elizarraras JM, et al. WebGestalt 2024: faster gene set analysis and new support for metabolomics and multi-omics. *Nucleic Acids Res*. 2024;52(W1):W415–W421.
22. Choi HMT, et al. Third-generation in situ hybridization chain reaction: Multiplexed, quantitative, sensitive, versatile, robust. *Development (Cambridge)*. 2018;145(12). https://doi.org/10.1242/DEV.165753/48466.
23. Erben U, et al. A guide to histomorphological evaluation of intestinal inflammation in mouse models. *Int J Clin Exp Pathol*. 2014;7(8):4557.
24. Poston TB, et al. T Cell-Independent Gamma Interferon and B Cells Cooperate To Prevent Mortality Associated with Disseminated Chlamydia muridarum Genital Tract Infection. *Infect Immun*. 2018;86(7). https://doi.org/10.1128/IAI.00143-18.
25. Dolat L, et al. Chlamydia repurposes the actin-binding protein EPS8 to disassemble epithelial tight junctions and promote infection. *Cell Host Microbe*. 2022;30(12):1685.
26. Cortina ME, et al. The inclusion membrane protein IncS is critical for initiation of the Chlamydia intracellular developmental cycle. *PLoS Pathog*. 2022;18(9). https://doi.org/10.1371/JOURNAL.PPAT.1010818.
27. Skilton RJ, et al. The Chlamydia muridarum plasmid revisited : new insights into growth kinetics. *Wellcome Open Res*. 2018;3:25.
28. Hall MB. Rasusa: Randomly subsample sequencing reads to a specified coverage. *J Open Source Softw*. 2022;7(69):3941.
29. Kolmogorov M, et al. Assembly of long, error-prone reads using repeat graphs. *Nature Biotechnology 2019 37:5*. 2019;37(5):540–546.
30. Lin Y, et al. Assembly of long error-prone reads using de Bruijn graphs. *Proc Natl Acad Sci U S A*. 2016;113(52):E8396–E8405.
31. Wick RR, Holt KE. Polypolish: Short-read polishing of long-read bacterial genome assemblies. *PLoS Comput Biol*. 2022;18(1). https://doi.org/10.1371/JOURNAL.PCBI.1009802.
32. Bouras G, et al. How low can you go? Short-read polishing of Oxford Nanopore bacterial genome assemblies. *Microb Genom*. 2024;10(6):001254.
33. BCL Convert [Internet]. https://support-docs.illumina.com/SW/BCL_Convert/Content/SW/FrontPages/BCL_Convert.htm. Accessed August 15, 2024.
34. Eren AM, et al. Community-led, integrated, reproducible multi-omics with anvi'o. *Nat Microbiol*. 2021;6(1):3–6.
35. Eddy SR. Accelerated Profile HMM Searches. *PLoS Comput Biol*. 2011;7(10):e1002195.
36. Pritchard L, et al. Genomics and taxonomy in diagnostics for food security: soft-rotting enterobacterial plant pathogens. *Analytical Methods*. 2015;8(1):12–24.
37. IW D. Inkscape - Draw Freely. [Internet]. https://inkscape.org/. Accessed August 15, 2024.
38. Letunic I, Bork P. Interactive Tree Of Life (iTOL) v5: an online tool for phylogenetic tree display and annotation. *Nucleic Acids Res*. 2021;49(W1):W293–W296.
39. Richter M, Rosselló-Móra R. Shifting the genomic gold standard for the prokaryotic species definition. *Proc Natl Acad Sci U S A*. 2009;106(45):19126–19131.
40. Goris J, et al. DNA-DNA hybridization values and their relationship to whole-genome sequence similarities. *Int J Syst Evol Microbiol*. 2007;57(1):81–91.
41. Pillonel T, et al. Taxogenomics of the order Chlamydiales. *Int J Syst Evol Microbiol*. 2015;65(Pt 4):1381–1393.

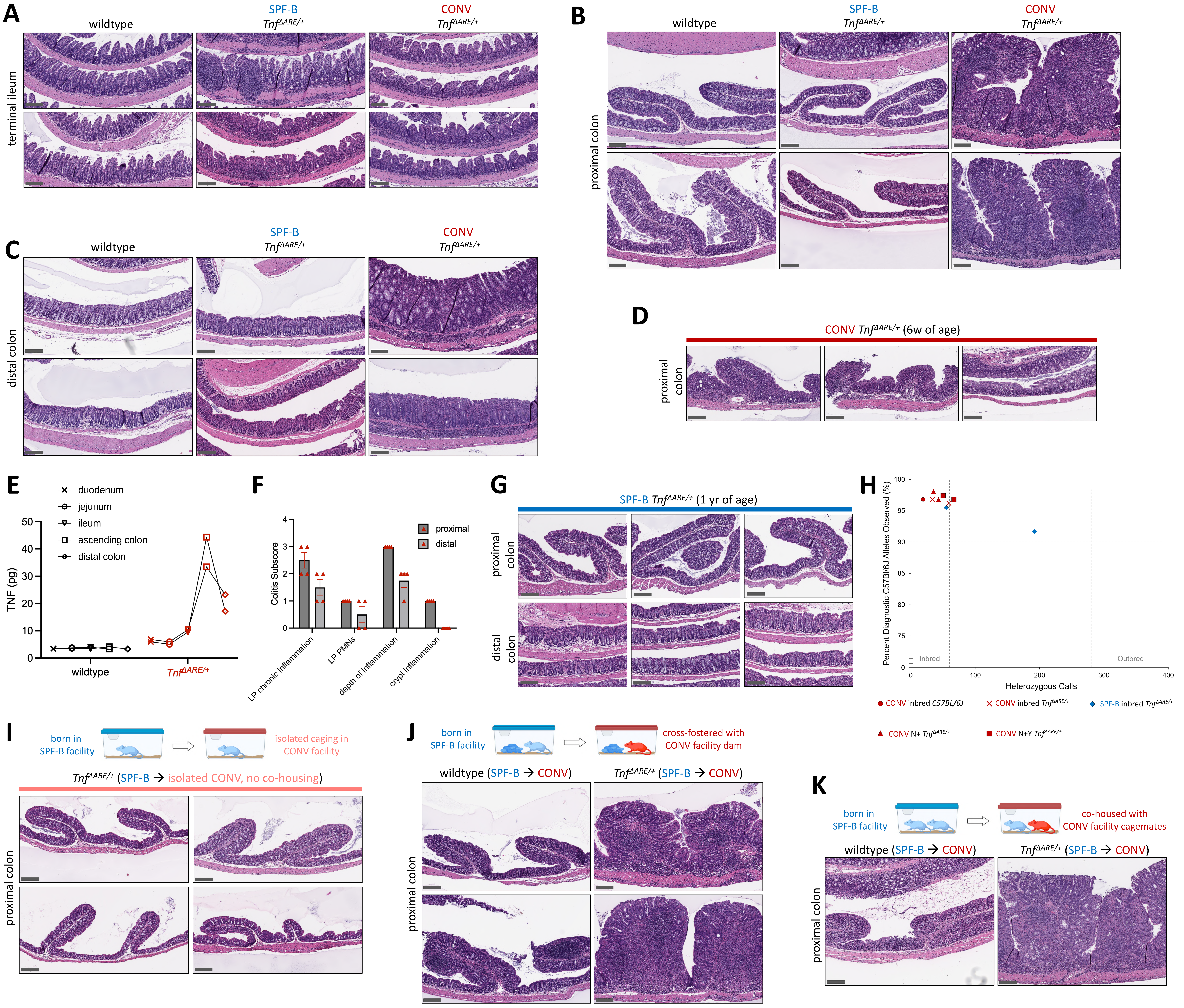

Figure S1. Genetic background and environmental factors are insufficient to drive colonic inflammation in the *Tnf*$^{\Delta ARE/+}$ model of Crohn's-like disease.
(A - C) Additional replicates of H&E-stained intestinal sections (terminal ileum **(A)**, proximal colon **(B)**, distal colon **(C)**) from wildtype (N = 3) and *Tnf*$^{\Delta ARE/+}$ mice from SPF-B (N = 4) and CONV facilities (N = 5). Wildtype samples are from the CONV facility and all mice are age-matched (34-42 w of age). **(D)** H&E-stained proximal colon sections from young (6-7 w) CONV *Tnf*$^{\Delta ARE/+}$ mice (N = 3). **(E)** TNF protein levels measured by ELISA in each region of the small intestine and colon from CONV wildtype (N = 2) and CONV *Tnf*$^{\Delta ARE/+}$ (N = 2) mice, aged 26-38w. **(F)** Colitis subscores that contribute to overall colitis score from histopathological scoring of colons from age-matched CONV *Tnf*$^{\Delta ARE/+}$ mice (N = 4, 12w of age), separated by proximal and distal colon regions. Mean plus SEM are shown, and statistical significance was not determined. LP = lamina propria. PMNs = polymorphonuclear leukocytes. **(G)** H&E-stained colonic sections from 1 year-old SPF-B *Tnf*$^{\Delta ARE/+}$ mice (N = 3). **(H)** Comparison of background amongst SPF-B and CONV facility mice from various indicated generations of backcrossing and inbreeding using MiniMUGA genetic analysis. "N+" indicates more than one backcross to C57BL/6J and "N+Y" indicates the more than one backcross to C57BL/6J, one of which was male to refresh the Y chromosome. **(I)** H&E-stained proximal colon sections from SPF-B *Tnf*$^{\Delta ARE/+}$ mice (N = 4, 27w of age at collection) transferred as adults and housed in isolated caging in the CONV facility. **(J)** Additional replicates of H&E-stained proximal colon sections from SPF-B wildtype *Tnf*$^{\Delta ARE/+}$ mice transferred and co-housed/fostered as pups in the CONV facility with a wildtype *or Tnf*$^{\Delta ARE/+}$ foster dam along with the dam's age-matched biological pups. Pups were transferred and fostered within the first 3 days of birth and co-housed until experimental collection (37w of age, N = 3 wildtype, N = 4 Tnf$^{\Delta ARE/+}$). **(K)** Additional replicates of H&E-stained proximal colon sections from SPF-B adult mice (N = 2 wildtype, N = 3 Tnf$^{\Delta ARE/+}$) transferred and co-housed in the CONV facility in mixed-sex conditions until experimental collection (32-54w of age). CONV donors are of either wildtype or *Tnf*$^{\Delta ARE/+}$ genotype. Scale bars = 200 μm.
Related to Figure 1.

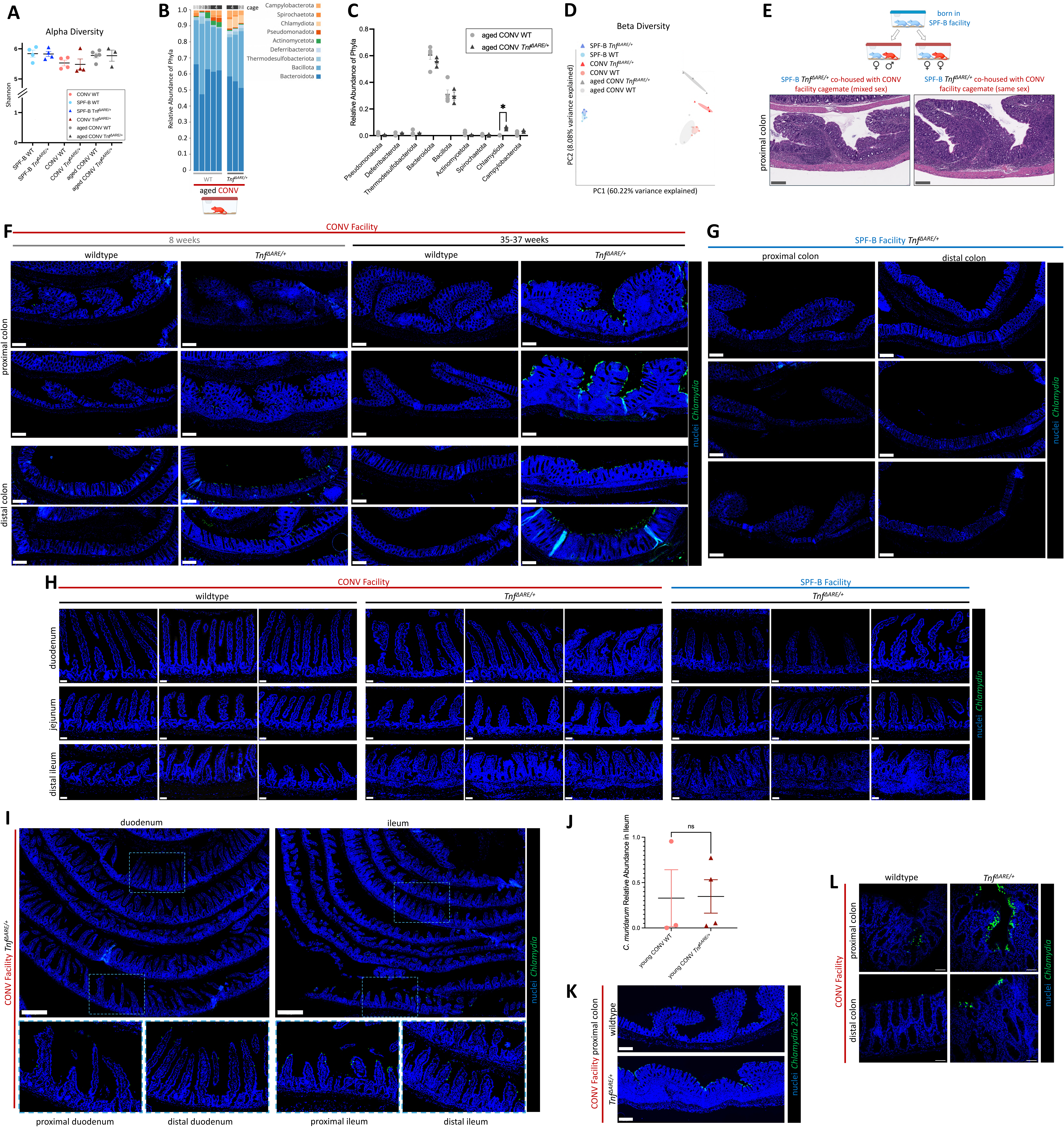

Figure S2. Shotgun metagenomic sequencing and *in situ* imaging of *Chlamydia muridarum* in wildtype and *Tnf*$^{\Delta ARE/+}$ intestinal specimens.

(A) Alpha diversity, measured as the Shannon index, of individual proximal colon luminal content shotgun metagenomic datasets at the species level (filtered for Eubacteria kingdom only). N = 4 per condition. Mean plus standard error of the mean (SEM) are shown and statistical significance was determined using ordinary one-way ANOVA with multiple comparisons. **(B-C)** Shotgun metagenomic data of proximal colon luminal contents from aged (20-24w of age) wildtype (N = 4) and *Tnf*$^{\Delta ARE/+}$ (N = 3) mice from the CONV facility from 4 cages. Data is represented as relative abundance of mapped phyla for individual wildtype and *Tnf*$^{\Delta ARE/+}$ mice in the CONV facility. Mean plus standard error of the mean (SEM) are shown and statistical significance was determined using multiple unpaired t tests with false discovery rate (FDR) of 1%. **(D)** Beta diversity at the species level amongst the same samples. N = 4 per condition. **(E)** Representative H&E-stained proximal colon sections from SPF-B adult *Tnf*$^{\Delta ARE/+}$ mice (N = 2 same-sex, N = 4 mixed-sex) transferred and co-housed in the CONV facility in same-sex or mixed-sex conditions until experimental endpoint (15-24w of age). Scale bars = 200 µm. **(F)** Additional replicates of IF images of *Chlamydia* major outer membrane protein (MOMP - green) and nuclei (Hoechst - blue) co-staining on colonic sections from wildtype (N = 3 at 8w of age, N = 4 at 35-37w of age) and *Tnf*$^{\Delta ARE/+}$ (N = 3 at 8w of age, N = 3 at 35-37w of age) mice from the CONV facility. Scale bars = 200 µm. **(G)** IF images of *Chlamydia* major outer membrane protein (MOMP - green) and nuclei (Hoechst - blue) co-staining on colonic sections from aged *Tnf*$^{\Delta ARE/+}$ (N = 3 at 35-37w of age) mice from the SPF-B facility. Scale bars = 200 µm. **(H)** IF images of *Chlamydia* major outer membrane protein (MOMP - green) and nuclei (Hoechst - blue) co-staining in small intestinal sections. Representative images from duodenum, jejunum, and terminal/distal ileum regions of CONV wildtype (N = 3), CONV *Tnf*$^{\Delta ARE/+}$ (N = 3), and SPF-B *Tnf*$^{\Delta ARE/+}$ (N = 3) mice. All mice are 35-37w of age. Scale bars = 50 µm. **(I)** IF images of *Chlamydia* major outer membrane protein (MOMP - green) and nuclei (Hoechst - blue) co-staining in small intestinal sections (duodenum – left, ileum – right) of a rare case of small intestinal *Chlamydia* inclusions in a young (8w) *Tnf*$^{\Delta ARE/+}$ mouse . Scale bars = 400 µm. **(J)** Relative abundance of *C. muridarum* from shotgun metagenomic sequencing data of terminal ileum luminal contents from young (6-7w of age) wildtype (N = 3) and *Tnf*$^{\Delta ARE/+}$ (N = 4) mice from the CONV facility. Mean plus standard error of the mean (SEM) are shown and statistical significance was determined using an unpaired t test. **(K)** Representative images of fluorescence *in situ* hybridization of *C. muridarum* 23S RNA (green) with nuclei (Hoechst – blue) co-staining in age-matched (13w) wildtype and *Tnf*$^{\Delta ARE/+}$ mice from the CONV facility. Scale bars = 200 µm. **(L)** Representative IF confocal microscopy images of *Chlamydia* major outer membrane protein (MOMP - green) and nuclei (Hoechst - blue) co-staining of the proximal and distal colon. N = 3 per condition. Scale bars = 50 µm. p-value * < 0.05, ** < 0.01, *** < 0.001. **** < 0.0001.

Related to Figure 2 and Table S1.

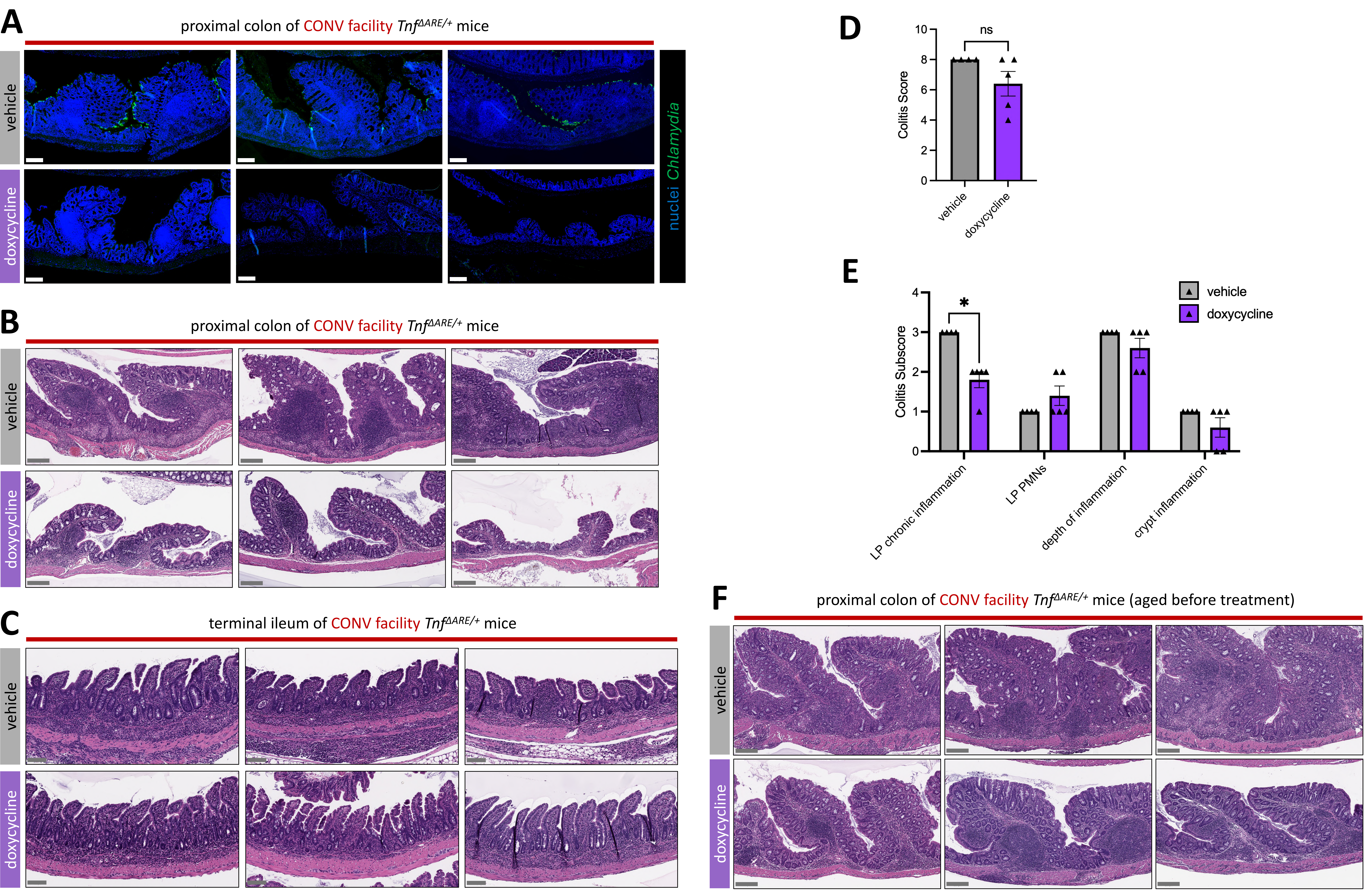

Figure S3. Doxycycline treatment clears *Chlamydia muridarum* infection and resolves inflammation in the colon of *$Tnf^{\Delta ARE/+}$* mice. **(A)** Additional replicates of IF images of *Chlamydia* major outer membrane protein (MOMP - green) and nuclei (Hoechst - blue) co-staining on proximal colon sections from CONV *$Tnf^{\Delta ARE/+}$* mice treated with doxycycline or vehicle. N = 4 mice per condition, age-matched at 11-12w of age at harvest. Scale bars = 200 µm. **(B)** Additional replicates of H&E-stained proximal colon sections from from CONV *$Tnf^{\Delta ARE/+}$* mice treated with doxycycline or vehicle. N = 4 mice per condition, age-matched at 11-12w of age at harvest. Scale bars = 200 µm. **(C)** H&E-stained terminal ileum sections from from CONV *$Tnf^{\Delta ARE/+}$* mice treated with doxycycline or vehicle. N = 4 mice per condition, age-matched at 11-12w of age at harvest. Scale bars = 100 µm. **(D)** Colitis scores from histopathological scoring of colons from aged CONV *$Tnf^{\Delta ARE/+}$* mice (13-16w at start, 18-23w at harvest) treated with doxycycline or vehicle. N = 4 mice per condition. Mean plus SEM are shown, and statistical significance was determined using an unpaired t test. **(E)** Colitis subscores that contribute to overall colitis score in D. Mean plus SEM are shown, and statistical significance was determined using multiple unpaired t tests with FDR of 1%. LP = lamina propria. PMNs = polymorphonuclear leukocytes. **(F)** H&E-stained proximal colon sections from aged CONV *$Tnf^{\Delta ARE/+}$* mice (13-16w at start, 18-23w at harvest) treated with doxycycline or vehicle. N = 4 mice per condition. Scale bars = 200 µm. p-value * < 0.05, ** < 0.01, *** < 0.001. **** < 0.0001. **Related to Figure 3.**

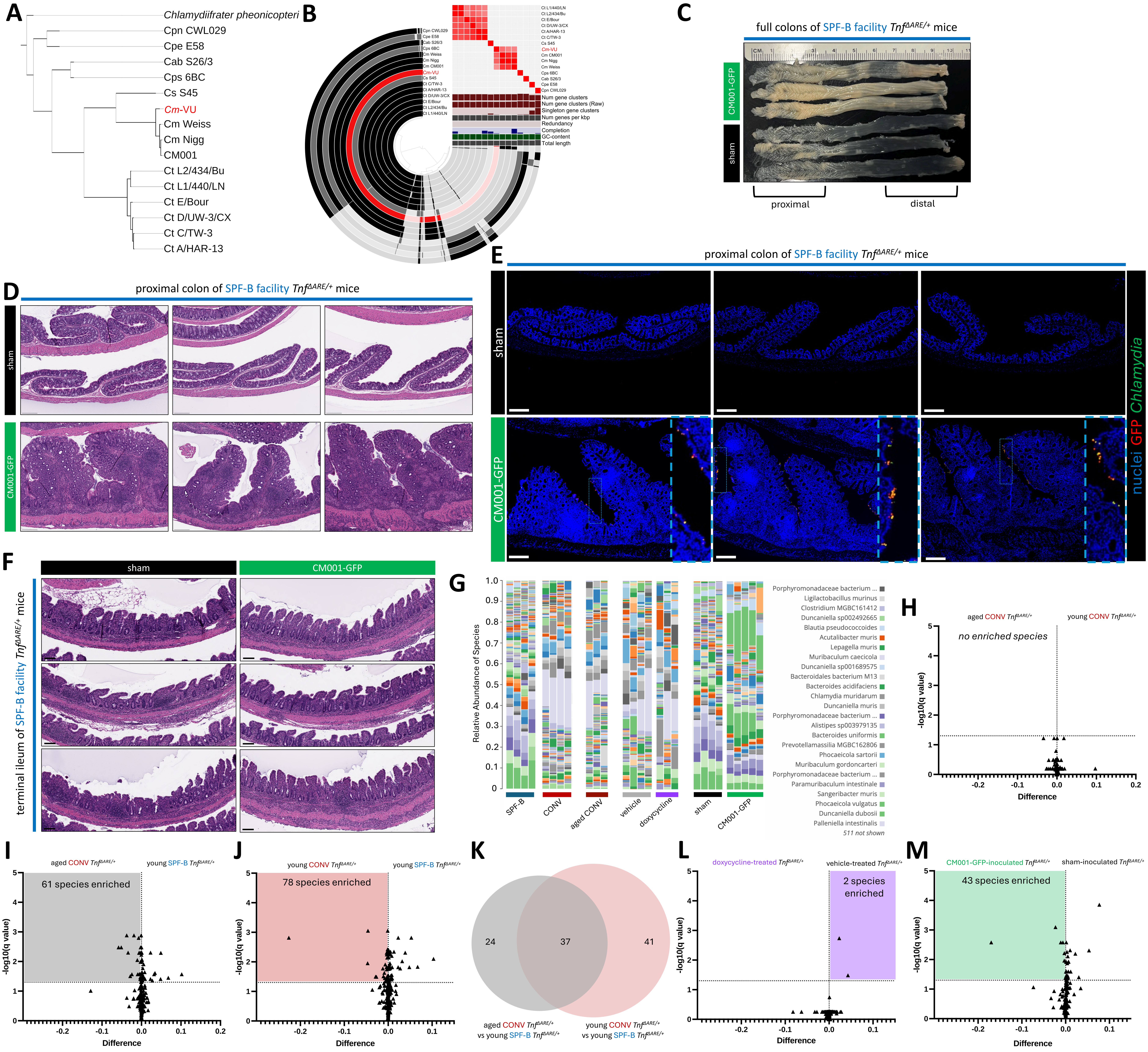

Figure S4. *Chlamydia muridarum* strain CM001-GFP is sufficient to drive colonic inflammation in *Tnf*$^{\Delta ARE/+}$ mice.

(A) Pairwise analysis of genome similarity identifies *Cm*-VU as *C. muridarum*. ANI percentage identity was calculated using pyANI within anvi'o and the resulting dendrogram was visualized to group *Chlamydia* genomes. *Chlamydiifrater phoenicopterid* was used as an outgroup. Notably, *Cm*-VU (red) is grouped with the *C. muridarum* genomes. **(B)** Pangenomic analysis of *Chlamydia* genomes. The phylogram is clustered based on full ANI percentage identity, alternating species are colored in black and grey, and *Cm*-VU is highlighted in red. The heatmap (top-right) displays ANI similarity between genomes. Shades of red denote pairwise ANI of 95% or greater. **(C)** Macroscopic images of whole colons from *Tnf*$^{\Delta ARE/+}$ mice that are sham or CM001-GFP-inoculated. 3 representative colons from N = 10 mice per condition, age-matched at 16-20w of age at harvest. **(D)** Additional replicates of H&E-stained proximal colon sections from *Tnf*$^{\Delta ARE/+}$ mice that are sham or CM001-GFP-inoculated. N = 10 mice per condition, age-matched at 16-20w of age at harvest. Scale bars = 200 μm. **(E)** Additional replicates of IF images of *Chlamydia* major outer membrane protein (MOMP - green), endogenous GFP from CM001-GFP (GFP – red), and nuclei (Hoechst - blue) co-staining on proximal colon sections from *Tnf*$^{\Delta ARE/+}$ mice that are sham or CM001-GFP-inoculated. N = 5 mice per condition, age-matched at 16-20w of age at harvest. Inset to show colocalization of GFP and MOMP signal in CM001-GFP-inoculated conditions. Scale bars = 200 μm. **(F)** Representative images of H&E-stained terminal ileal sections from *Tnf*$^{\Delta ARE/+}$ mice that are sham or CM001-GFP-inoculated. N = 10 mice per condition, age-matched at 16-20w of age at harvest. Scale bars = 100 μm. **(G)** Shotgun metagenomic data, filtered for only the eubacteria kingdom and represented as relative abundance at the species level, of proximal colon luminal contents from individual *Tnf*$^{\Delta ARE/+}$ mice in the experimental paradigms in Figure 2A (young CONV facility), Supp 2C (aged CONV facility), Figure 3G (vehicle vs doxycycline-treated), and Figure 3P (sham vs CM001-GFP-inoculated). Highest abundance species names are shown. **(H)** Statistically enriched species in aged CONV (20-24w of age) vs young CONV (6-7 w of age) *Tnf*$^{\Delta ARE/+}$ mice derived from shotgun metagenomic data. Statistical enrichment was determined using multiple unpaired t tests with FDR of 5%, which resulted in 0 hits. **(I)** Statistically enriched species in aged CONV (20-24w of age) vs young SPF-B (6-7 w of age) *Tnf*$^{\Delta ARE/+}$ mice derived from shotgun metagenomic data. Statistical enrichment was determined using multiple unpaired t tests with FDR of 5%, which resulted in 61 hits. **(J)** Statistically enriched species in young CONV (6-7w of age) vs young SPF-B (6-7 w of age) *Tnf*$^{\Delta ARE/+}$ mice derived from shotgun metagenomic data. Statistical enrichment was determined using multiple unpaired t tests with FDR of 5%, which resulted in 78 hits. **(K)** Venn diagram depicting enriched species that are shared between young CONV and aged CONV *Tnf*$^{\Delta ARE/+}$ mice when compared to young SPF-B mice. **(L)** Statistically enriched species in doxycycline-treated vs vehicle-treated *Tnf*$^{\Delta ARE/+}$ mice from the CONV facility of mixed ages derived from shotgun metagenomic data. Statistical enrichment was determined using multiple unpaired t tests with FDR of 5%, which resulted in 2 hits. **(M)** Statistically enriched species in CM001-GFP-inoculated vs sham-inoculated *Tnf*$^{\Delta ARE/+}$ mice from the SPF-B facility of mixed ages derived from shotgun metagenomic data. Statistical enrichment was determined using multiple unpaired t tests with FDR of 5%, which resulted in 43 hits. p-value * < 0.05, ** < 0.01, *** < 0.001. **** < 0.0001.

Related to Figure 3, Table S1, Table S2, and Table S3.

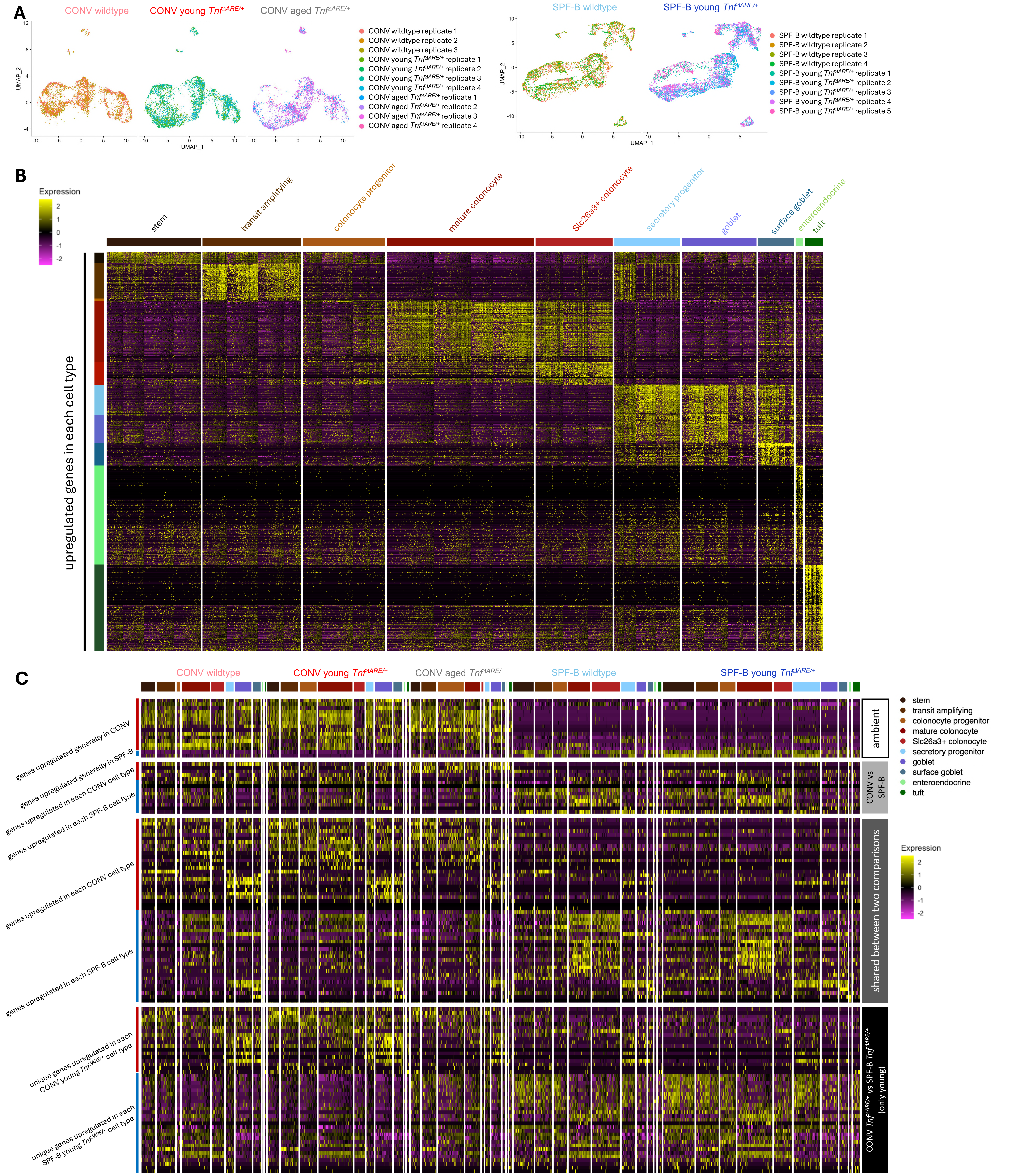

Figure S5. *Chlamydia* induces host defense-related transcriptional changes in proximal colonic epithelial cell types.
(A) UMAP co-embedding of scRNA-seq with sample replicate overlay indicated by color. **(B)** Heatmap of cell type specific differential gene expression from scRNA-seq data, pooling wildtype and $Tnf^{\Delta ARE/+}$ samples, organized by cell type on x-axis. Genes on y-axis are organized by upregulated genes for each cell type, identified by differential expression analysis of each cell type compared to the rest of the cells. Differentially expressed genes were defined as those with log fold change > 1. **(C)** Heatmap of differentially expressed genes from scRNA-seq data, split by cell type and sample type. Genes on the y-axis are organized by generally upregulated (ambient genes) in each facility and those that are differentially expressed in the comparison of CONV vs SPF-B samples from scRNA-seq data (pooled wildtype and $Tnf^{\Delta ARE/+}$ samples), differentially expressed in the comparison of CONV young $Tnf^{\Delta ARE/+}$ vs SPF-B young $Tnf^{\Delta ARE/+}$, and those that are shared between the comparisons. Differentially expressed genes were defined as those with log fold change > 1.5. Generally upregulated genes for each facility were those that were differentially expressed in more than 4 cell types.
Related to Figure 4, Table S4, Table S5, Table S6, and Table S7.

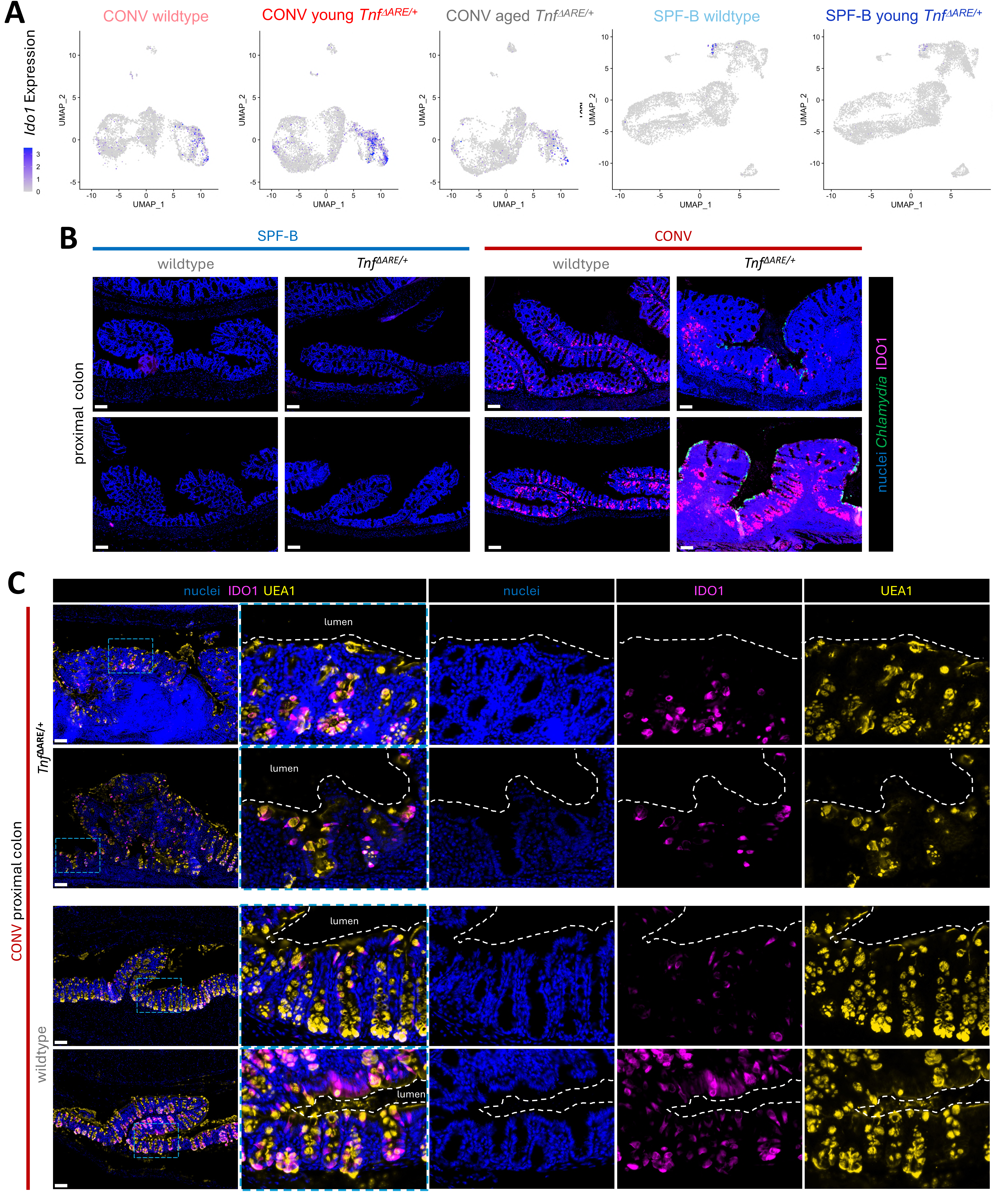

Figure S6. IDO1 is highly expressed in PC goblet cells of *Chlamydia*-colonized wildtype and *Tnf*$^{\Delta ARE/+}$ mice.
(A) UMAP co-embedding of scRNA-seq, separated into individual maps for each sample type, with overlay of *Ido1* gene expression indicated by the color gradient. **(B)** Additional replicates of IF images of IDO1 (magenta), *Chlamydia* major outer membrane protein (green), and nuclei (Hoechst - blue) co-staining on proximal colon sections from wildtype and *Tnf*$^{\Delta ARE/+}$ mice from the SPF-B and CONV facilities. N = 3 mice, age-matched at 34-42w of age at harvest. Scale bars = 100 µm. Data in first row, second column image first appears as nuclei/MOMP co-staining (no IDO1) in Figure S2G. Data in fourth column images first appear as nuclei/MOMP co-staining (no IDO1) in Figure S2F. **(C)** Additional replicates of IF images of IDO1 (magenta), UEA1 lectin (yellow), and nuclei (Hoechst - blue) co-staining on proximal colon sections from wildtype and *Tnf*$^{\Delta ARE/+}$ mice from the CONV facilty. Inset image to show colocalization of UEA1 lectin, a goblet and secretory granule marker, with IDO1. N = 3 mice, age-matched at 16-17w of age at harvest. Scale bars = 100 µm.
Related to Figure 4, Table S5, and Table S6.

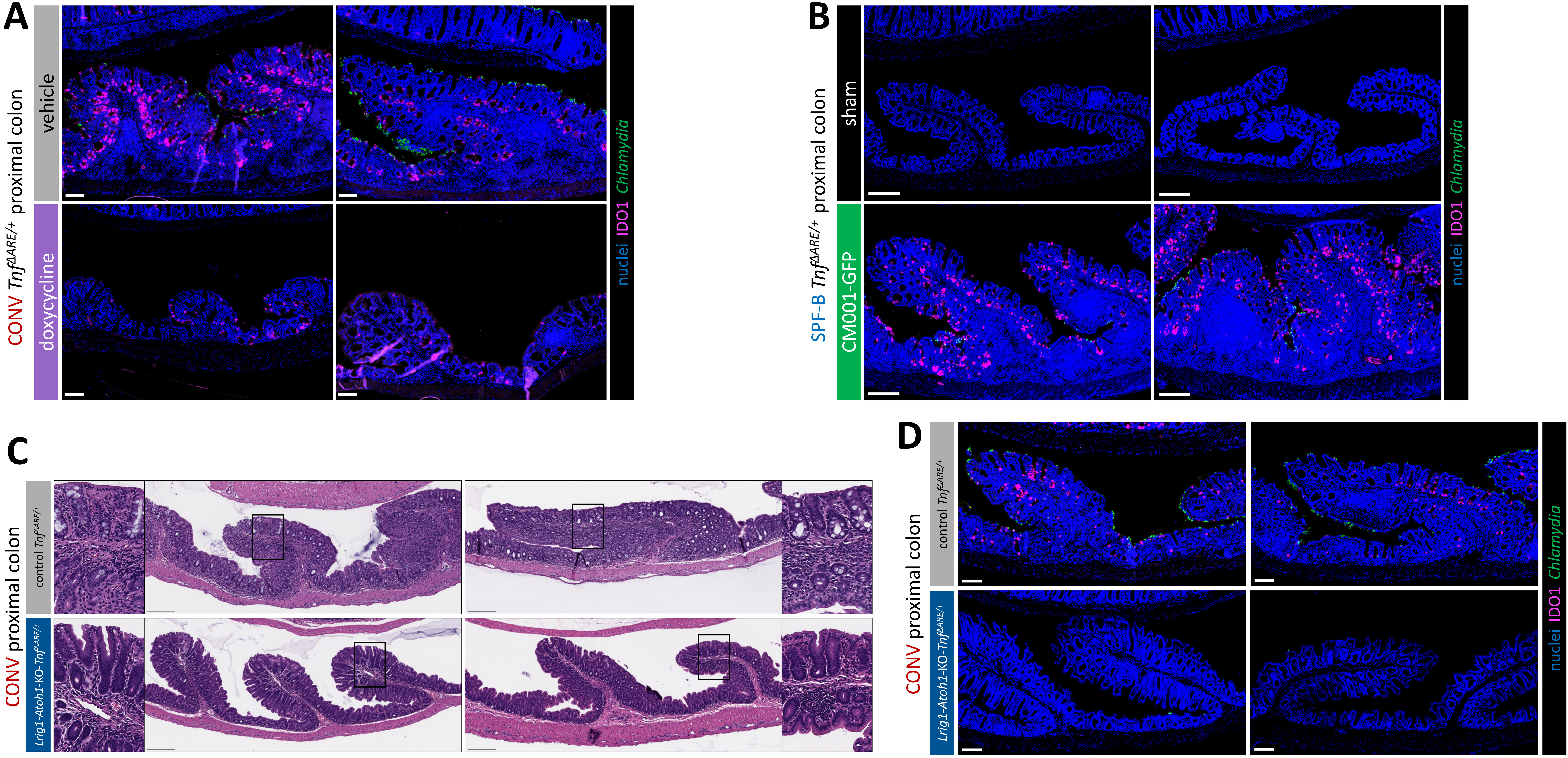

Figure S7. Secretory cell-derived IDO1 expression is associated with proximal colonic inflammation in the *Tnf*$^{\Delta ARE/+}$ model.
(A) Additional replicates of IF images of IDO1 (magenta), *Chlamydia* major outer membrane protein (MOMP - green), and nuclei (Hoechst - blue) co-staining on proximal colon sections from CONV *Tnf*$^{\Delta ARE/+}$ mice treated with doxycycline or vehicle. N = 4 mice per condition, age-matched at 11-12w of age at harvest. Scale bars = 100 µm. Data in top right and bottom left images first appear as nuclei/MOMP co-staining (no IDO1) in Figure S3A. **(B)** Additional replicates of IF images of IDO1 (magenta), *Chlamydia* major outer membrane protein (MOMP - green), and nuclei (Hoechst - blue) co-staining on proximal colon sections from SPF-B *Tnf*$^{\Delta ARE/+}$ mice that are sham or CM001-GFP-inoculated. N = 5 mice per condition, age-matched at 16-20w of age at harvest. Scale bars = 200 µm. **(C)** Additional replicates of H&E-stained proximal colon sections from *Tnf*$^{\Delta ARE/+}$ mice with or without secretory cell ablation. N = 5 control *Tnf*$^{\Delta ARE/+}$ mice, N = 3 *Lrig1-Atoh1*-KO-*Tnf*$^{\Delta ARE/+}$ mice, age-matched at 20-27w of age at harvest. Scale bars = 200 µm. Insets to show lack of goblet cell granules in the *Lrig1-Atoh1*-KO condition. **(D)** Additional replicates of IF images of IDO1 (magenta), *Chlamydia* major outer membrane protein (MOMP - green), and nuclei (Hoechst - blue) co-staining on proximal colon sections from *Tnf*$^{\Delta ARE/+}$ mice with or without secretory cell ablation. N = 5 control *Tnf*$^{\Delta ARE/+}$ mice, N = 3 *Lrig1-Atoh1*-KO-*Tnf*$^{\Delta ARE/+}$ mice, age-matched at 20-27w of age at harvest. Scale bars = 100 µm.
Related to Figure 5.

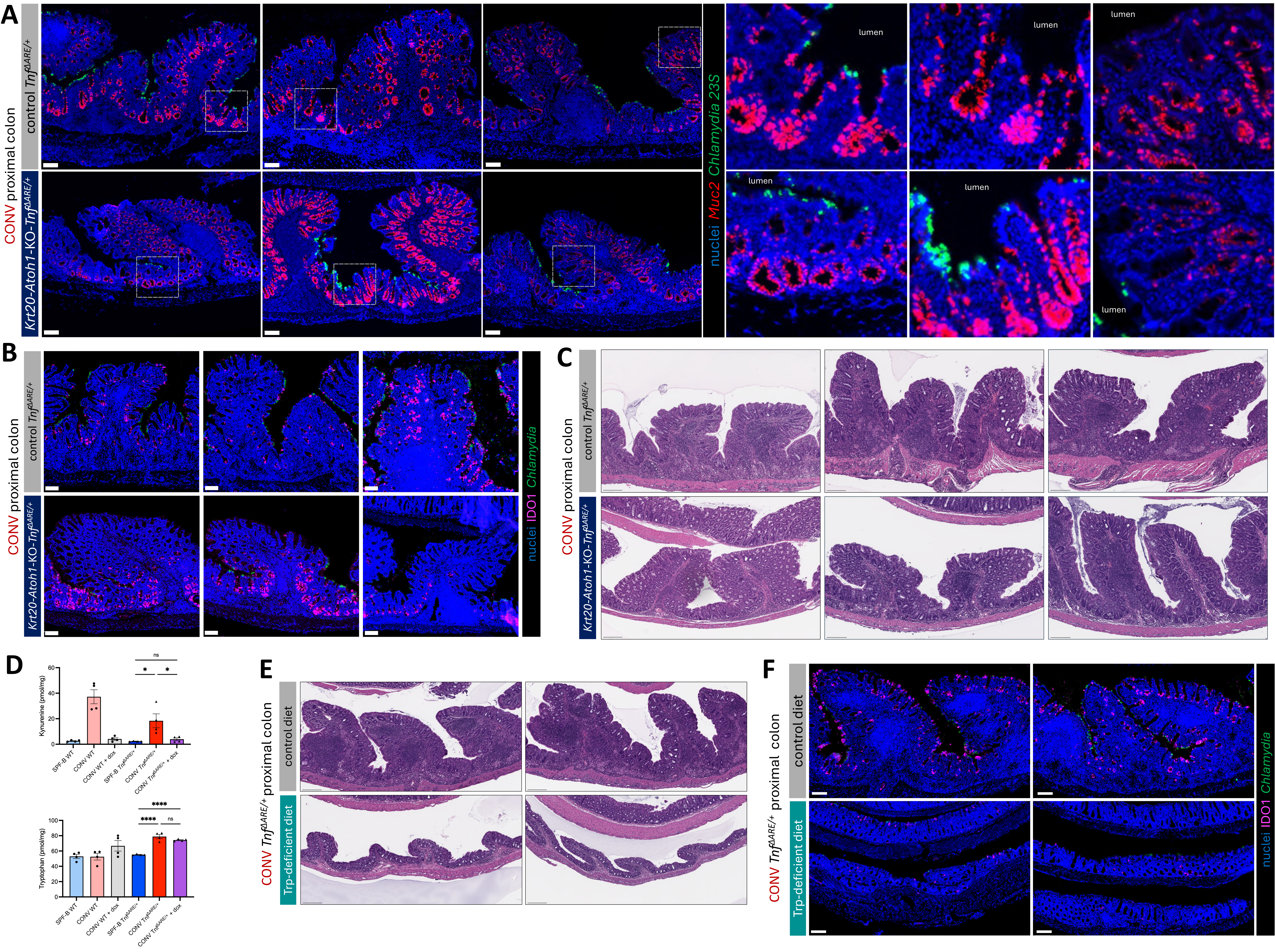

Figure S8. Goblet cell-derived IDO1 expression is associated with proximal colonic inflammation in the $Tnf^{\Delta ARE/+}$ model.
(A) Images of fluorescence *in situ* hybridization of *C. muridarum* 23S RNA (green), *Muc2* RNA (red), and nuclei (Hoechst – blue) co-staining in proximal colons of CONV $Tnf^{\Delta ARE/+}$ mice with or without differentiated secretory cell ablation. N = 4 mice per condition, age-matched (13w). Insets (right) show loss of *Muc2* expression at crypt top in *Krt20-Atoh1*-KO conditions. Scale bars = 100 µm. **(B)** Representative IF images IDO1 (magenta), *Chlamydia* major outer membrane protein (MOMP - green), and nuclei (Hoechst - blue) co-staining on proximal colon sections from CONV $Tnf^{\Delta ARE/+}$ mice with or without differentiated secretory cell ablation. N = 4 mice per condition, age-matched (13w). Scale bars = 100 µm. **(C)** H&E-stained proximal colon sections from CONV $Tnf^{\Delta ARE/+}$ mice with or without differentiated secretory cell ablation. N = 4 mice per condition, age-matched (13w). Scale bars = 200 µm. **(D)** Levels of kynurenine (top) and tryptophan (bottom) in proximal colon tissue measured by liquid chromatography mass spectrometry for the stated conditions, genotypes, and housing facilities. N = 4 per condition, age matched (13w). Mean plus SEM are shown, and statistical significance was determined using an ordinary one-way ANOVA with Sidak's multiple comparisons test. **(E)** Additional replicates of H&E-stained proximal colon sections from CONV $Tnf^{\Delta ARE/+}$ mice fed with control or tryptophan-deficient diet. N = 7 $Tnf^{\Delta ARE/+}$ mice on control diet, N = 5 $Tnf^{\Delta ARE/+}$ mice on tryptophan-deficient diet, age-matched at 11-12w of age at harvest. Scale bars = 200 µm. **(F)** Additional replicates IF images of IDO1 (magenta), *Chlamydia* major outer membrane protein (MOMP - green), and nuclei (Hoechst - blue) co-staining on proximal colon sections from CONV $Tnf^{\Delta ARE/+}$ mice fed with control or tryptophan-deficient diet. N = 7 $Tnf^{\Delta ARE/+}$ mice on control diet, N = 5 $Tnf^{\Delta ARE/+}$ mice on tryptophan-deficient diet, age-matched at 11-12w of age at harvest. Scale bars = 100 µm.
Related to Figure 5.

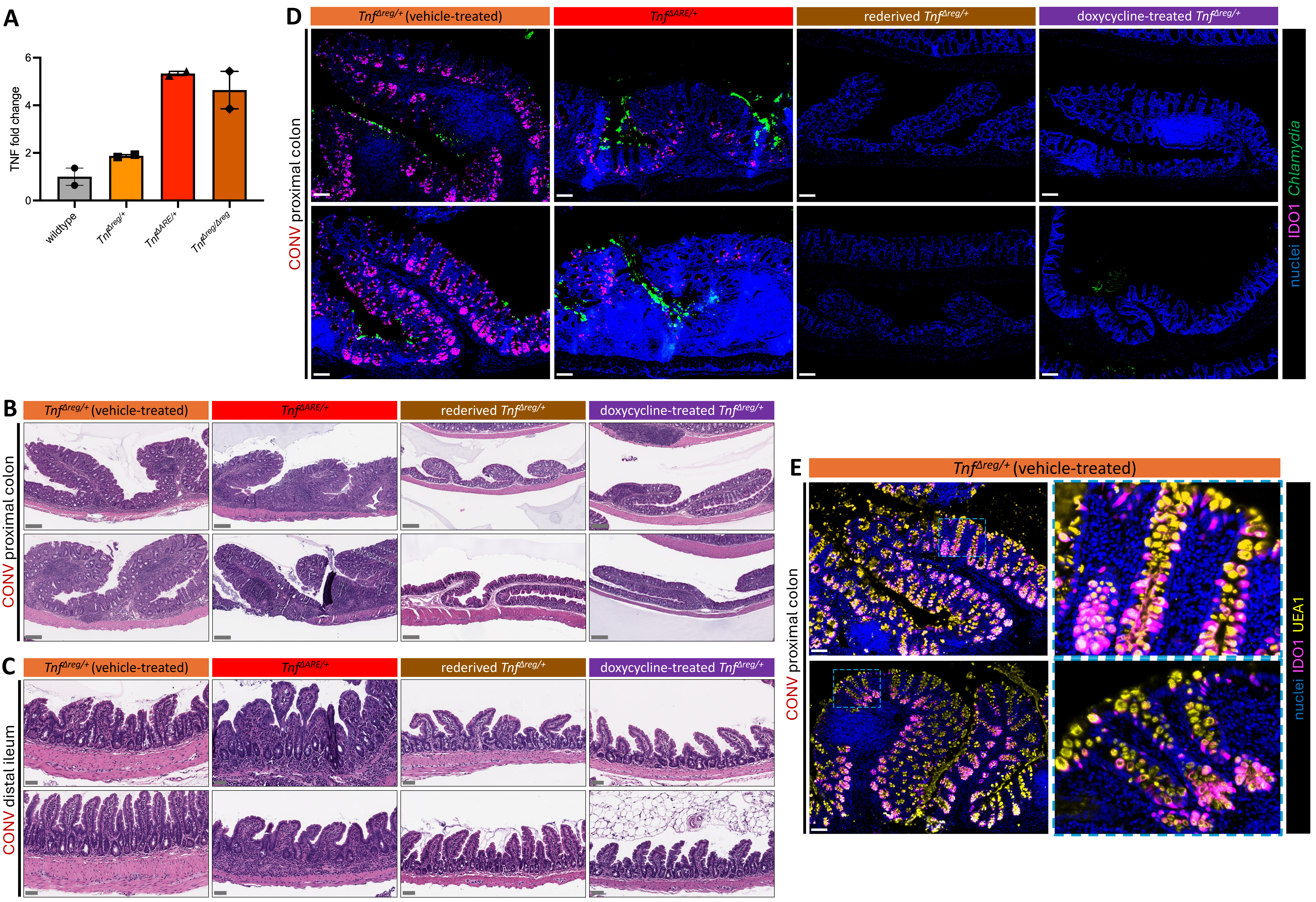

Figure S9. *Chlamydia* and TNF-driven PC inflammation is not dependent on upstream ileal inflammation.

(A) TNF protein levels, as fold change relative to wildtype, measured by ELISA from *in vitro* stimulated bone marrow derived macrophages derived from wildtype, $Tnf^{\Delta reg/+}$, $Tnf^{\Delta ARE/+}$, and $Tnf^{\Delta reg/\Delta reg}$ mice. N = 2 mice per condition aged 13-18w at harvest. All mice are from the CONV facility. **(B)** Additional replicates for H&E-stained proximal colon sections from CONV $Tnf^{\Delta reg/+}$ (N = 5), CONV $Tnf^{\Delta ARE/+}$ (N = 6), rederived Chlamydia-negative CONV $Tnf^{\Delta reg/+}$ (N = 5), and doxycycline-treated CONV $Tnf^{\Delta ARE/+}$ (N = 4) mice. Mice are age-matched at 16-17w of age at harvest. Scale bars = 200 μm. **(C)** Additional replicates for H&E-stained terminal ileum sections from CONV $Tnf^{\Delta reg/+}$ (N = 5), CONV $Tnf^{\Delta ARE/+}$ (N = 6), rederived Chlamydia-negative CONV $Tnf^{\Delta reg/+}$ (N = 5), and doxycycline-treated CONV $Tnf^{\Delta ARE/+}$ (N = 4) mice. Mice are age-matched at 16-17w of age at harvest. Scale bars = 50 μm. **(D)** Additional replicates for IF images of IDO1 (magenta), *Chlamydia* major outer membrane protein (MOMP - green), and nuclei (Hoechst - blue) co-staining of proximal colon sections from CONV $Tnf^{\Delta reg/+}$, CONV $Tnf^{\Delta ARE/+}$, rederived Chlamydia-negative CONV $Tnf^{\Delta reg/+}$, and doxycycline-treated CONV $Tnf^{\Delta ARE/+}$ mice. N = 3 mice per condition, age-matched at 16-17w of age at harvest. Scale bars = 100 μm. Data in second row, second column first appears as nuclei/IDO1/UEA1 co-staining (no MOMP) in Figure S6C. **(E)** Additional replicates for IF images of IDO1 (magenta), UEA1 lectin (yellow), and nuclei (Hoechst - blue) co-staining of proximal colon sections from CONV $Tnf^{\Delta reg/+}$ mice. Inset image to show colocalization of UEA1 lectin, a goblet and secretory granule marker, with IDO1. N = 3 mice, age-matched at 16-17w of age at harvest. Scale bars = 100 μm. Data in first row first appears as nuclei/IDO1/MOMP co-staining (no UEA1) in Figure S9D. **Related to Figure 6.**

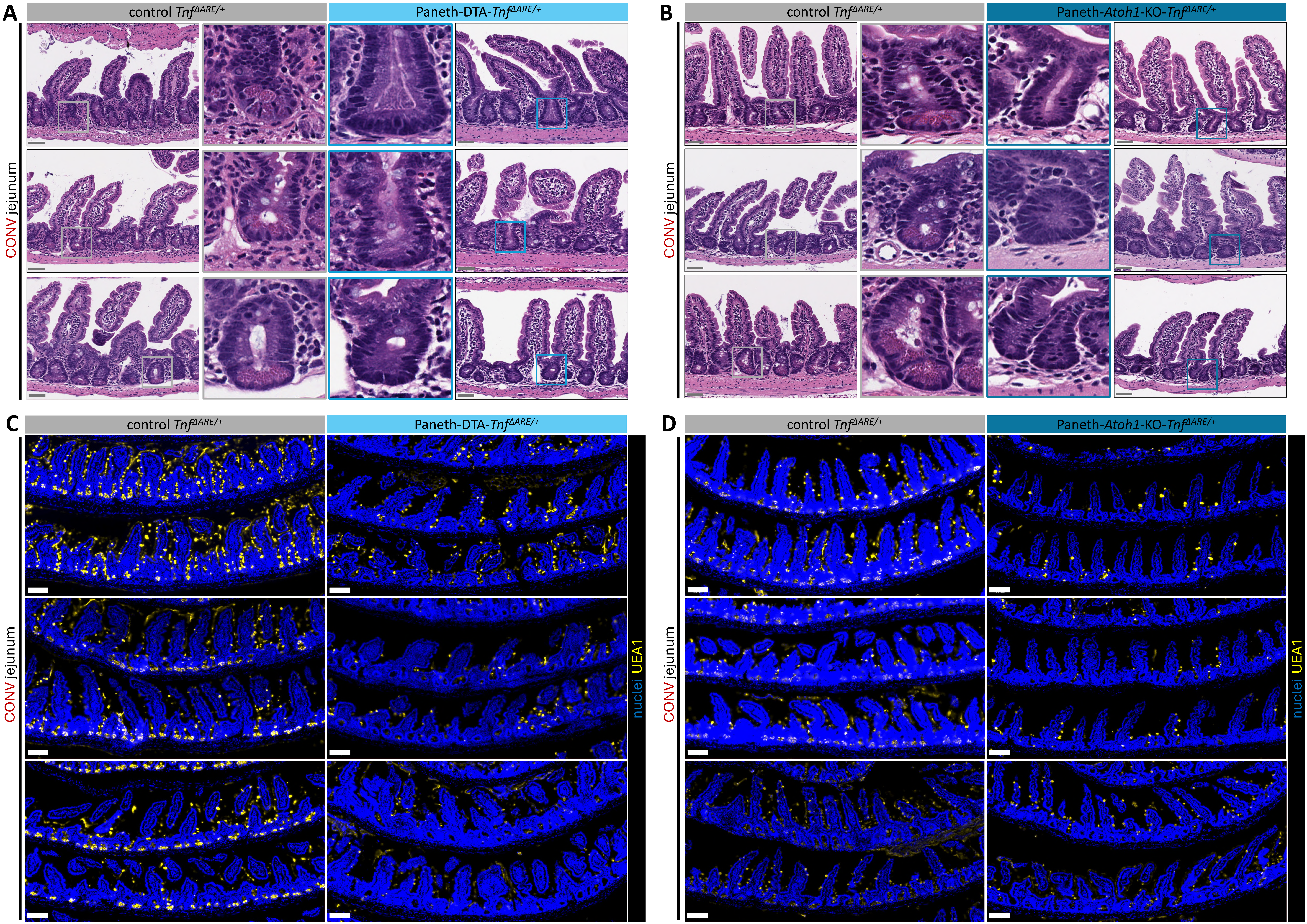

Figure S10. Paneth cells are depleted from the small intestine of Paneth-DTA-*Tnf*$^{\Delta ARE/+}$ and Paneth-*Atoh1*-KO-*Tnf*$^{\Delta ARE/+}$ mice.

(A) Representative H&E-stained jejunum sections from control *Tnf*$^{\Delta ARE/+}$ (N = 4) and Paneth-DTA-*Tnf*$^{\Delta ARE/+}$ (N = 4) mice. Insets to show lack of Paneth cell granules in crypts in the Paneth-DTA-*Tnf*$^{\Delta ARE/+}$ condition. Mice are from the CONV facility and are age-matched at 8w of age at harvest. Scale bars = 50 µm. **(B)** Representative H&E-stained jejunum sections from control *Tnf*$^{\Delta ARE/+}$ (N = 3) and Paneth-*Atoh1*-KO-*Tnf*$^{\Delta ARE/+}$ (N = 4) mice. Insets to show lack of Paneth cell granules in crypts in the Paneth-*Atoh1*-KO-*Tnf*$^{\Delta ARE/+}$ condition. Mice are from the CONV facility and are age-matched at 6-10w of age at harvest. Scale bars = 50 µm. **(C)** Representative IF images of UEA1 lectin (yellow) and nuclei (Hoechst - blue) co-staining on jejunum sections from control *Tnf*$^{\Delta ARE/+}$ (N = 4) and Paneth-DTA-*Tnf*$^{\Delta ARE/+}$ (N = 4) mice. UEA1 lectin, a Paneth and secretory granule marker, is reduced in the crypts of Paneth-DTA-*Tnf*$^{\Delta ARE/+}$ jejuna. Mice are from the CONV facility and are age-matched at 8w of age at harvest. Scale bars = 100 µm. **(D)** Representative IF images of UEA1 lectin (yellow) and nuclei (Hoechst - blue) co-staining on jejunum sections from control *Tnf*$^{\Delta ARE/+}$ (N = 4) and Paneth-*Atoh1*-KO-*Tnf*$^{\Delta ARE/+}$ (N = 4) mice. UEA1 lectin, a Paneth and secretory granule marker, is reduced in the crypts of Paneth-*Atoh1*-KO-*Tnf*$^{\Delta ARE/+}$ jejuna. Mice are from the CONV facility and are age-matched at 6-10w of age at harvest. Scale bars = 100 µm.

Related to Figure 6.

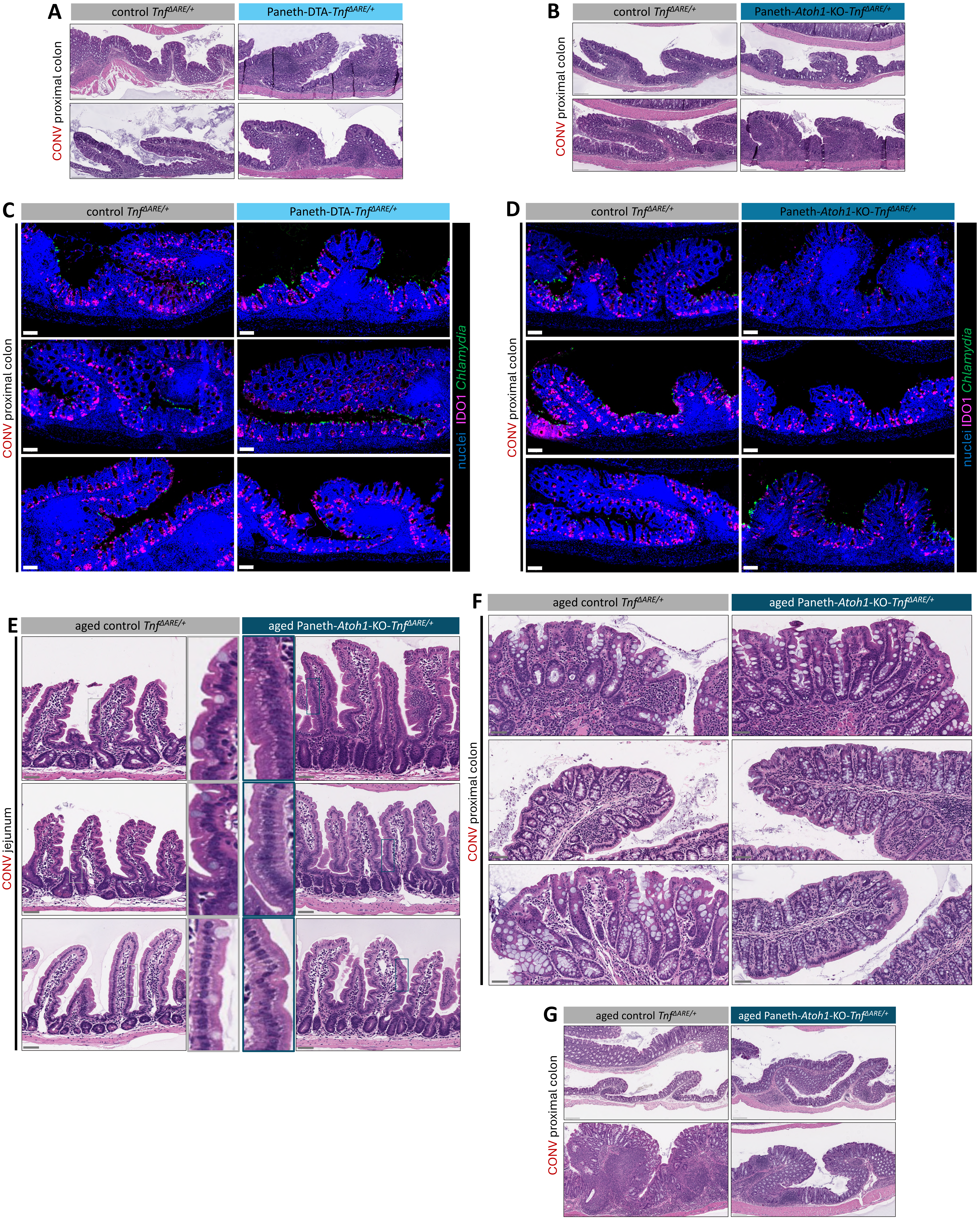

Figure S11. Paneth cell depletion neither promotes nor protects against proximal colonic inflammation in the $Tnf^{\Delta ARE/+}$ model.
(A) Additional replicates for H&E-stained proximal colon sections from control $Tnf^{\Delta ARE/+}$ (N = 4) and Paneth-DTA-$Tnf^{\Delta ARE/+}$ (N = 4) mice. Mice are from the CONV facility and are age-matched at 8w of age at harvest. Scale bars = 200 µm. **(B)** Additional replicates for H&E-stained proximal colon sections from control $Tnf^{\Delta ARE/+}$ (N = 3) and Paneth-*Atoh1*-KO-$Tnf^{\Delta ARE/+}$ (N = 4) mice. Mice are from the CONV facility and are age-matched at 6-10w of age at harvest. Scale bars = 200 µm. **(C)** IF images of IDO1 (magenta), *Chlamydia* major outer membrane protein (MOMP - green), and nuclei (Hoechst - blue) co-staining on proximal colon sections from control $Tnf^{\Delta ARE/+}$ (N = 4) and Paneth-DTA-$Tnf^{\Delta ARE/+}$ (N = 4) mice. Mice are from the CONV facility and are age-matched at 8w of age at harvest. Scale bars = 100 µm. **(D)** IF images of IDO1 (magenta), *Chlamydia* major outer membrane protein (MOMP - green), and nuclei (Hoechst - blue) co-staining on proximal colon sections from control $Tnf^{\Delta ARE/+}$ (N = 3) and Paneth-*Atoh1*-KO-$Tnf^{\Delta ARE/+}$ (N = 4) mice. Mice are from the CONV facility and are age-matched at 6-10w of age at harvest. Scale bars = 100 µm. **(E)** Representative H&E-stained jejunum sections from aged control $Tnf^{\Delta ARE/+}$ (N = 4) and aged Paneth-*Atoh1*-KO-$Tnf^{\Delta ARE/+}$ (N = 4) mice. Mice are from the CONV facility and are age-matched at 23-64w of age at harvest. Scale bars = 50 µm. **(F)** Representative H&E-stained proximal colon sections from aged control $Tnf^{\Delta ARE/+}$ (N = 4) and aged Paneth-*Atoh1*-KO-$Tnf^{\Delta ARE/+}$ (N = 4) mice. Mice are from the CONV facility and are age-matched at 23-64w of age at harvest. Scale bars = 50 µm. **(G)** Additional replicates for H&E-stained proximal colon sections from aged control $Tnf^{\Delta ARE/+}$ (N = 4) and aged Paneth-*Atoh1*-KO-$Tnf^{\Delta ARE/+}$ (N = 4) mice. Mice are from the CONV facility and are age-matched at 23-64w of age at harvest. Scale bars = 200 µm.
Related to Figure 6.

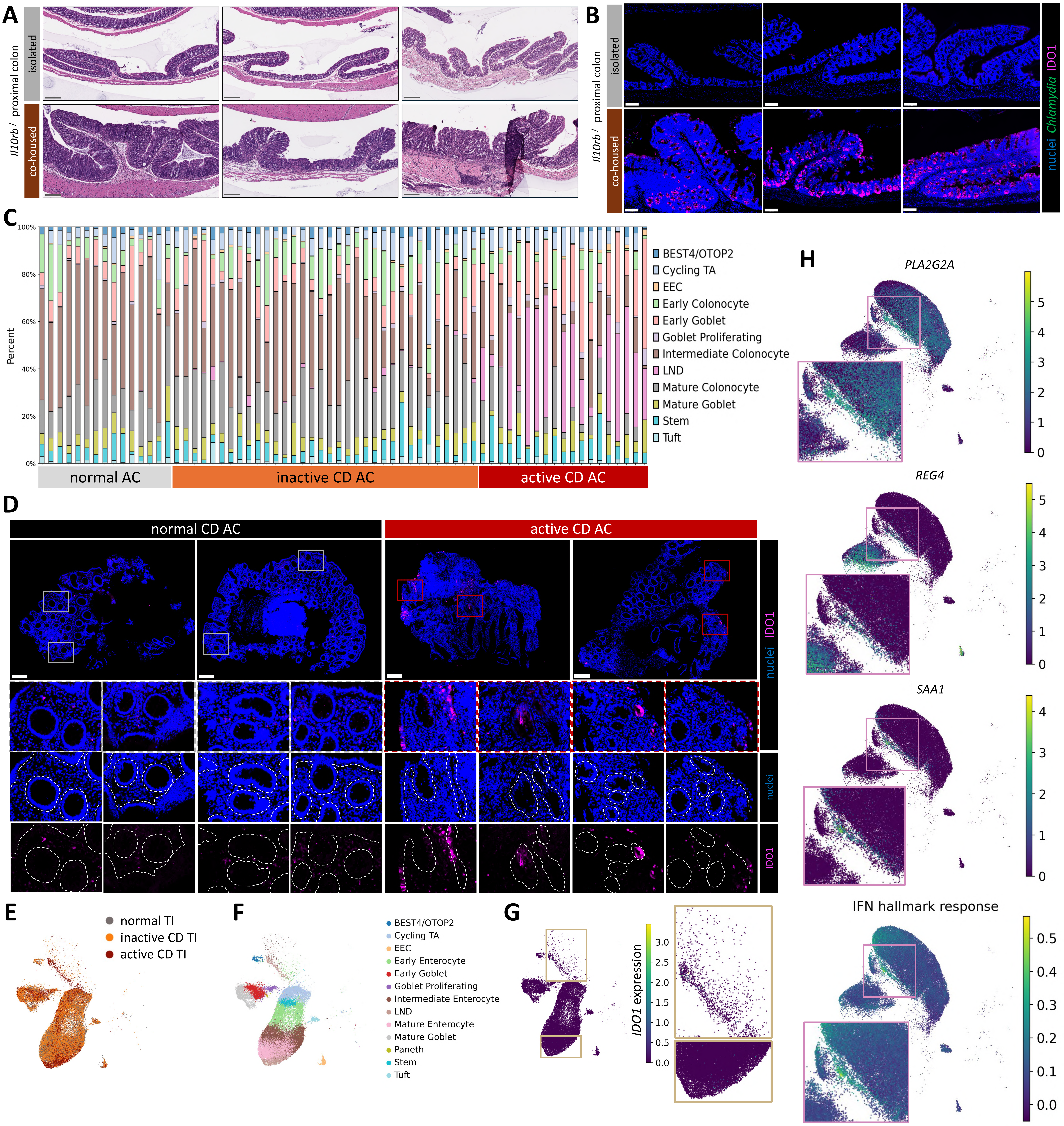

Figure S12. CD specimens with active AC inflammation are associated with upregulation of IDO1 in epithelial cells.
(A) H&E-stained proximal colon sections from *Il10rb$^{-/-}$* mice in isolated caging or *Chlamydia*-positive co-housing conditions. N = 3 mice per condition, age-matched ranging 22-47w of age at harvest. Co-housing conditions were for 4-8 weeks. Scale bars = 200 µm. **(B)** IF images of IDO1 (magenta), *Chlamydia* major outer membrane protein (green), and nuclei (Hoechst - blue) co-staining on proximal colon sections from *Il10rb$^{-/-}$* mice in isolated caging or *Chlamydia*-positive co-housing conditions. N = 3 mice, age-matched ranging 22-47w of age at harvest. Scale bars = 100 µm. **(C)** Barplot with epithelial cell type proportion for each specimen, grouped by sample type, of scRNA-seq data of AC samples. **(D)** Additional replicates of IF images of IDO1 (magenta) and nuclei (Hoechst - blue) co-staining on ascending colon biopsies from CD specimens with normal (N = 3) or active (N = 3) histopathological scoring of the AC. Inset and individual channels to show IDO1 expression in epithelial cells. Dashed white lines indicate the basal side of epithelial cells. Scale bars = 200 µm. **(E)** UMAP co-embedding of scRNA-seq samples of TI epithelial cells from normal (N = 15), inactive CD (N = 30), and active CD (N = 17) specimens. Sample type overlay is indicated by color. Normal TI is composed of healthy control specimens, inactive CD TI is composed of CD specimens histopathologically scored as normal or quiescent, and active CD TI is composed of CD specimens histopathologically scored as mild, moderate, or severe. **(F)** UMAP of TI scRNA-seq data with cell type overlay indicated by color (left) and LND cell overlay indicated by color (right). **(G)** UMAP of TI scRNA-seq data with overlay of *IDO1* gene expression indicated by the color gradient. **(H)** UMAP of AC scRNA-seq data with overlay of gene expression or hallmark IFN-γ response metagene score indicated by the color gradient.
Related to Fig 7.
